# 3D photogrammetry quantifies the size of basal cell carcinoma lesions with submillimeter accuracy: high correlation with lesion response to photodynamic therapy

**DOI:** 10.1117/1.JBO.30.S3.S34107

**Published:** 2025-09-25

**Authors:** Edward V. Maytin, Nathalie C. Zeitouni, Abigail Updyke, Jeffrey T. Negrey, Alan S. Shen, Lauren E. Heusinkveld, Sanjay Anand, Christine B. Warren, Tayyaba Hasan, Brian W. Pogue

**Affiliations:** a Cleveland Clinic, Department of Dermatology, Cleveland, Ohio, United States; b Cleveland Clinic, Department of Biomedical Engineering, Cleveland, Ohio, United States; c Cleveland Clinic Lerner College of Medicine of Case Western Reserve University, Cleveland, Ohio, United States; d Massachusetts General Hospital, Wellman Center for Photomedicine, Boston, Massachusetts, United States; e University of Arizona College of Medicine, Medical Dermatology Specialists, Phoenix, Arizona, United States; f Cleveland Clinic, Clinical Research Unit, Cleveland, Ohio, United States; g Dartmouth College, Thayer School of Engineering, Hanover, New Hampshire, United States

**Keywords:** photogrammetry, imaging systems, image analysis

## Abstract

**Significance:**

Noninvasive imaging to accurately measure subtle changes in tumor size is underutilized when assessing therapeutic responses in the skin. During photodynamic therapy (PDT) for basal cell carcinoma (BCC), a better definition of the tumor size threshold for PDT responsiveness is needed.

**Aim:**

We aim to quantitatively demonstrate the first clinical evidence of tumor shrinkage after multiple rounds of PDT using a robust measurement and analysis approach.

**Approach:**

Tumors were monitored experimentally using a 3D camera and software system (stereo photogrammetry). A total of 122 BCC tumors in 35 patients were treated with PDT (5-ALA and blue light) in three sessions. Calculated volumes and heights were used to plot changes in tumor size.

**Results:**

In total, 70% of BCC cleared completely. Measured heights correlated with histological tumor depth; average heights were ∼10% to 20% of actual tumor depth. From photogrammetry at baseline, an average height of <0.15  mm was found to predict a complete therapeutic response. Thus, our 3D morphometric technique provides a surrogate measure of BCC tumor depth that predicts PDT response and is accurate to well below the millimeter level.

**Conclusions:**

3D photogrammetry can inform the selection of BCC tumors for PDT with exceptionally high spatial accuracy, dramatically better than can be quantified by a clinician.

## 1 Introduction

Skin cancers as a group are the most common cancers known to man, i.e., the incidence of basal cell carcinoma (BCC) and squamous cell carcinoma (SCC) exceeds all other forms of cancer, including breast and colon cancer.^1^ Although surgery is often used to treat skin cancers, another option for BCC and SCC is to use medical therapies such as fluorouracil cream, imiquimod cream, or photodynamic therapy (PDT).^2^ PDT is a combination treatment in which a topical photosensitizing drug is applied to the tumor and subsequently activated using visible light illumination;^3^ it is approved for treatment of BCC in 18 European countries but not yet in the United States.^4^ When conducting research studies on the clinical response to medical therapies, it is very important to accurately monitor changes in tumor size as a function of time. To date, the vast majority of clinical studies in dermato-oncology have used tumor area, estimated visually by a study physician measuring the length and width of the tumor, as the primary endpoint. Occasionally, clinical trials will report tumor volumes that were calculated by multiplying tumor area by a tumor height (distance above the normal skin surface) that was visually estimated by study investigators.^5^ Unfortunately, these visual methods tend to be highly unreliable. A more objective method for quantifying the height and volume of skin tumors at a near-microscopic scale (i.e., with submillimeter accuracy) is needed.

Photogrammetry is the science of obtaining reliable information about physical objects through recording, measuring, and interpreting photographic images; this includes the extraction of three-dimensional measurements from two-dimensional data (i.e., images). Here, we describe a photogrammetric technique that employs a 3D camera system and software to generate kinetic profiles for individual skin tumors as they respond to PDT. In a clinical trial of patients with multiple BCC tumors, a large tumor cohort (122 BCC lesions) was treated and followed using photogrammetry for an extended period. Each tumor was treated repeatedly with PDT (three treatment sessions, spaced 2 months apart), and the tumor size was monitored via (1) clinical size estimation, i.e., by a study physician using a millimeter ruler; and (2) the new 3D photogrammetric method. Results show that 3D photogrammetry can provide kinetic data that quantify tumor growth and/or shrinkage over a prolonged therapeutic time course, with a submillimeter accuracy that is impossible to achieve using clinical size estimation alone.

## 2 Materials and Methods

### 2.1 3D Camera and Software

**Fig. 1:**
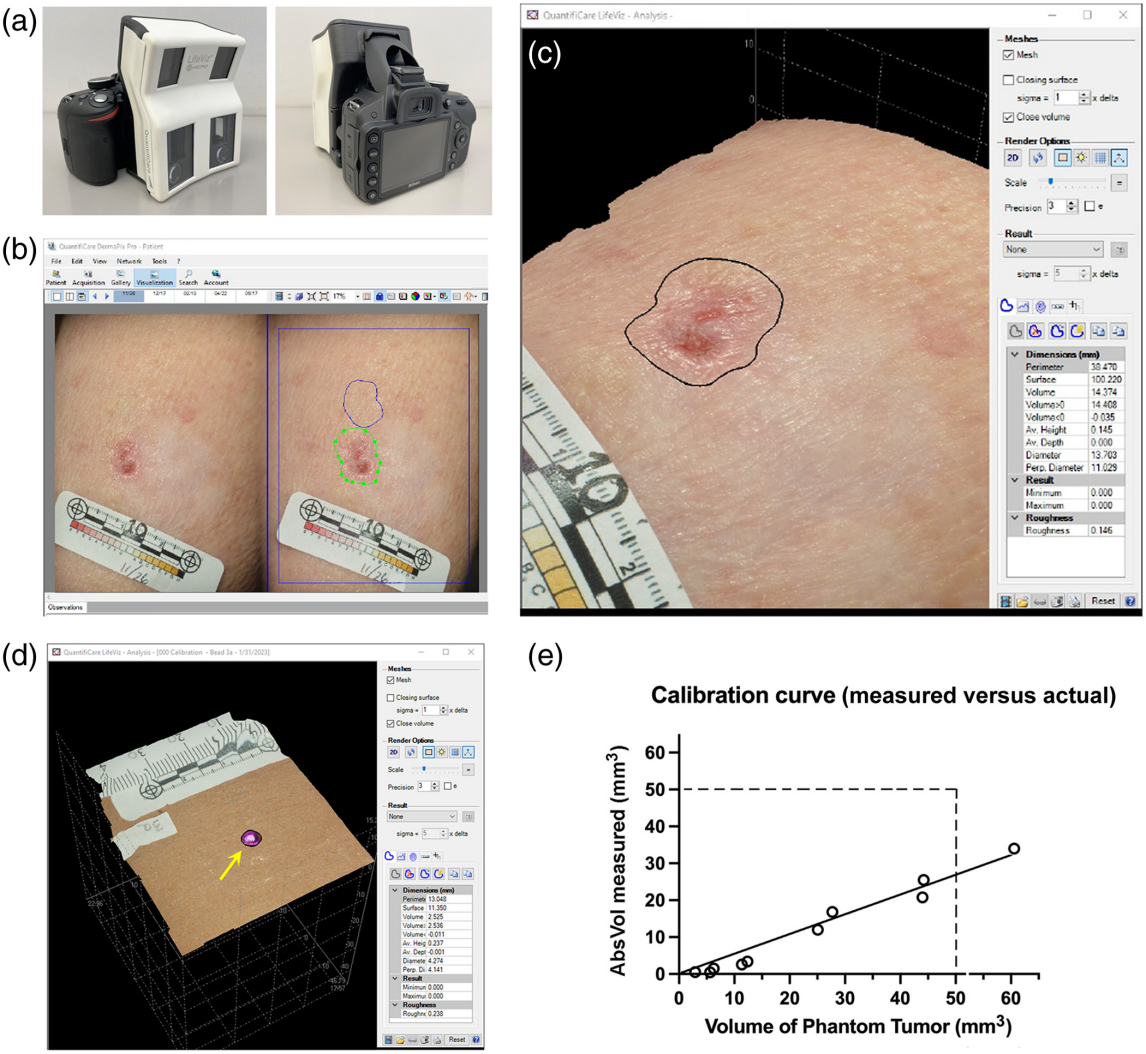
Features of the 3D camera and software system, including data output and calibration. (a) Dual lens assembly mounted on a Nikon camera body. (b) Appearance of the pair of images after being imported into the DermaPix software program. Images are displayed here in 2D mode. The *green line* shows a ROI, drawn by the investigator, which encompasses the BCC tumor. The *blue line* outlines an area of normal skin, which is an ROI used to take a background (nontumor) reading. (c) The same BCC tumor, now visualized in 3D mode. Note the raised edges, central ulceration, and disruption of normal skin lines. The paper sticker with tick marks (millimeter ruler) was placed onto the patient’s skin prior to taking the photographs. (d) Tumor phantom (purple epoxy bead, indicated by *yellow arrow*), as visualized in the 3D analysis window. (e) Calibration curve, showing values of 3D*AbsVol* versus the actual volume of the epoxy phantoms.

The LifeViz Micro camera^6^ consists of a standard digital camera body outfitted with a special housing that contains mirrors and dual lenses spaced ∼5  cm apart to simulate stereo vision [Fig. 1(a)]. With this camera, the researcher simultaneously captures a pair of digital photographs of the tumor. The dual images are then transferred to a laptop computer running the 3D image analysis program (DermaPix, by Quantificare Inc., Valbonne, France). After importing the pair of images into the analysis program, the pair of images are displayed side-by-side in two-dimensional mode [Fig. 1(b)]. One can toggle back and forth between the 2D drawing mode [Fig. 1(b)] and 3D visualization mode in which the tumor is displayed as a 3D image [Fig. 1(c)]. The 3D image can be rotated in space and viewed from all angles, and can also be magnified to show very fine details such as skin lines, raised borders, and small ulcerated areas within the tumor, as seen in Fig. 1(c).

To perform quantitative analysis, the investigator defines the region of interest (ROI) to be analyzed by drawing an outline around the tumor [the *green* line in Fig. 1(b) and the *black* line in Fig. 1(c)]. The computer program then converts the 3D image into a 3D mesh network from which various measurements within the ROI can be calculated. In the example in Fig. 1(c), the ROI is defined as the black outline drawn around the tumor (window on the *left*). Within the ROI, various parameters are automatically measured and displayed within the report window [Fig. 1(c), *lower right*]. These parameters include *surface area*, major axis diameter, minor axis (perpendicular) diameter, positive volume (above the surface plane), negative volume (below the surface plane), maximum lesion height, average lesion height, and “roughness”; the definitions of these parameters are given in the Supplementary Material. In developing our data, we empirically tried each parameter during analysis and ultimately found two of them to be the most consistent and useful for describing changes in tumor size after PDT. The first was *absolute volume* (**3DAbsVol**), defined as the sum of the positive volume and the absolute value of the negative volume. The second parameter was *average lesion height* (**3DAvHt**). From the report window, parameters of interest can be exported to an Excel spreadsheet for further analysis.

### 2.2 Calibration of 3D Absolute Volumes Using Known Size Standards

The analysis system (3D camera and associated software) is pre-calibrated by the vendor to generate numerical outputs in millimeter units (for lengths), and other calculated parameters in either ${\mathrm{mm}}^{2}$ (for areas) or ${\mathrm{mm}}^{3}$ (for volumes). To estimate the true relationship between tumor volume and the measured 3DAbsVol, the following experiment was conducted. First, BCC tumor phantoms were created using decorative beads (made of epoxy resin and shaped as half-spheres of various sizes), purchased from a local hobby store. Each bead was weighed on a laboratory balance (Mettler), and then adhered to a piece of cardboard to create a BCC tumor phantom. Each of the beads was photographed using the 3D camera, and their 3DAbsVol were calculated using the analysis software as illustrated in Fig. 1(d). The calculated 3DAbsVol values were plotted against the actual volumes of the standards, assuming the density of epoxy to be 1.2  mg/mL (or $1.2\;\mathrm{mg}/{\mathrm{mm}}^{3}$). The plot in Fig. 1(e) demonstrates a linear relationship between 3DAbsVol and actual volume. Although the calculated 3DAbsVol of our phantoms appears to be ∼50% less than the actual volume, the fact that complex 3D mesh calculations can introduce some systemic inaccuracy is expected. For our clinical study, we made no attempt to adjust the 3D output values because we were interested in relative changes rather than absolute measurements of BCC tumor size.

### 2.3 Clinical Trial Design

The clinical trial was registered at ClinicalTrials.gov (NCT03483441) and conducted at two different sites, Cleveland Clinic (Cleveland, Ohio) and US Dermatology Partners (Phoenix, Arizona) under related but separate IRB approvals. For inclusion, all patients were required to have a minimum number of biopsy-proven BCC tumors (2 or 3, depending upon the trial location), and up to 10 lesions per patient could be studied. Two or three histologically proven BCC were required for enrollment at the Cleveland site, or Arizona site, respectively. Exclusion criteria included pregnancy, history of renal disease or porphyria, and treatment with vismodegib or vitamin D supplementation within the past month.

The study consisted of five study visits, each spaced 2 months apart. The first visit was for baseline measurements; the second, third, and fourth visits were for PDT treatments; and the last visit was for final lesion counts. 3D photos of BCC lesions were taken at every visit, and immediately prior to PDT at visits 2, 3, and 4. The primary aim of the clinical study was to test whether high doses of oral vitamin D, when used in combination with PDT, may affect (accelerate) the kinetic clearance rate of BCC tumors. For that purpose, patients were randomized to take high-dose vitamin D for 1 week prior to either visits 2 or 3, to see what effect this might have upon the kinetics (rate of shrinkage) of BCC tumors subsequently. Because the high-dose vitamin D intervention at visits 2 or 3 ultimately had only small effects upon tumor clearance (data not shown), and due to the paper length required for an adequate description of complex vitamin D effects, the full details about the vitamin D aspects of clinical trial NCT03483441 will be presented in a separate paper.

### 2.4 PDT Treatments

PDT treatments were administered as follows. A solution of 20% ALA (Levulan Kerastick, Sun Pharmaceuticals, Mumbai, India) was applied to each tumor and a 5 mm rim of surrounding skin, covered with an occlusive dressing, and 4 h later illuminated with blue light (Blu-U lamp, 417 nm; $20\;J/{\mathrm{cm}}^{2}$; Sun/DUSA) as described.^7^ Pain relief was provided using ice-cold cloths as needed. Patients were sent home with instructions to avoid direct sun exposure for 48 h.

### 2.5 Evaluation of BCC Histologic Subtypes

For each biopsied lesion, the histological diagnosis was determined by a board-certified dermatopathologist at the respective institutions. To analyze the depth of BCC lesions, extra slides were recut from the original paraffin blocks, stained with hematoxylin and eosin (H&E), and the maximum depth (distance from epidermis to deepest aspect of tumor nests) was analyzed using QuPath software.^8^

## 3 Results

All raw and calculated measurements of BCC tumors obtained during the clinical trial are provided in Table S1 in the Supplementary Material. As described in Sec. 2, patients were recruited at two different clinical sites (14 patients at Cleveland Clinic and 22 patients at the Arizona site). Of 36 total subjects enrolled, 35 completed the entire protocol. Patients were generally older adults (mean age, 62 years), Caucasian, and male (70%). A total of 211 lesions were monitored using 3D photogrammetry during the trial. Among these, 89 were later excluded due to a subsequent non-BCC diagnosis (SCC; fibrosis/scar) or to technical issues that precluded a complete analysis at all five study visits (see Table S1 in the Supplementary Material). Thus, 122 lesions were ultimately available for a complete analysis during the entire 6-month study time course.

### 3.1 Description of Quantitative Data Obtainable from 3D Photogrammetry of BCC Skin Tumors

**Fig. 2:**
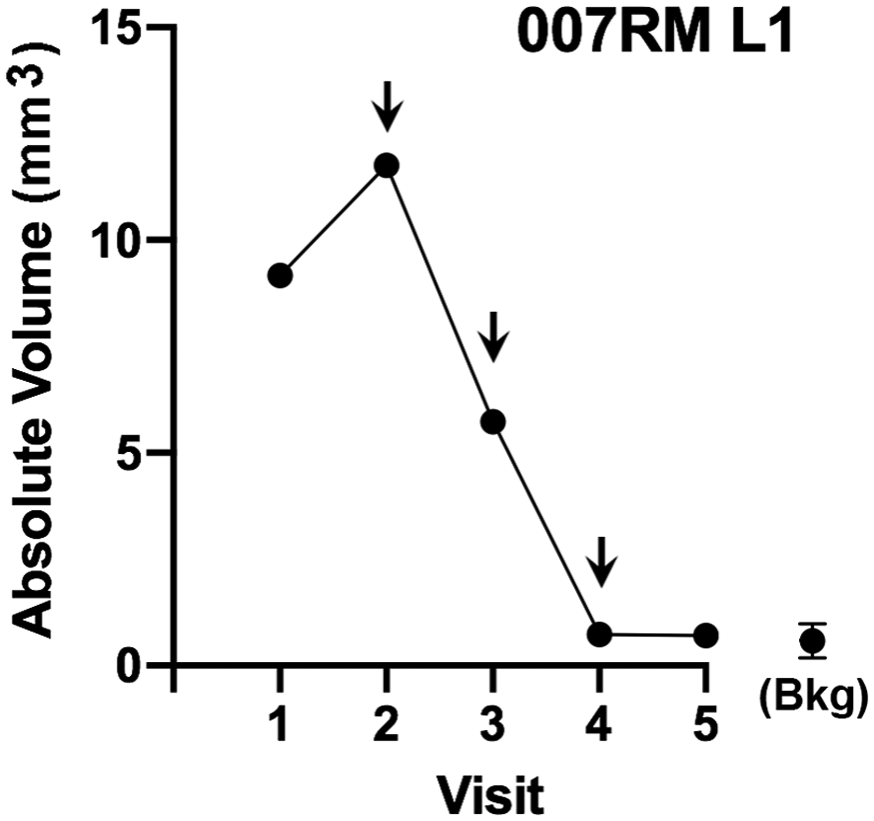
Kinetics of the change in size of a BCC tumor in response to PDT (this example is from patient 7, lesion 1). The data for 3DAbsVol of the lesion listed in Table 1 are displayed graphically here. *Downward arrows* indicate PDT treatment visits. Bkg, background (normal skin).

As described in Sec. 2, three-dimensional images (mathematical reconstructions) of tumors from dual high-resolution photographs were used to determine 3DAbsVol and 3DAvHt for each tumor, relative to the skin’s horizontal surface plane. Table 1 shows an example of data from one BCC lesion (in this case, lesion 1, patient 7). When comparing the first two rows of data (height and the perpendicular diameters of the lesion as estimated by the study physician during clinical examination, *rows 1–2*), the 3D photographic method clearly provides a more precise measurement for these parameters (*rows 3–5*). The 3DAvHt parameter, obtained by dividing absolute volume by lesion area, is particularly noteworthy as it provides a quantitative estimate of height, which is not possible to do from clinical examination alone. For describing changes in tumor size during a treatment time course, the absolute volume (3DAbsVol) is the most informative as it can detect small changes in size relative to the surface plane; the latter is represented by 3DAbsVol background measurements taken within regions of normal skin surrounding the tumor (*row 8*). To establish a quantitative definition for lesion disappearance, one can set the 3DAbsVol background (mean ± SD, *row 9*) as a threshold for defining lesion disappearance. By plotting the 3DAbsVol data, the resulting graph serves as a convenient visual record of tumor behavior in response to PDT (Fig. 2). Looking at Fig. 2, this lesion continued to grow after the first study visit (no treatment), but after the first PDT treatment (visit 2), the trend was reversed and the lesion partially shrank. After another PDT treatment (visit 3), the volume of the tumor reached the background level of normal skin by visit 4. A third PDT treatment (visit 4) had no further effect on the lesion, so we interpret this as a complete clearance response at visit 4 after PDT. In this particular case, a skin biopsy was performed at visit 5, and this confirmed the absence of residual tumor.

**Table 1:** Example of 3D photogrammetric data obtained for a BCC lesion.

	Patient-lesion: 007-L1	Visit number				
		V1	V2	V3	V4	V5
1	Clinical height estimate	Raised	Raised	Slightly raised	Slightly raised	Flat
2	Clinical diameter × 2 (mm)	8 × 10	8 × 10	5 × 10	8 × 4	2 × 2
3	3D diameter (mm)	12.9	11.4	7.7	7.9	5.9
4	3D perpendicular diameter (mm)	10.1	7.4	3.5	4.6	4.2
5	3D average height (mm)	0.06	0.07	0.03	0.01	0.01
6	3D volume (${\mathrm{mm}}^{3}$)	9.11	11.70	5.64	0.23	-0.25
7	3D absolute volume (${\mathrm{mm}}^{3}$)	9.17	11.76	5.73	0.73	0.70
8	3D absolute volume, background readings	0.44	0.59	n/d	n/d	0.73
9	3D absolute volume, mean ± SD	0.59 ± 0.15				
10	BCC lesion response	----- Lesion cleared at V4 ------				

### 3.2 Photogrammetric Analysis of Changes in Tumor Size After PDT: Overall Patterns and Results

Values for 3DAbsVol and 3DAvHt, obtained from the 3D photos of each lesion at every study visit, were calculated as described in Table 1 and Fig. 2 and listed in Table S1 in the Supplementary Material for all tumors. From this, we were able to quantitatively determine how each BCC tumor responded to PDT and ask which tumor characteristics (e.g., overall size, thickness, and histological features that relate to biologic behavior) might correlate with PDT response.

**Fig. 3:**
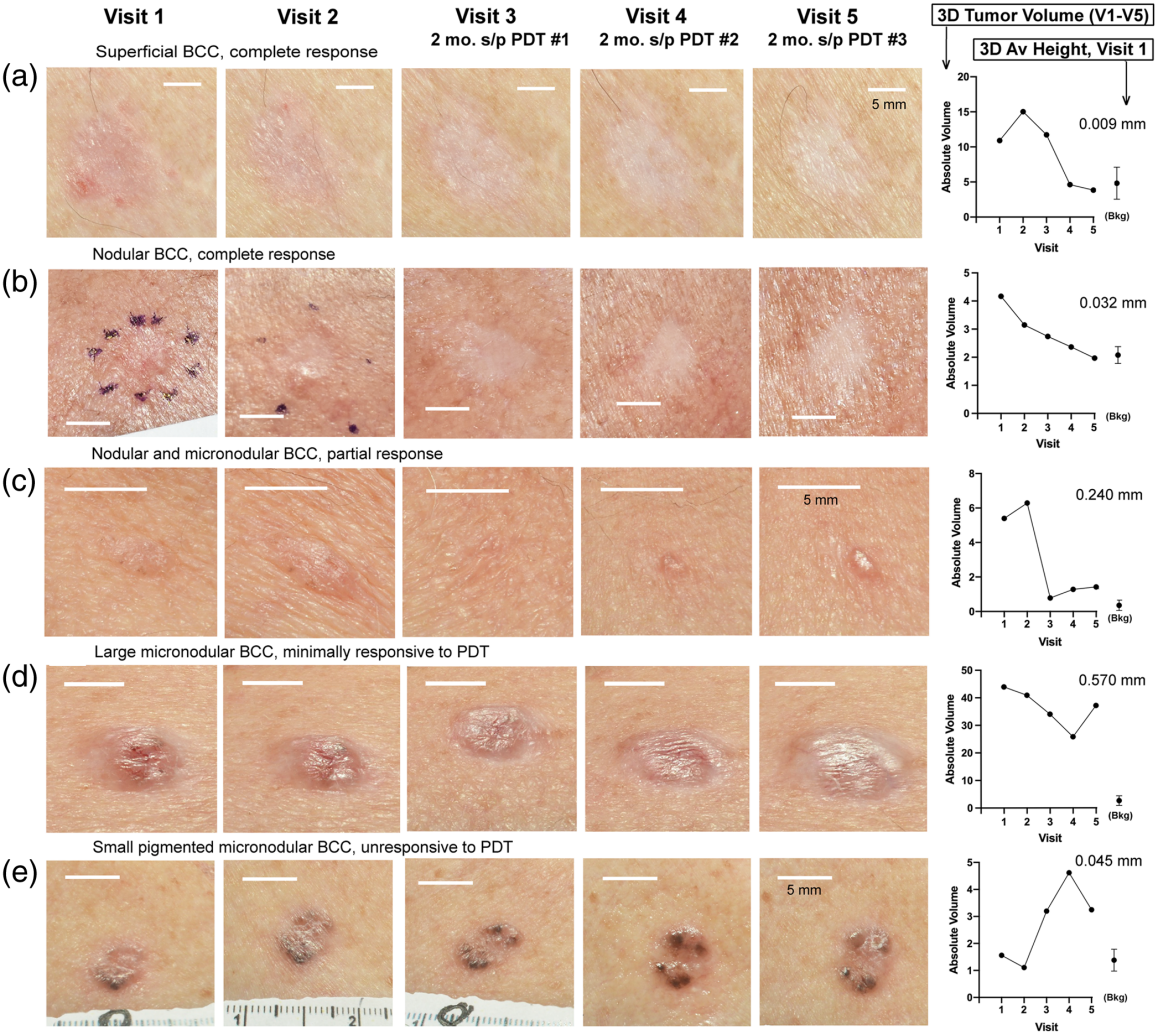
3D reconstructed images of BCC tumors photographed at each of the five study visits. Photographic images (*left*) are screenshots of 3D reconstructions to show typical appearances of each tumor at multiple visits. Graphs (at right) show the corresponding changes in calculated tumor volumes (*3DAbsVol*, in ${\mathrm{mm}}^{3}$) of each tumor, over the time course of 5 study visits. The 3D average height (*3DAvHt*, in millimeters) of each tumor at visit 1 is indicated in the upper right corner of the graphs. Scale bars, 5 mm. (a) Superficial BCC, complete response; (b) nodular BCC, complete response; (c) nodular and micronodular BCC, partial response; (d) large micronodular BCC, minimally responsive to PDT; and (e) small pigmented micronodular BCC, unresponsive to PDT.

Overall, the majority of BCC lesions study resolved after PDT; 41% of tumors were cleared by visit 3, 58% by visit 4, and 70% by visit 5. Conversely, 30% of the tumors failed to clear and were therefore classified as PDT-resistant. At a more granular level, one can begin to examine patterns and relationships by comparing the photographic appearance of each tumor with its clearance kinetics, as illustrated in Fig. 3. In general, most BCC that cleared with PDT were small, relatively thin tumors [Figs. 3(a) and 3(b)]. By histological examination, most of these were superficial BCC [Fig. 3(a)], but some had a nodular histology [Fig. 3(b)]. Some tumors responded only transiently to PDT, shrinking after one or more treatments, but then growing back [Figs. 3(c) and 3(d)].

A very important general result of this clinical trial is that tumor height really matters for therapeutic outcomes. Thus, the calculated height (3DAvHt) was typically greater for PDT-resistant lesions than for PDT-responsive ones, as illustrated in Figs. 3(a)–3(d). However, as discussed in more detail below, notable exceptions to the inverse correlation between tumor height and PDT responsiveness were seen with BCC tumors with an aggressive histological subtype. An example of the latter is shown in Fig. 3(e), where there is no inhibition in tumor growth rate post-PDT, despite the lesion being relatively thin (3DAvHt = 0.045 mm).

### 3.3 Quantification of the Influence of BCC Tumor Size Upon PDT Responsiveness

**Fig. 4:**
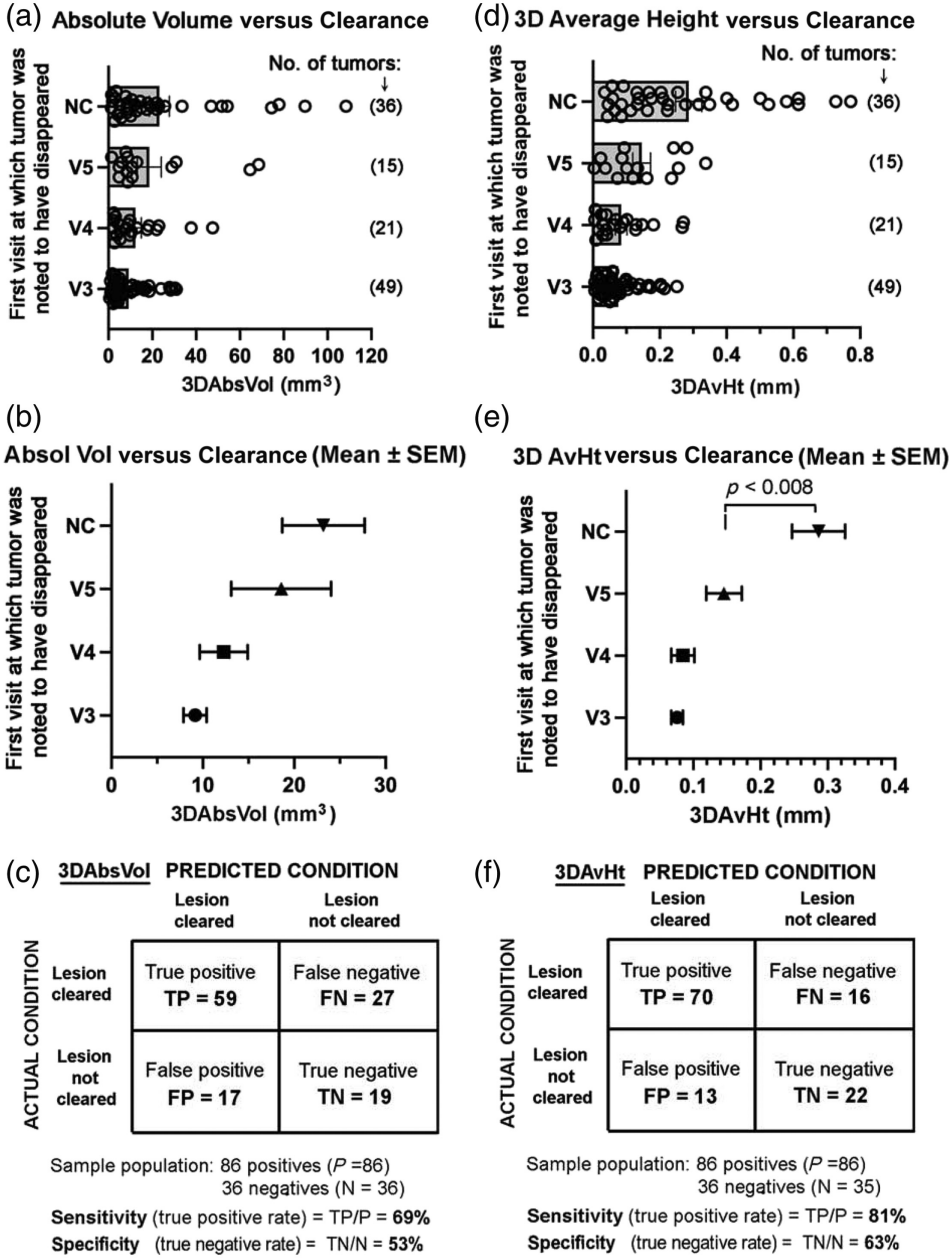
Correlation study among calculated size parameters from 3D analysis of BCC tumors *versus* tumor clearance, as indicated by the disappearance of tumor at visits V3, V4, or V5 (after 1, 2, or 3 PDT treatments, respectively). *NC*, tumors that failed to clear after 3 PDT treatments. (a)–(c): *3D absolute volume* is shown as data for individual tumors (a), or in aggregate (b). The confusion plot in panel (c) shows calculated sensitivity and specificity for 3DAbsVol measurements (see Table S2 in the Supplementary Material for details). (d)–(f): *3D average height* is shown as data for individual tumors (d), or in aggregate (e). The confusion plot in panel (f) shows calculated sensitivity and specificity for the 3DAvHt measurements (see Table S3 in the Supplementary Material for details).

To more precisely define the influence of tumor volume and thickness upon therapeutic response, we focused on two size parameters, *absolute volume* (3DAbsVol) and *3D average height* (3DAvHt) (see Table S1 in the Supplementary Material for a complete listing of these values for all tumors). We used the change in 3DAbsVol of the tumor relative to normal surrounding skin as our primary definition of tumor response (as described in Table 1 and Fig. 2). Tumors that cleared completely using this definition were grouped by the visit where their disappearance was first noticed (either V3, V4, or V5). Tumors that persisted at V5 were classified as *not cleared* (NC). Considering all evaluable tumors, a direct relationship between 3DAbsVol [Figs. 4(a) and 4(b)], or 3DAvHt [Figs. 4(d) and 4(e)], and the number of PDT treatments required to clear the tumor was observed. The volume measurements (3DAbsVol) were able to predict the actual PDT-induced clearance of tumors with a sensitivity of 69% and a specificity of 53% [Fig. 4(c)], and the height parameter (3DAvHt) yielded even better sensitivity (81%) and specificity (62%) values [Fig. 4(f)]. Specificity estimates could be improved further by recognizing that a majority of PDT nonresponders in our study, most of which had not been pre-diagnosed by biopsy, were histologically aggressive BCC subtypes. If those tumors are eliminated from the analysis (by assuming that in clinical practice, the aggressive types of BCC will be pre-identified by biopsy and therefore excluded from undergoing PDT), then the specificity values for 3DAbsVol and 3DAvHt rise to 70% (see Tables S2 and S3 in the Supplementary Material).

Interestingly, a 3DAbsVol value of $12\;{\mathrm{mm}}^{3}$, or a 3DAvHt value of ∼0.15  mm, appears to represent useful operational thresholds at which sensitivity and specificity are approximately equal. Tumors below the threshold are less likely to respond to PDT, compared with tumors larger than the threshold. As a predictor of BCC lesion clearance, we favor 3DAvHt because it is conceptually simpler and has better specificity (81%) and specificity (70%) characteristics (see Table S3 in the Supplementary Material).

**Fig. 5:**
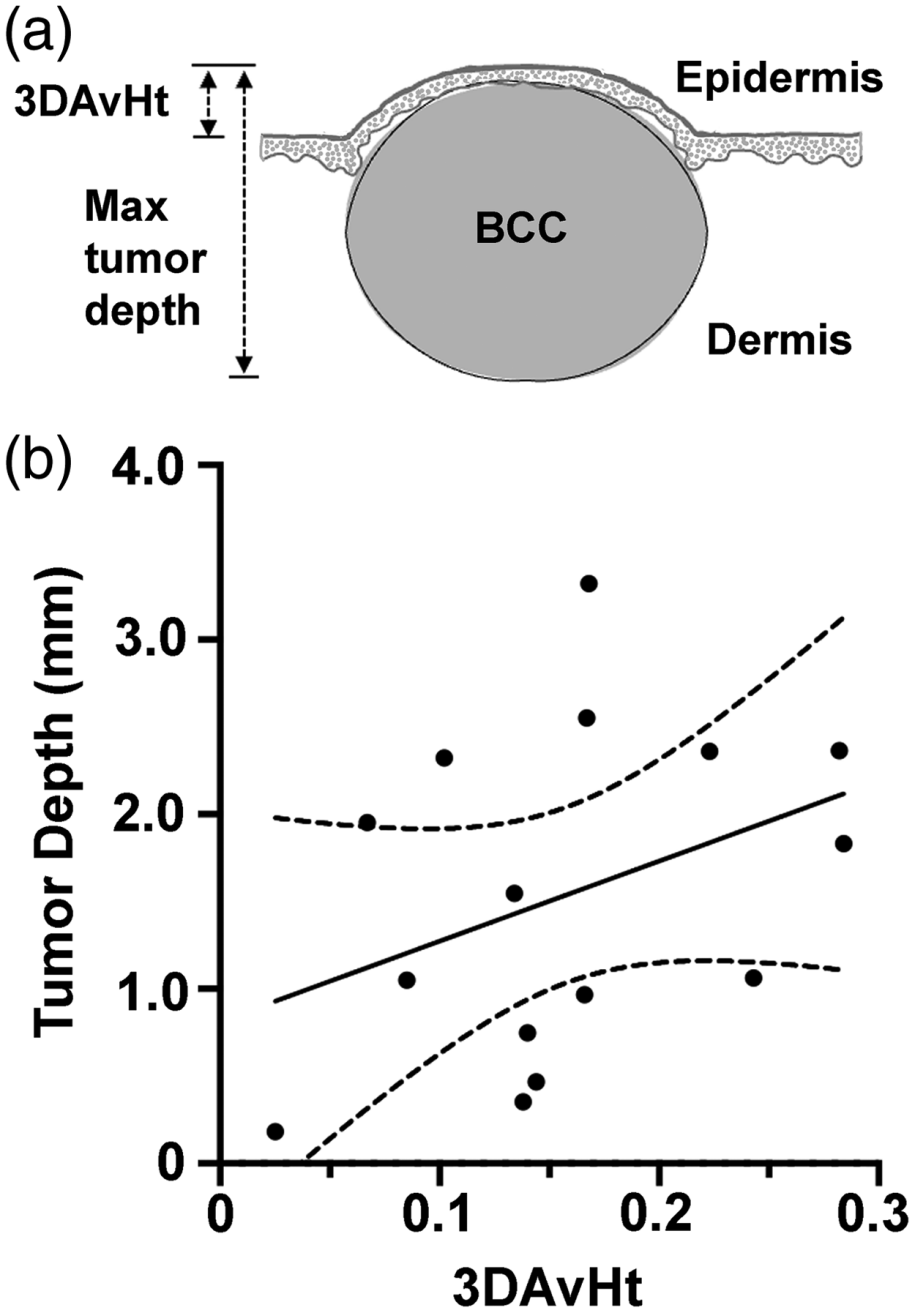
(a) Schematic diagram showing the definitions of 3DAvHt and histological tumor depth. (b) Plot to show the modest correlation between 3DAvHt and histologic depth for a subset of tumors that were biopsied at the same visit. Maximum depth of tumor nests was measured from digital images of H&E-stained histological sections; depths are in millimeters.

To understand how 3DAvHt might relate to the true intradermal depth of the tumor [as 3DAvHt is a calculated height that may or may not be related to actual histological tumor depth; see Fig. 5(a)], we performed the following analysis. For 15 BCC tumors that were photographed immediately prior to biopsy at V5, the 3DAvHt was compared with the depth of tumor nests observed in the corresponding H&E stained specimen. A roughly linear correlation between 3DAvHt and a maximum depth of the tumor was observed [Fig. 5(b)]. Although 3DAvHt values were only ∼10% to 20% of the actual tumor depth, a positive correlation between the two parameters was seen, even when tumors were stratified by histological subtype (Table S4 in the Supplementary Material). Thus, we conclude that 3DAvHt represents a surrogate measure of dermal depth for these BCC tumors.

## 4 Discussion

When investigating the efficacy of skin cancer treatments, accurate methods for monitoring tumor response to the therapeutic agent are essential. BCC tumors can be difficult to assess quantitatively because they vary widely in shape (flat, nodular, or ulcerated) and size (centimeters to millimeters in diameter). Unfortunately, traditional measurements for estimating tumor size (visual estimation with a ruler or calipers or from 2-D photographs) are very unreliable. In a prior study of BCC tumors, we found that the use of 2-D photography to assess post-PDT responses^7^ was only useful as a qualitative (descriptive) indicator. We therefore sought to develop an improved method that can quantitatively detect subtle changes in the size (volume and height) of small skin tumors. Specifically, we wished to accurately measure tumor volumes to ask how the size and depth of the tumor might correlate with response to PDT therapy. This has been an important unresolved question in the PDT field for some time now.^5^^,^^9^

During the planning of the clinical trial, we considered existing options for 3D measurement of skin tumors. Several methods are currently available for performing photogrammetry of the skin, including the use of stereo-multiplexed cameras, hyperspectral imaging, and confocal imaging (reviewed in Ref. 10). However, most of these systems are designed for observations at a macro scale, e.g., for clinical applications such as esthetic treatment planning prior to cosmetic surgery of the face,^11^^–^^13^ or for creating *ex vivo* phantom models of skin tumors prior to radiation therapy.^14^^,^^15^ Although some devices can extract depth information, e.g., to characterize wrinkles or acne scars,^16^ most are not designed to analyze cutaneous features at a submillimeter level. An exception is line-field confocal optical coherence tomography (LC-OCT), which provides impressive details of cutaneous structure at the near-cellular level (reviewed in Ref. 17). However, LC-OCT requires funding and expertise well beyond the limited research budget available for our clinical trial. Therefore, we developed a 3D photogrammetric technique that uses a commercially available, handheld 3D camera system and analytic software from Quantificare that can provide a convenient point-and-shoot approach in the clinic and allow people to perform lesion analysis with minimal training. Thus, in terms of innovation, the relative value of our paper lies in demonstrating how an already available equipment package can be used for photogrammetry at submillimeter accuracy when evaluating BCC skin cancer in patients.

The data reported here, from our systematic examination of 122 BCC tumors monitored over a 6-month treatment time course, show that a relatively simple 3D stereo photogrammetric system can be used to quantitatively characterize biological responses of individual BCC tumors. This capability is important not just for descriptive purposes but also for providing new functional insights. Specifically, 3D photographic morphometry allowed us to demonstrate for the first time that calculated tumor volumes and heights can be used to define a size threshold that discriminates between PDT-responsive and PDT-resistant tumors. This finding has very important clinical implications as it will allow physicians to better select which tumors to treat with PDT to achieve a successful therapeutic outcome.

The calculated parameter (3DAvHt), which proved to be the most useful predictor of PDT response, was found to correlate with the depth of BCC tumor nests below the surface, even though 3DAvHt represents only ∼10% to 20% of the actual tumor depth [Fig. 4(e)]. The critical threshold value for 3DAvHt [0.20 mm; see Fig. 4(d)] corresponds to an approximate tumor depth of 1.5 mm [Fig. 4(F)], which matches quite closely to results from a clinical study by Mosterd et al.^18^ that compared PDT outcomes with BCC histological tumor depth. In that study, PDT responses of 78 BCC tumors with a wide range of thickness values (0.30 to 3.10 mm) were categorized into two groups with widely different outcomes, i.e., tumors <1.3  mm thick had a risk of treatment failure of 15.5%, whereas tumors >1.3  mm thick had a risk of treatment failure of 42.2% % (P=0.09). Their 1.3 mm depth threshold^18^ and our estimated 1.5 mm depth threshold are very similar. Importantly, these values are consistent with European guidelines recommending that PDT be performed only for BCC<2  mm in depth.^19^^,^^20^ Our new 3D photographic method represents an objective, noninvasive way of judging whether a BCC tumor falls within these established clinical guidelines.

The recommendation of 2 mm as a depth threshold originated with a pioneering study by Morton et al.^9^ of 53 BCC tumors, which demonstrated that thickness significantly affected PDT response, with no response in four patients whose tumors exceeded 2 mm in thickness. Subsequently, most PDT clinical trials have focused on patients with BCC tumors < 2 mm thick, primarily superficial BCC but some with nodular BCC as well.^21^ As a result, many clinicians who perform PDT have learned to visually estimate tumor depth with a surprising degree of accuracy but the ability to correctly predict the histological BCC subtype is relatively poor, i.e., only 72% in a recent large study.^5^ Surface diameter can be indicative of BCC depth, but only for large tumors. In 235 biopsies of relatively large nodular BCC (mean surface diameter 13.0±8  mm), Takenouchi et al.^22^ found that horizontal surface diameter correlated with and was the strongest predictor of tumor depth, followed by histologic subtype. Our new 3D morphometry technique permits a relative depth evaluation of any BCC tumor, whether large or small, which could be quite helpful for PDT screening purposes. That said, any BCC tumor that is large or has clinically suspicious features should be histologically evaluated before undergoing PDT.

An important and fascinating question is how 3DAvHt is able to predict PDT responses when most of the tumor lies below the surface. Here, we need to consider the concept of “photodynamic priming,” in which only a portion of a tumor needs to be damaged by PDT to trigger local events (expression of damage-associated molecular patterns, alteration of tumor stroma, stimulation of innate immune cells) that eventually lead to activation of systemic anti-tumor immunity.^23^^–^^26^ Light penetration into the skin is controlled by the laws of physics, and therefore photodynamic efficiency of light at various depths within the skin can be modeled and predicted.^27^ Combining this idea with the concept of photodynamic priming, we postulate that 3DAvHt (a parameter proportionately related to histological tumor depth) is useful because it correlates with the light penetration depth required to successfully induce photodynamic priming.

The above discussion about tumor thickness should not obscure the fact that the BCC histological subtype is an overriding determinant of PDT responsiveness. Four BCC growth patterns are thought to have prognostic significance post-treatment: superficial, nodular, micronodular, and infiltrative.^28^ In a study of BCC skin biopsies by Welsch et al.,^29^ superficial (23%) and nodular (59%) subtypes were most prevalent, whereas micronodular (13%) and infiltrative (5%) subtypes were less common. This compares well with our data in which the distribution of subtypes was superficial (31%), nodular (44%), micronodular (15%), infiltrative (5%), and other (7%). Micronodular and infiltrative subtypes are often reported as the thickest tumors and the most likely to recur, and indeed these were the deepest and most frequent histologic subtypes in our dataset (Table S4 in the Supplementary Material, Graph A).

One limitation of this study was the fairly large number of data points that were unavailable for analysis due to unanticipated problems. For example, at the Arizona site, many BCC lesions (44%) that were initially photographed at visit 1 had disappeared by visit 2, even before PDT was administered; this was probably due to inflammation from the initial diagnostic skin biopsy. At the Cleveland site, a biopsy was not required for all lesions at the outset, and as a consequence, ∼9% of lesions were clinically misdiagnosed and later proved to be scars, keloids, or SCC tumors. Finally, some digital photos were missing or had too many hairs within the image, which disrupted the software mesh analysis during 3D reconstruction.

In summary, we have described a robust, noninvasive, 3D photogrammetric measurement technique that can accurately measure changes in BCC tumor size at the submillimeter level. Because this noninvasive technique (1) provides a surrogate measure of actual tumor size, (2) predicts therapeutic responsiveness, (3) uses relatively simple equipment (a handheld camera and a laptop for running the software), and (4) is relatively easily to perform, the method should be useful in dermatology research and clinical settings to help identify which BCC are appropriate candidates for successful PDT.

## Supplementary Material

10.1117/1.JBO.30.S3.S34107.s01

## Data Availability

All data in support of the findings of this paper are available within the article or as the Supplementary Material.

## ClinicalTrials.gov

NCT03483441.
