# Supplementary material for: 3D photogrammetry quantifies the size of basal cell carcinoma lesions with submillimeter accuracy: high correlation with lesion response to photodynamic therapy
Source: J Biomed Opt. 2025 Sep 25;30(Suppl 3):S34107. doi: 10.1117/1.JBO.30.S3.S34107 (PMC12463384; doi:10.1117/1.JBO.30.S3.S34107)
Supplement: Supplementary file 1 [file JBO_030_S34107_SD001.pdf]

SUPPLEMENTARY MATERIALS

for Maytin et al, JBO manuscript

Supplem. Methods.	Definitions of 3D analytic parameters used in 3D photogrammetry. (2 pages)
Supplem. Table 1.	Kinetic response data for all BCC lesions analyzed in the clinical trial. (23 pages)
Supplem. Table 2.	Tumor volume (3DAbsVol) as a predictor of BCC clearance after PDT: Data and calculations of sensitivity and specificity. (2 pages)
Supplem. Table 3.	Tumor height (3DAvHt) as a predictor of BCC clearance after PDT: Data and calculations of sensitivity and specificity. (2 pages)
Supplem. Table 4.	Comparisons of BCC histologic subtype, measured tumor depth, and calculated height (3DAvHt). (1 page)

SUPPLEMENTARY METHODS

Definitions of 3D analytic parameters generated by the Dermapix software from Quantificare, Inc.

A. Concept of a “closing surface” as the reference point for 3D Analysis:

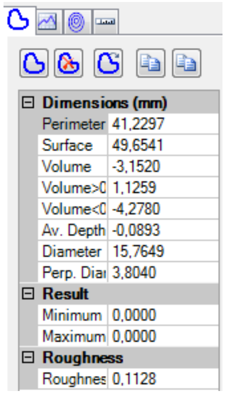

With the Quantificare analysis package, the image is first rendered as a 3-D network of multiple points (a “mesh”). This is then used to generate a variety of calculated parameters, including lesion height (above the surface), lesion depth (below the surface), positive lesion volume (‘mountains’), or negative lesion volume (‘valleys’). Each of these is calculated relative to a baseline reference surface (closing surface).

The measured depths/heights and volumes are a difference between the skin surface and a reference surface (the closing surface). This reference is a smoothed abstract representation of the skin (an average). The value "sigma" is a smoothing coefficient for this surface, shown below in white:

Sigma 2	Sigma 4	Sigma 6	...	Sigma 99 (flat surface)
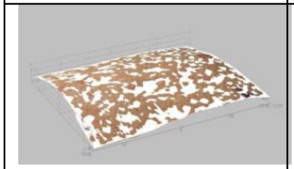	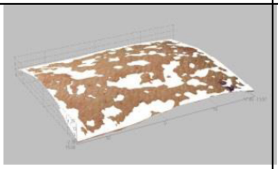	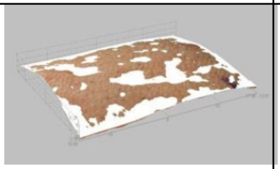		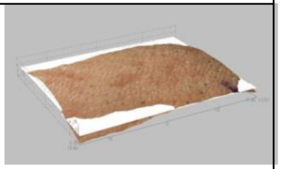

- ✓ A small sigma indicates small unevenness (lesions, folds, pimples).
- ✓ A higher sigma indicates larger unevenness.

It is important to understand that the “closing surface” is defined by the global region used for 3D reconstruction. In the ideal case, the reconstructed surface is flat with one area of unevenness (such as a bump –e.g. a *raised tumor;*- or a hole eg., a *wrinkle* or an *ulcer*).

B. Values measured in the 3D Analysis

Perimeter (in mm): Circumference of the lesion (this is seldom used in clinical trials).

Surface area (in mm2): Real 3D surface area of the lesion (useful for comparing the evolution of a lesion between two visits).

***Volume**:* (in mm3): Total volume of the lesion using a minimal surface based on lesion boundaries to close the volume of the region for such a computation. We then evaluate the **negative volume** (under the closing surface), the **positive volume** (above the closing surface) and the **total volume** (equal to positive plus negative volume). (volume = volume>0 - volume<0).

Absolute Volume: (in mm3): same as the total volume (equal to positive plus negative volume). (volume = volume>0 - volume<0). We call this the 3D Absolute Volume (**3DAbsVol**) in the manuscript.

The diagram below represents the surface of the skin. The pink color represents the positive volume. The blue color represents the negative volume. In red we have the baseline calculated by the software.

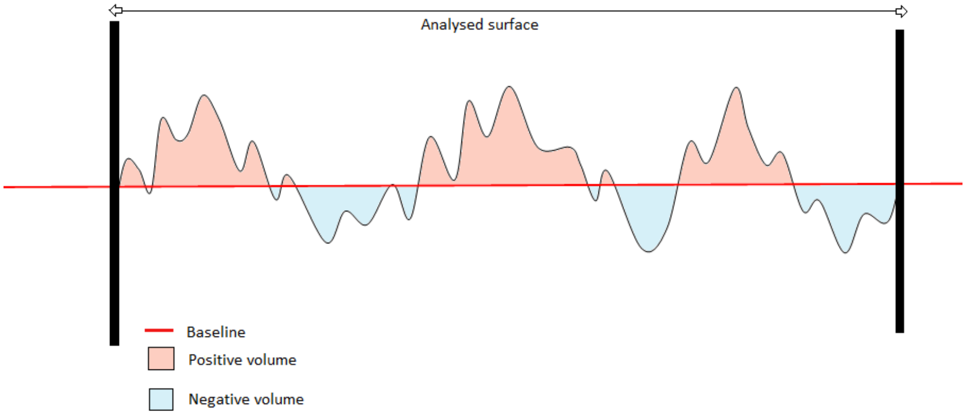

Avg. Depth: (in mm); also known as ***Avg. Height:*** (in mm). This parameter is equal to the total volume (= positive volume – negative volume), divided by the surface. It is useful, for example to characterize a wrinkle because it is a more stable parameter than volume or surface. Average depth is a value based on a contour (the contour of the wrinkle for example). In the Quantificare program, although this parameter is named "depth" (because it was set up to characterize wrinkles), it actually represents a "height" since it is based on the difference between positive and negative volumes. In the manuscript, we refer to this as the 3D Average Height **(3DAvHt).**

Diameter / Perpendicular diameter: The diameter is the measurement of a straight line drawn between the widest points of the region of interest. The perpendicular diameter is the measurement of a straight line drawn between the widest points of the region of interest which cuts across the diameter measurement at an angle of 90°.

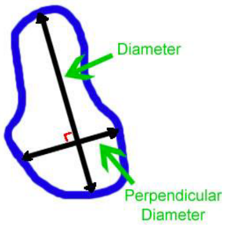

Roughness (rugosity) is equal to the absolute positive volume, plus the absolute negative volume, all divided by the surface. It is the average height of the surface variation around a surface smoothed with a smoothing factor. It is based on the smoothing surface and not on the contour. It reflects the regularity or lack thereof on the skin surface. The choice of the smoothing factor depends on the region of interest. High smoothing (more smoothed version of the skin surface) values stand for global volume changes (such as large folds in dynamic glabella) while small smoothing values correspond to very local volume changes. The roughness is unitless.

SUPPLEMENTARY TABLE S1. Kinetic Response Data for all Lesions Analyzed in the Clinical Trial of PDT and BCC

Explanation of information in each column of the table:

A — **Patient, Lesion (Location)**
The assigned ID number of the patient, the lesion number, and the location of the lesion on the body are indicated.
A maximum of 10 BCC lesions could be studied in any given patient.

B — **Graphical Summary**
Graphs of absolute tumor volume, plotted from yellow-highlighted values in the table to the right, are shown.

C — **3-D lesion data**
Shown are the values generated by 3-D photogrammetric analysis of each lesion at each study visit.
Details to note:
• For every lesion, 3 background measurements of 3D AbsVol were taken from areas of normal skin near the tumor.
• The average background value is shown at the right of each table, and also in the graphs.
• Clinical notes (long X short diameter, & whether lesions were raised or flat) were only provided for Cleveland patients.

D — **Visit numbers 1-5**
For parameters listed at the left, individual values are entered to the right for each study visit.

E — **BCC lesion clearance**
The first visit at which the 3D AbsVol was statistically similar to background is defined as the time when the tumor cleared.
Therefore, the time of tumor clearance is defined as either **V3, V4, or V5**.
Tumors still present at V5 are defined as PDT-resistant, and designed "**NC**" (not cleared).

F — **Diagnosis by Biopsy:**
Histological diagnoses of biopsied lesions were made by a Board-certified dermatopathologist.
In Cleveland, the diagnosis of BCC was determined by the presence of distinct clinical features and only those lesions that failed to clear were biopsied. In Arizona, patient's lesions were biopsied prior to enrollment to establish the BCC diagnosis.

CLEVELAND PATIENTS:

A	B	C	D					E	F
Patient, Lesion (Location)	Graphical Summary	3-D lesion data	Visit 1	Visit 2	Visit 3	Visit 4	Visit 5	BCC lesion cleared at:	Biopsy result

Pt 1, Lesion 1
(scalp)

001KB L1

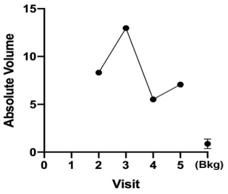

001KB L1	V1	V2	V3	V4	V5
	Visit Number				
Clinical height	Raised	Raised	raised	raised	Flat
Clinical diameters (mm)	13 x 5	20 x 9	20 x 9	16 x 8	14 x 10
3D diameter (*mm*)		18.68	21.26	17.77	16.64
3D perp diameter (*mm*)		11.51	12.52	10.86	10.50
3D av height (*mm*)	Photo	0.044	0.024	0.004	0.024
3D volume (*mm^3*)	missing	4.171	-4.269	-4.948	-1.640
3D absol volume (*mm^3*)		8.314	12.975	5.522	7.069

BCC lesion cleared at: **V5**

Biopsy at Visit 5: Prurigo nodule (no tumor)

Background, 3D Absol Vol in non-tumor areas:

			MEAN	SD
0.708	0.886	1.026	**0.873**	0.159

Pt 1, Lesion 2
(temple)

001KB L2

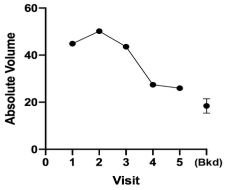

001KB L2	V1	V2	V3	V4	V5
	Visit Number				
Clinical height	raised	raised	raised	raised	Flat
Clinical diameters (mm)	15 x 11	20 x 11	20 x 11	10 x 10	10 x 10
3D diameter (*mm*)	20.03	24.16	n/d	15.79	14.60
3D perp diameter (*mm*)	17.64	17.71	n/d	15.05	12.79
3D av height (*mm*)	0.058	0.198	n/d	0.170	0.190
3D volume (*mm^3*)	-15.407	46.572	41.975	26.019	25.502
3D absol volume (*mm^3*)	44.856	50.176	43.591	27.413	25.959

BCC lesion cleared at: **V4**

Biopsy at Visit 5: Folliculitis (no tumor)

Background, 3D Absol Vol in non-tumor areas:

			MEAN	SD
20.734	14.957	19.544	**18.411**	3.050

Pt 1, Lesion 3
(forehead)

001KB L3

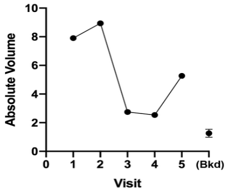

001KB L3	V1	V2	V3	V4	V5
	Visit Number				
Clinical height	Raised	Raised	Raised	Raised	Raised
Clinical diameters (mm)	18 x 18	7 x 3	7 x 6	6 x 5	6 x 6
3D diameter (*mm*)	7.56	8.08	7.80	5.91	6.32
3D perp diameter (*mm*)	6.64	6.58	6.78	4.13	4.42
3D av height (*mm*)	0.081	0.100	0.109	0.092	0.166
3D volume (*mm^3*)	2.068	0.000	2.742	2.337	5.264
3D absol volume (*mm^3*)	7.905	8.940	2.744	2.534	5.270

BCC lesion cleared at: **NC**

Biopsy at Visit 5: BCC, nodular & micronodular

Background, 3D Absol Vol in non-tumor areas:

			MEAN	SD
1.579	1.036	1.180	**1.265**	0.281

P1, Lesion 4
(back)

001KB L4

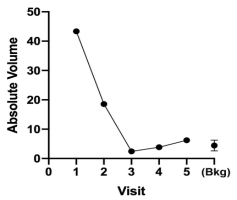

	Visit Number				
001KB L4	V1	V2	V3	V4	V5
Clinical height	Raised	Raised	Raised	Raised	invisible
Clinical diameters (mm)	11 x 11	7 x 5	7 x 5	4 x 3	asurable
3D diameter (*mm*)	8.92	6.95	12.67	12.70	12.80
3D perp diameter (*mm*)	7.32	4.58	9.06	9.05	8.96
3D av height (*mm*)	0.071	0.052	0.055	0.034	0.070
3D volume (*mm^3*)	42.885	18.217	4.580	2.940	5.900
3D absol volume (*mm^3*)	43.334	18.573	4.920	3.848	6.241

V3

Background, 3D Absol Vol in non-tumor areas:

			MEAN	SD
4.615	2.512	6.241	**4.456**	1.870

Pt 1, Lesion 5
(back)

001LB K5

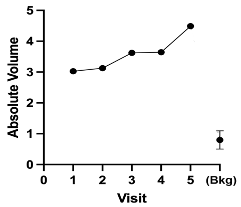

	Visit Number				
001KB L5	V1	V2	V3	V4	V5
Clinical height	Raised	Raised	Raised	Raised	Raised
Clinical diameters (mm)	6 x 6	10 x 5	10 x 5	4 x 5	5 x 4
3D diameter (*mm*)	4.95	9.28	6.18	4.39	6.47
3D perp diameter (*mm*)	4.25	6.27	4.03	4.09	4.98
3D av height (*mm*)	0.058	0.060	0.067	0.102	0.167
3D volume (*mm^3*)	2.095	2.165	3.522	3.613	4.395
3D absol volume (*mm^3*)	3.029	3.127	3.625	3.645	4.488

NC

Biopsy at Visit 5:
BCC, nodular & micronodular

Background, 3D Absol Vol in non-tumor areas:

			MEAN	SD
1.140	0.601	0.656	**0.799**	0.297

Pt 1, Lesion 6
(forearm)

001KB L6

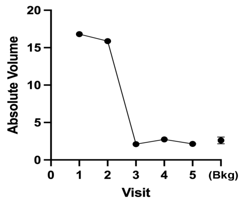

	Visit Number				
001KB L6	V1	V2	V3	V4	V5
Clinical height	Slightly R	Sl. raised	Sl. raisec	Sl. raised	Flat
Clinical diameters (mm)	6 x 6	5 x 4	5 x 4	10 x 8	10 x 8
3D diameter (*mm*)	7.79	5.45	8.27	8.85	8.44
3D perp diameter (*mm*)	7.03	4.82	7.22	7.23	6.99
3D av height (*mm*)	0.047	0.039	0.051	0.056	0.051
3D volume (*mm^3*)	16.790	15.670	2.035	2.561	2.055
3D absol volume (*mm^3*)	16.801	15.882	2.102	2.744	2.145

V3

Background, 3D Absol Vol in non-tumor areas:

			MEAN	SD
2.743	2.107	2.996	**2.615**	0.458

Patient / Lesion (Location)	Graphical Summary	3-D lesion data	Visit 1	Visit 2	Visit 3	Visit 4	Visit 5	BCC lesion cleared at:	Biopsy result

Pt 2, Lesion 1
(hairline)

002BP L1

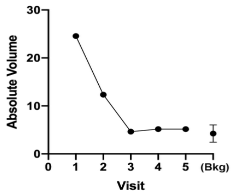

	Visit Number				
002BP L1	V1	V2	V3	V4	V5
Clinical height	Raised	n/d	Raised	Flat	Normal
Clinical diameters (mm)	15 x 9	n/d	15 x 9	15 x 9	visually
3D diameter (*mm*)	11.42	10.12	13.65	16.10	n/d
3D perp diameter (*mm*)	8.81	8.69	10.41	10.13	n/d
3D av height (*mm*)	0.392	0.017	0.022	0.150	n/d
3D volume (*mm^3*)	24.101	-12.009	0.236	4.732	n/d
3D absol volume (*mm^3*)	24.558	12.326	4.636	5.160	4.228

V3

Background, 3D Absol Vol in non-tumor areas:

			MEAN	SD
2.877	6.300	3.508	**4.228**	1.821

Pt 2, Lesion 2
(forehead)

002BP L2

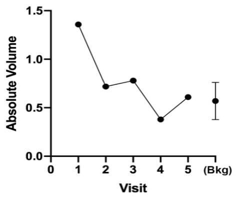

	Visit Number				
002BP L2	V1	V2	V3	V4	V5
Clinical height	Raised	n/d	Flat	Flat	'mal skin
Clinical diameters (mm)	6 x 4	n/d	4 x 4	4 x 4	asurable
3D diameter (*mm*)	5.35	4.71	Io lesion	No lesion	Io lesion
3D perp diameter (*mm*)	3.03	3.26	visible	visible	visible
3D av height (*mm*)	0.047	0.016	--	--	--
3D volume (*mm^3*)	0.43	0.04	--	--	--
3D absol volume (*mm^3*)	1.358	0.719	0.779	0.379	0.609

V4

Background, 3D Absol Vol in non-tumor areas:

			MEAN	SD
0.774	0.539	0.395	**0.569**	0.191

Pt 2, Lesion 5
(chin)

002BP L5

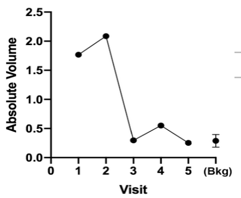

	Visit Number				
002BP L5	V1	V2	V3	V4	V5
Clinical height	Raised	n/d	Sl. raisec	No lesion	No lesion
Clinical diameters (mm)	3 x 3	n/d	3 x 3	visible	Iot visible
3D diameter (*mm*)	3.39	3.85	2.91	--	--
3D perp diameter (*mm*)	2.65	3.18	2.26	--	--
3D av height (*mm*)	0.242	0.260	0.032	--	--
3D volume (*mm^3*)	1.768	2.078	0.215	0.425	0.106
3D absol volume (*mm^3*)	1.768	2.086	0.297	0.550	0.253

V3

Background, 3D Absol Vol in non-tumor areas:

			MEAN	SD
0.413	0.243	0.208	**0.288**	0.110

Pt 2, Lesion 3: Not used because problem with 3-D reconstruction
Pt 2, Lesion 4: Not used because unclear in several photos

Patient / Lesion (Location)	Graphical Summary	3-D lesion data	Visit 1	Visit 2	Visit 3	Visit 4	Visit 5	BCC lesion cleared at:	Biopsy result

Pt 3, Lesion 1
(foot)

003MM L1

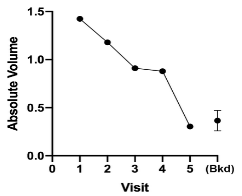

	Visit Number				
003MM L1	V1	V2	V3	V4	V5
Clinical height	Raised	Raised	Raised	Flat/gon	No lesion
Clinical diameters (mm)	5 x 5	5 x 5	5 x 5	4 x 4	visible
3D diameter (*mm*)	5.85	5.43	5.53	5.32	--
3D perp diameter (*mm*)	5.53	5.13	4.63	3.48	--
3D av height (*mm*)	0.004	0.003	0.005	0.006	--
3D volume (*mm^3*)	1.424	1.179	0.870	0.879	0.236
3D absol volume (*mm^3*)	1.424	1.179	0.910	0.879	0.304

V5

Background, 3D Absol Vol in non-tumor areas:

			MEAN	SD
0.305	0.303	0.488	**0.365**	0.106

Pt 3, Lesion 2
(leg)

003MM L2

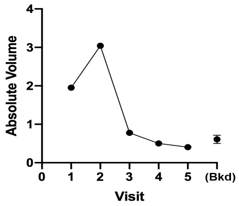

	Visit Number				
003MM L2	V1	V2	V3	V4	V5
Clinical height	Raised	Raised	Flat/Gor	Flat/Gon	Not visible
Clinical diameters (mm)	5 x 4	5 x 4	5 x 5	5 x 5	asurable
3D diameter (*mm*)	5.10	5.34	1.30	1.66	1.00
3D perp diameter (*mm*)	4.71	5.08	1.03	1.41	0.59
3D av height (*mm*)	0.143	0.134	0.014	0.028	0.019
3D volume (*mm^3*)	1.950	3.042	0.765	0.500	0.404
3D absol volume (*mm^3*)	1.950	3.042	0.777	0.500	0.405

V4

Background, 3D Absol Vol in non-tumor areas:

			MEAN	SD
0.500	0.606	0.715	**0.607**	0.108

Pt 3, Lesion 3
(ankle)

003MM L3

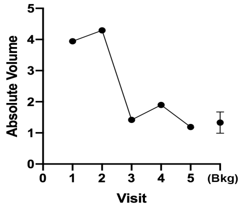

	Visit Number				
003MM L3	V1	V2	V3	V4	V5
Clinical height	Raised	Raised	Flat	Slightly r	Flat
Clinical diameters (mm)	7 x 7	7 x 7	6 x 5	4 x 4	4 x 4
3D diameter (*mm*)	5.83	8.76	4.86	5.01	4.19
3D perp diameter (*mm*)	4.46	6.08	3.67	4.55	3.79
3D av height (*mm*)	0.040	0.063	0.033	0.043	0.017
3D volume (*mm^3*)	3.923	4.210	1.100	1.647	0.891
3D absol volume (*mm^3*)	3.943	4.294	1.419	1.896	1.190

V5

Background, 3D Absol Vol in non-tumor areas:

			MEAN	SD
1.421	1.622	0.953	**1.332**	0.344

Pt 3, Lesion 4
(leg)

003MM L4

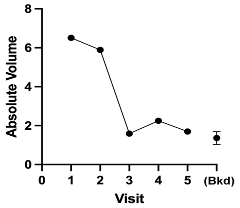

	Visit Number				
003MM L4	V1	V2	V3	V4	V5
Clinical height	Raised	Raised	Flat	No lesion	No lesion
Clinical diameters (mm)	10 x 5	10 x 5	7 x 3	visible	visible
3D diameter (*mm*)	10.16	10.78	3.22	--	--
3D perp diameter (*mm*)	5.83	6.57	1.84	--	--
3D av height (*mm*)	0.005	0.006	0.011	--	--
3D volume (*mm^3*)	6.474	5.754	1.229	2.172	-0.079
3D absol volume (*mm^3*)	6.510	5.890	1.593	2.249	1.699

V5

Background, 3D Absol Vol in non-tumor areas:

			MEAN	SD
1.073	1.437	1.589	**1.366**	0.265

Pt 3, Lesion 5
(arm)

003MM L5

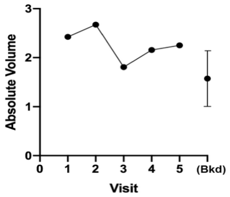

	Visit Number				
003MM L5	V1	V2	V3	V4	V5
Clinical height	Raised	Raised	Flat	Flat	Flat
Clinical diameters (mm)	10 x 6	10 x 6	7 x 6	n/d	4 x 3
3D diameter (*mm*)	9.47	10.14	4.33	4.11	4.24
3D perp diameter (*mm*)	6.24	7.63	2.54	2.45	2.09
3D av height (*mm*)	0.053	0.061	0.024	0.008	0.031
3D volume (*mm^3*)	2.271	2.462	1.277	1.971	1.762
3D absol volume (*mm^3*)	2.423	2.674	1.806	2.155	2.252

V5

Biopsy at Visit 5:
Hyperkeratosis & chronic inflammation (no tumor)

			MEAN	SD
1.337	1.160	2.221	**1.573**	0.568

Pt 3, Lesion 6
(back)

003MM L6

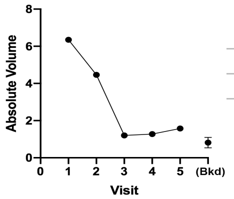

	Visit Number				
003MM L6	V1	V2	V3	V4	V5
Clinical height	Raised	Raised	Slightly r	Flat	Flat
Clinical diameters (mm)	9 x 4	9 x 4	7 x 4	6 x 5	7 x 4
3D diameter (*mm*)	7.67	8.16	7.45	7.32	2.73
3D perp diameter (*mm*)	6.03	5.47	3.90	4.97	1.02
3D av height (*mm*)	0.200	0.147	0.041	0.073	0.015
3D volume (*mm^3*)	6.335	4.377	1.084	0.147	-0.200
3D absol volume (*mm^3*)	6.352	4.466	1.204	1.276	1.578

V3

Background, 3D Absol Vol in non-tumor areas:

			MEAN	SD
0.520	1.077	0.845	**0.814**	0.280

Patient / Lesion (Location)	Graphical Summary	3-D lesion data	Visit 1	Visit 2	Visit 3	Visit 4	Visit 5	BCC lesion cleared at:	Biopsy result

Pt 4, Lesion 1
(knee)

In retropsect for patient 4, lesions #1-5 were atrophic shave bipsy scars; lesion #6 was an SCC.

(Did not graph)

004JB L1	V1	V2	V3	V4	V5
	Visit Number				
Clinical height	18x11	18x11	15x10	12x9	9x12
Clinical diameters (mm)	scaly shin	scaly shin	Flat	Flat	Flat
3D diameter (*mm*)	17.119	17.075	16.051	13.021	10.797
3D perp diameter (*mm*)	12.963	12.134	12.118	9.875	9.283
3D av height (*mm*)	0.004	0.013	0.003	0.004	0.001
3D volume (*mm^3*)	0.635	1.95	0.423	0.396	0.05
3D absol volume (*mm^3*)	18.735	17.361	17.119	8.996	8.244

Background, 3D Absol Vol in non-tumor areas:

			MEAN	SD
4.027	5.764	6.245	**5.345**	1.167

Pt 4, Lesion 2
(back)

In retropsect for patient 4, lesions #1-5 were atrophic shave bipsy scars; lesion #6 was an SCC.

(Did not graph)

004JB L2	V1	V2	V3	V4	V5
	Visit Number				
Clinical height	5x5	5x5	9x6	3x6	3x6
Clinical diameters (mm)			Raised	Flat	Looks normal
3D diameter (*mm*)	6.486	7.876	5.92	6.539	6.518
3D perp diameter (*mm*)	5.546	6.861	5.081	5.624	5.612
3D av height (*mm*)	0.029	0.008	0.009	0.016	0.009
3D volume (*mm^3*)	0.656	0.282	0.165	0.368	0.217
3D absol volume (*mm^3*)	0.776	0.876	0.878	0.438	0.406

Background, 3D Absol Vol in non-tumor areas:

			MEAN	SD
0.275	0.317	0.516	**0.369**	0.129

Pt 4, Lesion 3
(back)

Atrophic BCC (negative volumes)
Lesion became more fibrotic-looking after each PDT Rx.

(Did not graph)

004JB L3	V1	V2	V3	V4	V5
	Visit Number				
Clinical height	10x6	8x9	9x8	8x10	8x10
Clinical diameters (mm)				Depresse	Depressed
3D diameter (*mm*)	12.592	10.352	9.247	9.904	9.75
3D perp diameter (*mm*)	8.302	7.865	7.145	7.062	7.16
3D av height (*mm*)	0	0	0	0	0.001
3D volume (*mm^3*)	0	0.014	0.007	0.008	0.031
3D absol volume (*mm^3*)	20.665	15.361	18.422	13.218	12.183

Background, 3D Absol Vol in non-tumor areas:

			MEAN	SD
1.348	1.98	1.559	**1.629**	0.322

Pt 4, Lesion 4
(back)

Old sunken biopsy scar;
(Shiny surface in a smooth depression in the skin. The 3D quantif'n shows negative vol)

(Did not graph)

004JB L4	V1	V2	V3	V4	V5
	Visit Number				
Clinical height	10x9	10x9	10x9	10x9	10x9
Clinical diameters (mm)					Depressed
3D diameter (*mm*)	12.395	11.2	10.691	11.608	9.724
3D perp diameter (*mm*)	9.87	8.591	8.183	8.436	7.603
3D av height (*mm*)	0	0	0	0	0
3D volume (*mm^3*)	0.014	0	0	0.002	0
3D absol volume (*mm^3*)	21.502	18.635	20.919	19.452	15.163

Background, 3D Absol Vol in non-tumor areas:

			MEAN	SD
2.575	1.222	2.042	**1.946**	0.682

Pt 4, Lesion 5
(back)

Sunken nature of depressed lesion only became apparent upon magnification with the 3D images, and neg volumes

(Did not graph)

004JB L5	V1	V2	V3	V4	V5
	Visit Number				
Clinical height	6x6	5x7	5x10	5x10	5x10
Clinical diameters (mm)					Depressed
3D diameter (*mm*)	9.772	10.064	10.151	11.058	9.667
3D perp diameter (*mm*)	8.895	6.977	6.335	7.191	5.798
3D av height (*mm*)	0.001	0.001	0	0	0
3D volume (*mm^3*)	0.027	0.03	0.001	0.025	0
3D absol volume (*mm^3*)	8.231	11.035	15.937	11.669	7.604

Background, 3D Absol Vol in non-tumor areas:

			MEAN	SD
0.893	1.014	0.713	**0.873**	0.151

Pt 4, Lesion 6
(back)

(Not a BCC, diid not graph)

004JB L6	V1	V2	V3	V4	V5
	Visit Number				
Clinical height			Not visit	Flat	Flat
Clinical diameters (mm)	4x4	4x3	4x3	2x2	2x2
3D diameter (*mm*)					
3D perp diameter (*mm*)					
3D av height (*mm*)					
3D volume (*mm^3*)	0.9995	0.7911	0.2171	0.2441	0.2893
3D absol volume (*mm^3*)	0.9995	0.7911	0.23	0.259	0.2896

Biopsy at Visit 5:
Squamous cell CA

Background, 3D Absol Vol in non-tumor areas:

MEAN	SD

Patient / Lesion (Location)	Graphical Summary	3-D lesion data	Visit 1	Visit 2	Visit 3	Visit 4	Visit 5	BCC lesion cleared at:	Biopsy result

Pt 5, Lesion 1
(breast)

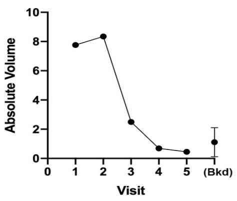

005CF L1	V1	V2	V3	V4	V5
	Visit Number				
Clinical height			Flat	Flat	No lesion
Clinical diameters (mm)	13 x 12	13 x 12	8 x 8	5 x 4	visible
3D diameter (*mm*)	13.43	13.94	5.47	5.95	--
3D perp diameter (*mm*)	11.99	10.85	4.83	4.65	--
3D av height (*mm*)	0.067	0.072	0.013	0.006	--
3D volume (*mm^3*)	6.347	7.977	0.957	-0.219	0.034
3D absol volume (*mm^3*)	7.764	8.348	2.496	0.685	0.456

BCC lesion cleared at: **V4**

Background, 3D Absol Vol in non-tumor areas:

			MEAN	SD
1.462	1.182	0.691	**1.112**	0.995

Pt 5, Lesion 2
(arm)

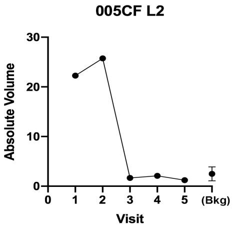

005CF L2	V1	V2	V3	V4	V5
	Visit Number				
Clinical height			Flat	Flat	Flat
Clinical diameters (mm)	9 x 7	9 x 7	5 x 4	5 x 5	5 x 5
3D diameter (*mm*)	8.94	8.00	7.58	7.36	7.36
3D perp diameter (*mm*)	8.33	6.76	6.49	5.56	5.56
3D av height (*mm*)	0.012	0.030	0.052	0.036	0.004
3D volume (*mm^3*)	21.768	25.700	1.592	2.083	1.002
3D absol volume (*mm^3*)	22.260	25.737	1.663	2.095	1.214

V3

Background, 3D Absol Vol in non-tumor areas:

			MEAN	SD
4.118	1.596	1.761	**2.492**	1.411

Pt 5, Lesion 3
(arm)

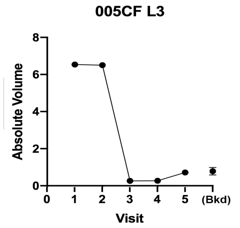

005CF L3	V1	V2	V3	V4	V5
	Visit Number				
Clinical height			Flat	No lesion	No lesion
Clinical diameters (mm)	9 x 6	9 x 6	4 x 5	visible	visible
3D diameter (*mm*)	8.31	7.71	8.27	--	--
3D perp diameter (*mm*)	7.64	6.87	5.42	--	--
3D av height (*mm*)	0.022	0.016	0.004	--	--
3D volume (*mm^3*)	6.363	6.322	-0.096	-0.092	0.469
3D absol volume (*mm^3*)	6.535	6.505	0.264	0.272	0.716

V3

Background, 3D Absol Vol in non-tumor areas:

			MEAN	SD
0.746	0.593	1.001	**0.780**	0.206

Pt 5, Lesion 4
(back)

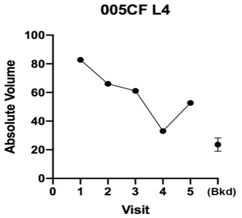

005CF L4	V1	V2	V3	V4	V5
	Visit Number				
Clinical height				Mostly fl	Mostly flat
Clinical diameters (mm)	39x15	39x15	39x22	35x20	35x20
3D diameter (*mm*)	37.58	36.48	39.08	41.24	40.29
3D perp diameter (*mm*)	25.08	24.46	21.36	23.22	19.07
3D av height (*mm*)	0.060	0.071	0.078	0.090	0.144
3D volume (*mm^3*)	80.191	57.438	34.189	23.461	40.919
3D absol volume (*mm^3*)	82.831	66.033	61.138	33.065	52.698

NC

Biopsy at Visit 5:
BCC, nodular

Background, 3D Absol Vol in non-tumor areas:

			MEAN	SD
25.747	18.293	26.955	**23.665**	4.692

Pt 5, Lesion 5
(back)

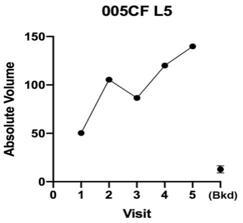

005CF L5	V1	V2	V3	V4	V5
	Visit Number				
Clinical height	with	with 6x6	with	with	with
Clinical diameters (mm)	15 x 12	15 x 12	17 x 10	15 x 12	15 x 12
3D diameter (*mm*)	13.31	17.62	20.32	20.62	20.14
3D perp diameter (*mm*)	12.10	15.91	12.47	14.27	14.86
3D av height (*mm*)	0.079	0.272	0.308	0.483	0.318
3D volume (*mm^3*)	-5.863	4.369	29.653	99.505	89.709
3D absol volume (*mm^3*)	50.306	105.462	86.617	120.030	#######

NC

Biopsy at Visit 5:
BCC, infiltrative

Background, 3D Absol Vol in non-tumor areas:

			MEAN	SD
16.809	9.940	11.666	**12.805**	3.573

Pt 5, Lesion 6
(back)

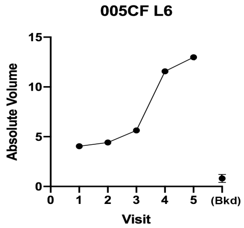

005CF L6	V1	V2	V3	V4	V5
	Visit Number				
Clinical height	Raised	Raised	Raised	Raised	Raised
Clinical diameters (mm)	9 x 5	9 x 5	8 x 5	8 x 5	10 x 5
3D diameter (*mm*)	8.01	8.42	8.08	8.90	9.09
3D perp diameter (*mm*)	4.98	5.09	4.78	5.22	6.58
3D av height (*mm*)	0.124	0.142	0.205	0.348	0.315
3D volume (*mm^3*)	3.485	4.233	5.491	11.366	12.573
3D absol volume (*mm^3*)	4.038	4.421	5.631	11.567	12.980

NC

Biopsy at Visit 5:
BCC, nodular and infiltrative

Background, 3D Absol Vol in non-tumor areas:

			MEAN	SD
0.989	0.709	0.713	**0.803**	0.161

Pt 5, Lesion 7
(back)

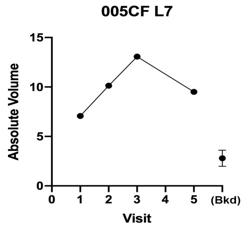

005CF L7	V1	V2	V3	V4	V5
	Visit Number				
Clinical height	Raised	Raised	Raised	Raised	Raised
Clinical diameters (mm)	12 x 10	12 x 10	10 x 9	12 x 6	12 x 10
3D diameter (*mm*)	11.75	12.81	13.59	n/d	12.40
3D perp diameter (*mm*)	8.21	7.03	7.11	n/d	10.84
3D av height (*mm*)	0.060	0.166	0.238	n/d	0.067
3D volume (*mm^3*)	1.021	7.662	12.702	n/d	0.815
3D absol volume (*mm^3*)	7.069	10.128	13.066	n/d	9.510

NC

Biopsy at Visit 5:
BCC, nodular & and adenoid

Background, 3D Absol Vol in non-tumor areas:

			MEAN	SD
2.409	2.239	3.733	**2.794**	0.818

Pt 5, Lesion 8
(neck)

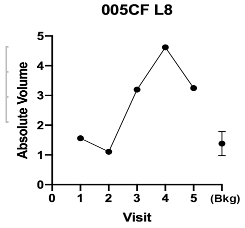

005CF L8	V1	V2	V3	V4	V5
	Visit Number				
Clinical height	Raised	Raised	Raised	Raised	Raised
Clinical diameters (mm)	5 x 5	5 x 5	7 x 6	7 x 5	6 x 5
3D diameter (*mm*)	7.24	6.56	7.88	8.23	8.21
3D perp diameter (*mm*)	5.83	4.72	5.29	5.88	5.27
3D av height (*mm*)	0.045	0.026	0.080	0.114	0.085
3D volume (*mm^3*)	0.941	-0.065	1.341	3.032	2.134
3D absol volume (*mm^3*)	1.561	1.106	3.199	4.618	3.246

NC

Biopsy at Visit 5:
BCC, micronodular

Background, 3D Absol Vol in non-tumor areas:

			MEAN	SD
1.824	1.028	1.296	**1.382**	0.405

Pt 5, Lesion 9
(arm)

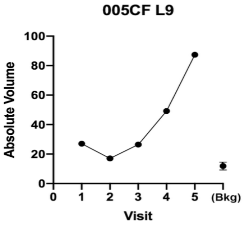

005CF L9	Visit Number V1	V2	V3	V4	V5
Clinical height	Raised	Raised	Raised	Raised	Raised
Clinical diameters (mm)	18 x 8	18 x 8	20 x 10	20 x 15	20 x 15
3D diameter (*mm*)	20.68	20.10	21.39	23.10	23.47
3D perp diameter (*mm*)	10.96	10.48	13.28	14.88	15.76
3D av height (*mm*)	0.164	0.101	0.142	0.220	0.343
3D volume (*mm^3*)	27.052	15.551	26.381	49.177	87.351
3D absol volume (*mm^3*)	27.065	17.004	26.444	49.244	87.405

NC

Biopsy at Visit 5:
BCC micronodular and infiltrative

Background, 3D Absol Vol in non-tumor areas:

			MEAN	SD
9.873	12.824	12.967	**11.888**	1.747

Pt 5, Lesion 10
(arm)

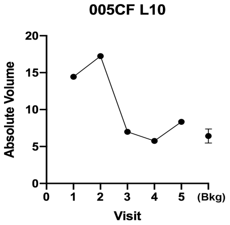

005CF L10	Visit Number V1	V2	V3	V4	V5
Clinical height	Sl. Raised	Sl. Raised	Sl. Raise	Flat	Slightly raised
Clinical diameters (mm)	10 x 6	10 x 6	6 x 6	5 x 5	5 x 5
3D diameter (*mm*)	9.91	9.08	--	--	--
3D perp diameter (*mm*)	7.22	7.11	--	--	--
3D av height (*mm*)	0.076	0.026	--	--	--
3D volume (*mm^3*)	14.374	17.243	6.942	5.660	8.322
3D absol volume (*mm^3*)	14.443	17.244	6.993	5.751	8.341

V3

Biopsy at Visit 5:
Dermal fibrosis, solar elastosis (no tumor)

Background, 3D Absol Vol in non-tumor areas:

			MEAN	SD
6.133	7.497	5.666	**6.432**	0.951

Patient / Lesion (Location)	Graphical Summary	3-D lesion data	Visit 1	Visit 2	Visit 3	Visit 4	Visit 5	BCC lesion cleared at:	Biopsy result

Pt 6, Lesion 1
(back)

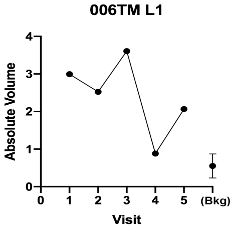

006TM L1	Visit Number V1	V2	V3	V4	V5
Clinical height	Raised	Raised w/	Raised	Flat, cent	Flat, central papule
Clinical diameters (mm)	12x17	12x17	12x17	2x5	3x5
3D diameter (*mm*)	4.63	5.26	7.02	5.68	6.82
3D perp diameter (*mm*)	2.34	4.48	3.19	2.67	3.37
3D av height (*mm*)	0.067	0.102	0.220	0.090	0.140
3D volume (*mm^3*)	2.395	-0.084	3.607	0.847	2.031
3D absol volume (*mm^3*)	2.996	2.526	3.609	0.882	2.066

NC

Biopsy at Visit 5:
BCC, nodular, microdular, & adenoid

Background, 3D Absol Vol in non-tumor areas:

			MEAN	SD
0.486	0.901	0.271	**0.553**	0.320

Pt 6, Lesion 2
(back)

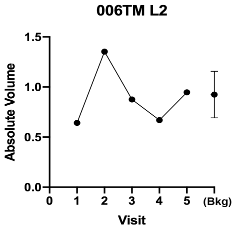

006TM L2	Visit Number V1	V2	V3	V4	V5
Clinical height	Raised	Raised	Flat	Flat	Flat
Clinical diameters (mm)	18x15	18x15	18x15	10x10	10x10
3D diameter (*mm*)	18.24	17.63	16.42	--	--
3D perp diameter (*mm*)	12.97	12.18	12.54	--	--
3D av height (*mm*)	0.010	0.001	0.004	--	--
3D volume (*mm^3*)	0.185	-1.349	0.722	-0.165	-0.014
3D absol volume (*mm^3*)	0.642	1.353	0.875	0.669	0.947

V3

Background, 3D Absol Vol in non-tumor areas:

			MEAN	SD
1.103	0.662	1.008	**0.925**	0.232

Pt 6, Lesion 3
(back)

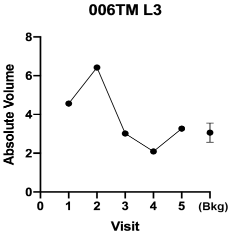

006TM L3	Visit Number V1	V2	V3	V4	V5
Clinical height	Raised	Raised	Flat	Flat	Flat
Clinical diameters (mm)	10x10	10x10	10x10	6x9	6x9
3D diameter (*mm*)	11.32	11.76	2.39	2.80	1.36
3D perp diameter (*mm*)	7.46	8.66	2.09	2.14	1.05
3D av height (*mm*)	0.067	0.088	0.020	0.051	0.042
3D volume (*mm^3*)	4.144	5.283	1.592	0.959	-1.616
3D absol volume (*mm^3*)	4.562	6.422	3.018	2.092	3.267

V3

Background, 3D Absol Vol in non-tumor areas:

			MEAN	SD
2.935	2.645	3.607	**3.062**	0.494

Pt 6, Lesion 4
(back)

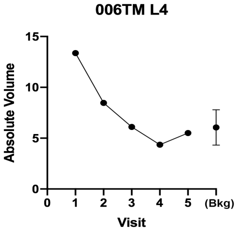

006TM L4	Visit Number V1	V2	V3	V4	V5
Clinical height	Raised	Raised	Flat	Flat	Flat
Clinical diameters (mm)	10x22	10x22	10x22	9x15	9x15
3D diameter (*mm*)	20.77	20.34	18.09	--	--
3D perp diameter (*mm*)	11.60	11.61	10.91	--	--
3D av height (*mm*)	0.051	0.048	0.011	--	--
3D volume (*mm^3*)	4.183	6.463	-3.085	-2.390	-3.263
3D absol volume (*mm^3*)	13.372	8.463	6.109	4.361	5.509

V3

Background, 3D Absol Vol in non-tumor areas:

			MEAN	SD
4.146	6.458	7.555	**6.053**	1.740

Visit Number

Pt 6, Lesion 5
(back)

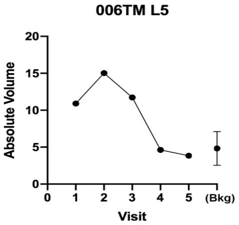

006TM L5	V1	V2	V3	V4	V5
Clinical height	Raised	Raised	Flat	Flat	Flat
Clinical diameters (mm)	10x15	10x15	10x10	10x12	10x12
3D diameter (*mm*)	17.89	17.45	15.65	--	--
3D perp diameter (*mm*)	13.12	11.94	10.16	--	--
3D av height (*mm*)	0.009	0.003	0.002	--	--
3D volume (*mm^3*)	-8.281	-13.947	-11.154	-2.229	0.200
3D absol volume (*mm^3*)	10.902	15.025	11.733	4.622	3.837

V4

Background, 3D Absol Vol in non-tumor areas:

			MEAN	SD
6.262	6.020	2.200	**4.827**	2.279

Patient / Lesion (Location)	Graphical Summary	3-D lesion data	Visit 1	Visit 2	Visit 3	Visit 4	Visit 5	BCC lesion cleared at:	Biopsy result

Pt 7, Lesion 1
(arm)

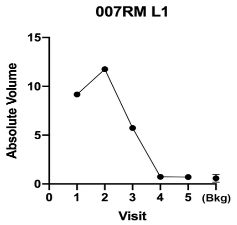

	Visit Number				
007RM L1	V1	V2	V3	V4	V5
Clinical height	Raised	Raised	Sl. Raise	Sl. Raised	Flat
Clinical diameters (mm)	8x10	8x10	5x10	8x4	2x2
3D diameter (*mm*)	12.95	11.42	7.74	7.91	5.88
3D perp diameter (*mm*)	10.07	7.41	3.51	4.56	4.17
3D av height (*mm*)	0.063	0.066	0.027	0.010	0.011
3D volume (*mm^3*)	9.110	11.703	5.639	0.233	-0.247
3D absol volume (*mm^3*)	9.169	11.761	5.734	0.728	0.700

V4

Biopsy at Visit 5:
Bx: dermal scar
chronic folliclitis
(no tumor)

Background, 3D Absol Vol in non-tumor areas:

			MEAN	SD
0.439	0.590	0.734	**0.587**	0.147

Pt 7, Lesion 2
(chest)

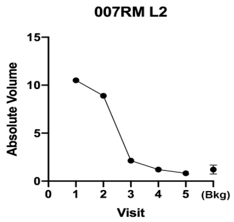

	Visit Number				
007RM L2	V1	V2	V3	V4	V5
Clinical height	Two dom	Two dom	Two pap	Flat, ill-d	Flat, ill-defined
Clinical diameters (mm)	5x11	5x11	5x4	N/A	Not visible
3D diameter (*mm*)	4.34	4.90	--	--	--
3D perp diameter (*mm*)	3.60	3.93	--	--	--
3D av height (*mm*)	0.278	0.261	--	--	--
3D volume (*mm^3*)	10.241	8.117	1.552	-0.862	0.157
3D absol volume (*mm^3*)	10.512	8.897	2.127	1.202	0.814

V4

Background, 3D Absol Vol in non-tumor areas:

			MEAN	SD
1.743	0.912	0.964	**1.207**	0.465

Pt 7, Lesion 3
(chest)

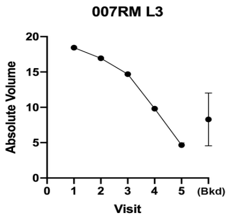

	Visit Number				
007RM L3	V1	V2	V3	V4	V5
Clinical height	Raised	Raised	Depresse	Flat	Flat
Clinical diameters (mm)	10x13	10x13	12x13	15x10	15x10
3D diameter (*mm*)	14.19	13.70	12.58	11.83	13.65
3D perp diameter (*mm*)	13.42	12.96	9.99	9.40	11.11
3D av height (*mm*)	0.126	0.079	0.082	0.076	0.042
3D volume (*mm^3*)	13.017	10.637	4.673	0.422	3.171
3D absol volume (*mm^3*)	18.445	16.947	14.689	9.810	4.654

V4

Background, 3D Absol Vol in non-tumor areas:

			MEAN	SD
9.230	11.466	4.186	**8.294**	3.729

Pt 7, Lesion 4
(leg)

Not a BCC; did not graph

	Visit Number				
007RM L4	V1	V2	V3	V4	V5
Diameter (exam)	5x12	5x12	5x12	Not meas	Not measurable
Height (exam)	Raised	Raised	Flat	Nothing \	Nothing visible
3D volume	6.5017	4.0638	-0.054		
3D absol volume	7.4166	4.8476	0.2063		

Biopsy (prior to V1
Biopsy: SCC

Patient / Lesion (Location)	Graphical Summary	3-D lesion data	Visit 1	Visit 2	Visit 3	Visit 4	Visit 5	BCC lesion cleared at:	Biopsy result

Pt 8, Lesion 3
8 / 3 (forearm)

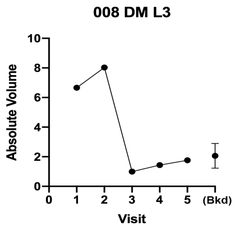

	Visit Number				
008DM L3	V1	V2	V3	V4	V5
Clinical height					
Clinical diameters (mm)	5x5	5x5	3x3	3x3	3x3
3D diameter (*mm*)	5.38	7.55	3.05	--	--
3D perp diameter (*mm*)	4.89	6.34	2.33	--	--
3D av height (*mm*)	0.185	0.219	0.011	--	--
3D volume (*mm^3*)	6.280	7.369	-0.098	1.350	1.725
3D absol volume (*mm^3*)	6.660	8.027	0.996	1.441	1.759

V3

Background, 3D Absol Vol in non-tumor areas:

			MEAN	SD
2.819	2.209	1.169	**2.066**	0.834

Pt 8, Lesion 4
8 / 4 (chest)

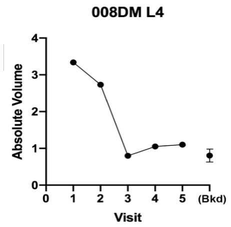

	Visit Number				
008DM L4	V1	V2	V3	V4	V5
Clinical height	raised	raised	flat	flat	flat
Clinical diameters (mm)	9x5	8x8	2x2	1x1	1x1
3D diameter (*mm*)	6.89	7.86			
3D perp diameter (*mm*)	4.35	6.78			
3D av height (*mm*)	0.048	0.047			
3D volume (*mm^3*)	-1.388	0.406	0.277	-0.198	0.865
3D absol volume (*mm^3*)	3.340	2.731	0.797	1.050	1.102

V3

Background, 3D Absol Vol in non-tumor areas:

			MEAN	SD
0.601	0.907	0.907	**0.805**	0.177

Pt 8, Lesion 5
8 / 5 (back)

008DM L5

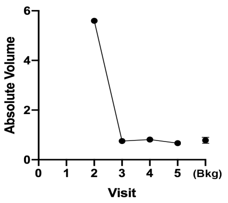

008DM L5	Visit Number V1	V2	V3	V4	V5
Clinical height	flat	raised	flat	flat	flat
Clinical diameters (mm)	10x5	15x5	3x8	4x7	4x5
3D diameter (*mm*)		15.38	6.22	2.44	3.45
3D perp diameter (*mm*)	No	5.99	4.60	2.24	3.09
3D av height (*mm*)	photo	0.042	0.017	0.023	0.005
3D volume (*mm^3*)		-1.477	0.082	0.352	0.084
3D absol volume (*mm^3*)		5.597	0.751	0.813	0.667

V3

Biopsy at Visit 5: Pityrosporum folliculitis (no tumor)

Background, 3D Absol Vol in non-tumor areas:

			MEAN	SD
0.907	0.776	0.667	**0.783**	0.120

Pt 8, Lesion 6
8 / 6 (back)

008DM L6

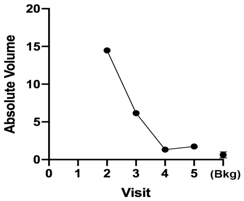

008DM L6	Visit Number V1	V2	V3	V4	V5
Clinical height		flat	raised	raised	flat
Clinical diameters (mm)		10x4	10x4	10x4	10x4
3D diameter (*mm*)		9.43	6.92	3.05	4.03
3D perp diameter (*mm*)	No	5.47	2.59	2.17	2.59
3D av height (*mm*)	photo	0.028	0.132	0.133	0.138
3D volume (*mm^3*)		-11.977	-3.538	1.125	1.205
3D absol volume (*mm^3*)		14.468	6.153	1.322	1.738

NC

Biopsy at Visit 5: BCC, nodular

Background, 3D Absol Vol in non-tumor areas:

			MEAN	SD
0.606	0.669	0.614	**0.630**	0.034

Pt 8, Lesion 1: Not used because lesion already gone at V2
Pt 8, Lesion 2: Not used because lesion already gone at V2

Patient / Lesion (Location)	Graphical Summary	3-D lesion data	Visit 1	Visit 2	Visit 3	Visit 4	Visit 5	BCC lesion cleared at:	Biopsy result

Pt 9, Lesion 1
(scalp)

009JG L1

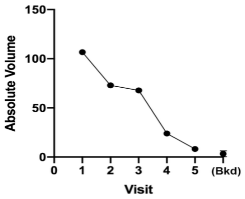

009JG L1	Visit Number V1	V2	V3	V4	V5
Clinical height	raised	raised	raised	raised	raised
Clinical diameters (mm)	12x8	12x8	15x9	13x7	5x4
3D diameter (*mm*)	13.98	12.83	13.53	12.73	7.83
3D perp diameter (*mm*)	10.48	9.43	9.40	8.52	5.43
3D av height (*mm*)	0.955	0.615	0.743	0.292	0.282
3D volume (*mm^3*)	105.484	72.749	67.820	23.957	7.792
3D absol volume (*mm^3*)	106.636	72.914	67.844	23.992	8.211

NC

Biopsy at Visit 5: BCC, nodular

Background, 3D Absol Vol in non-tumor areas:

			MEAN	SD
3.301	5.335	1.428	**3.355**	1.954

Pt 9, Lesion 2:
(scalp)

009JG L2

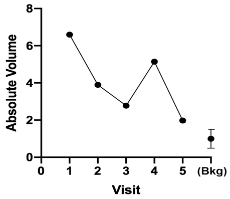

009JG L2	Visit Number V1	V2	V3	V4	V5
Clinical height	raised	raised	raised; sı	raised	flat ± papules
Clinical diameters (mm)	11x9	11x9	/papules	ı/papules	7x8
3D diameter (*mm*)	7.36	8.39	9.55	9.47	7.99
3D perp diameter (*mm*)	6.22	7.19	7.63	4.98	7.57
3D av height (*mm*)	0.105	0.064	0.032	0.100	0.102
3D volume (*mm^3*)	6.450	3.807	1.299	5.036	1.884
3D absol volume (*mm^3*)	6.599	3.897	2.777	5.142	1.977

NC

Biopsy at Visit 5: BCC, nodular and micronodular

Background, 3D Absol Vol in non-tumor areas:

			MEAN	SD
0.862	0.569	1.560	**0.997**	0.509

Pt 9, Lesion 3
(neck)

009JG L3

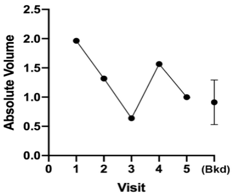

009JG L3	Visit Number V1	V2	V3	V4	V5
Clinical height	raised	raised	flat	flat	normal skin
Clinical diameters (mm)	4x5	4x5	2x2	1x1	2x2
3D diameter (*mm*)	9.36	6.39	--	--	--
3D perp diameter (*mm*)	5.20	4.89	--	--	--
3D av height (*mm*)	0.021	0.022	--	--	--
3D volume (*mm^3*)	-0.520	-0.440	-0.494	-1.161	-0.803
3D absol volume (*mm^3*)	1.964	1.317	0.640	1.566	0.998

V3

Background, 3D Absol Vol in non-tumor areas:

			MEAN	SD
0.919	1.289	0.525	**0.911**	0.382

Pt 9, Lesion 4
(neck)

009JG L4

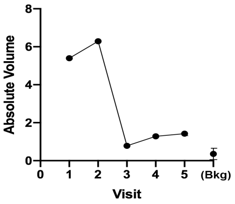

009JG L4	Visit Number V1	V2	V3	V4	V5
Clinical height	raised	slightly ra	slightly r	slightly ra	slightly raised
Clinical diameters (mm)	5x5	5x5	2x2	2x2	2x2
3D diameter (*mm*)	7.22	7.65	6.74	4.60	4.36
3D perp diameter (*mm*)	4.52	4.12	3.91	2.97	2.99
3D av height (*mm*)	0.240	0.314	0.029	0.135	0.168
3D volume (*mm^3*)	5.340	6.277	0.163	1.123	1.392
3D absol volume (*mm^3*)	5.398	6.287	0.783	1.286	1.422

NC

Biopsy at Visit 5: BCC, nodular and micronodular

Background, 3D Absol Vol in non-tumor areas:

			MEAN	SD
0.345	0.445	0.270	**0.353**	0.088

Pt 9, Lesion 5
(cheek)

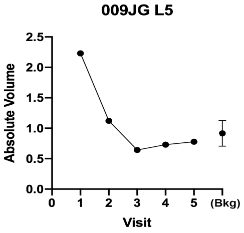

009JG L5	V1	V2	V3	V4	V5
	Visit Number				
Clinical height	raised	raised, rai	none, rai	none, no	none, none
Clinical diameters (mm)	3x3	3x3	1x2	No lesion	lo lesion
3D diameter (*mm*)	7.29	4.13	--	--	--
3D perp diameter (*mm*)	6.07	3.25	--	--	--
3D av height (*mm*)	0.101	0.047	--	--	--
3D volume (*mm^3*)	2.212	1.111	0.418	0.263	0.606
3D absol volume (*mm^3*)	2.231	1.123	0.642	0.731	0.778

V3

Background, 3D Absol Vol in non-tumor areas:

			MEAN	SD
1.009	0.674	1.064	**0.916**	0.211

Pt 9, Lesion 6
(back)

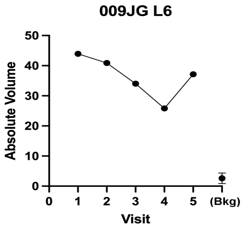

009JG L6	V1	V2	V3	V4	V5
	Visit Number				
Clinical height	soft raise	soft raise	soft raise	soft raise	soft raised
Clinical diameters (mm)	10x10	10x10	7x11	7x11	9x12
3D diameter (*mm*)	11.52	10.62	10.96	10.83	11.79
3D perp diameter (*mm*)	9.74	9.62	9.20	8.93	9.68
3D av height (*mm*)	0.570	0.608	0.482	0.379	0.473
3D volume (*mm^3*)	43.835	40.904	34.001	25.800	37.081
3D absol volume (*mm^3*)	43.923	40.905	34.001	25.813	37.130

NC

Biopsy at Visit 5:
BCC, micronodular

Background, 3D Absol Vol in non-tumor areas:

			MEAN	SD
2.458	2.521	2.845	**2.608**	0.208

Pt 9, Lesion 7
(chest)

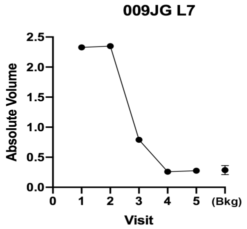

009JG L7	V1	V2	V3	V4	V5
	Visit Number				
Clinical height	raised	raised	raised	flat	flat
Clinical diameters (mm)	4x5	4x5	3x3	3x3	2x2
3D diameter (*mm*)	4.75	4.48	5.61	3.12	4.26
3D perp diameter (*mm*)	4.48	4.32	3.36	2.87	3.40
3D av height (*mm*)	0.177	0.185	0.063	0.009	0.015
3D volume (*mm^3*)	2.307	2.339	0.761	0.132	0.223
3D absol volume (*mm^3*)	2.327	2.348	0.790	0.259	0.276

V4

Biopsy at Visit 5:
Benign lichenoid keratosis (no tumor)

Background, 3D Absol Vol in non-tumor areas:

			MEAN	SD
0.337	0.265	0.259	**0.287**	0.043

Pt 9, Lesion 8
(chest)

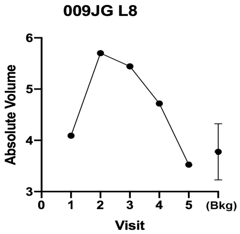

009JG L8	V1	V2	V3	V4	V5
	Visit Number				
Clinical height	slight rai	scaly raise	nearly fl	nearly fla	nearly flat
Clinical diameters (mm)	18x15	18x15	18x10	14x15	invisible
3D diameter (*mm*)	17.827	18.051	16.520	17.817	19.095
3D perp diameter (*mm*)	11.986	12.521	13.289	12.219	13.467
3D av height (*mm*)	0.025	0.021	0.014	0.015	0.019
3D volume (*mm^3*)	2.298	-0.935	-2.441	-1.107	0.533
3D absol volume (*mm^3*)	4.091	5.701	5.443	4.718	3.526

V5

Background, 3D Absol Vol in non-tumor areas:

			MEAN	SD
3.430	3.490	4.409	**3.777**	0.549

Pt 9, Lesion 9
(abdomen)

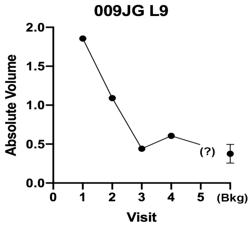

009JG L9	V1	V2	V3	V4	V5
	Visit Number				
Clinical height	flat	flat	flat	flat	flat
Clinical diameters (mm)	4x4	4x4	2x4	2x4	3x4
3D diameter (*mm*)	7.19	7.90	5.55	7.65	
3D perp diameter (*mm*)	5.81	5.27	3.47	4.40	No
3D av height (*mm*)	0.061	0.031	0.027	0.025	Photo
3D volume (*mm^3*)	1.830	0.788	0.300	0.510	
3D absol volume (*mm^3*)	1.857	1.091	0.440	0.605	

NC

Biopsy at Visit 5:
BCC, superficial

Background, 3D Absol Vol in non-tumor areas:

			MEAN	SD
0.305	0.516	0.309	**0.376**	0.121

Pt 9, Lesion 10
(back)

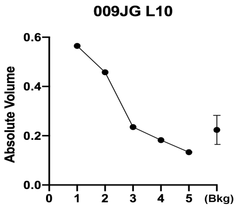

009JG L10	V1	V2	V3	V4	V5
	Visit Number				
Clinical height	flat	flat	flat	flat	flat, faint pink
Clinical diameters (mm)	4x6	4x6	4x6	2x2	2x2
3D diameter (*mm*)	8.44	5.15	3.64	3.00	--
3D perp diameter (*mm*)	3.40	2.96	1.62	1.61	--
3D av height (*mm*)	0.026	0.010	0.028	0.011	--
3D volume (*mm^3*)	0.244	0.032	0.054	0.088	0.006
3D absol volume (*mm^3*)	0.565	0.458	0.235	0.182	0.133

V3

Background, 3D Absol Vol in non-tumor areas:

			MEAN	SD
0.290	0.178	0.202	**0.224**	0.059

	Patient / Lesion (Location)	Graphical Summary	3-D lesion data	Visit 1	Visit 2	Visit 3	Visit 4	Visit 5	BCC lesion cleared at:	Biopsy result

Pt 10, Lesion 1
(temple)

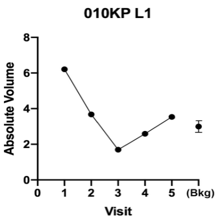

010KP L1	V1	V2	V3	V4	V5	BCC lesion cleared at					
	Visit Number										
Clinical height	slightly ra	slightly ra	flat	flat	flat, hard to find						
Clinical diameters (mm)	9x10	9x10	9x10	5x8	5x8	V3					
3D diameter (*mm*)	12.01	9.23	--	--	--						
3D perp diameter (*mm*)	9.85	7.15	--	--	--						
3D av height (*mm*)	0.068	0.023	--	--	--		Background, 3D Absol Vol in non-tumor areas:				
3D volume (*mm^3*)	5.173	2.663	-0.501	1.823	3.363					MEAN	SD
3D absol volume (*mm^3*)	6.212	3.676	1.699	2.588	3.534		2.633	3.087	3.280	3.000	0.332

Pt 10, Lesion 2
(temple)

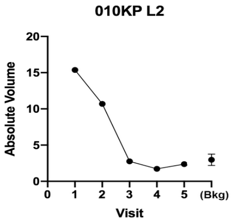

010KP L2	V1	V2	V3	V4	V5	BCC lesion cleared at					
	Visit Number										
Clinical height	slightly ra	slightly ra	raised w,	flat	flat			Biopsy at Visit 5:			
Clinical diameters (mm)	8x15	8x15	8x15	6x8	6x8	V3		Biopsy negative (no tumor)			
3D diameter (*mm*)	16.11	21.12	15.97	--	--						
3D perp diameter (*mm*)	7.56	11.51	8.49	--	--						
3D av height (*mm*)	0.019	0.063	0.008	--	--		Background, 3D Absol Vol in non-tumor areas:				
3D volume (*mm^3*)	10.984	7.556	-1.634	0.398	1.883					MEAN	SD
3D absol volume (*mm^3*)	15.372	10.692	2.763	1.721	2.376		3.860	2.386	2.662	2.970	0.783

Pt 10, Lesion 3
(cheek)

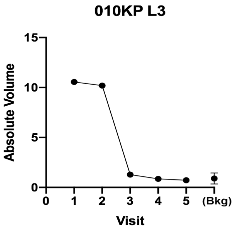

010KP L3	V1	V2	V3	V4	V5	BCC lesion cleared at					
	Visit Number										
Clinical height	slightly ra	two raise	flat	no visible	no visible lesion						
Clinical diameters (mm)	6x12	11x20	11x20	11x20	11x20	V3					
3D diameter (*mm*)	13.72	13.72	--	--	--						
3D perp diameter (*mm*)	5.56	3.52	--	--	--						
3D av height (*mm*)	0.072	0.046	--	--	--		Background, 3D Absol Vol in non-tumor areas:				
3D volume (*mm^3*)	9.087	8.800	1.068	0.709	0.188					MEAN	SD
3D absol volume (*mm^3*)	10.551	10.199	1.281	0.862	0.714		1.287	0.764	0.613	0.888	0.354

Pt 10, Lesion 4
(back)

NOTE: Although this lesion was monitored, it was only slightly raised and clinically questionable. It had normal epidermal markings on the 3D images. Biopsy showed normal skin.
We believe it was never a BCC, so this lesion was excluded from analysis.

010KP L4	V1	V2	V3	V4	V5	BCC lesion cleared at					
	Visit Number										
Clinical height	8x8	8x8	8x8	8x8	8x8	EXCLUDED THIS LESION		Biopsy at Visit 5:			
Clinical diameters (mm)	slightly ra	slightly ra	slightly r	slightly ra	barely			Normal skin			
3D diameter											
	NOT DONE (See note to the left)										
3D av height											
3D volume (*mm^3*)	7.6151	8.237	4.1949	3.5581	1.3595					MEAN	SD
3D absol volume (*mm^3*)	7.6151	8.28	4.2131	3.6211	1.3778		0.71329	0.9129	0.8643	0.8302	0.1041

Pt 10, Lesion 5
(temple)

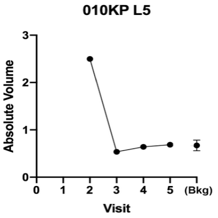

010KP L5	V1	V2	V3	V4	V5	BCC lesion cleared at					
	Visit Number										
Clinical height		raised	no lesior	no lesion	no lesion detected						
Clinical diameters (mm)		3x4	3x4	3x4	3x4	V3					
3D diameter (*mm*)		6.19	--	--	--						
3D perp diameter (*mm*)		5.69	--	--	--						
3D av height (*mm*)		0.089	--	--	--		Background, 3D Absol Vol in non-tumor areas:				
3D volume (*mm^3*)		2.343	0.176	-0.429	0.392					MEAN	SD
3D absol volume (*mm^3*)		2.498	0.537	0.641	0.687		0.540	0.737	0.737	0.671	0.113

Patient / Lesion (Location)	Graphical Summary	3-D lesion data	Visit 1	Visit 2	Visit 3	Visit 4	Visit 5	BCC lesion cleared at:	Biopsy result

11- Screenfail

Patient / Lesion (Location)	Graphical Summary	3-D lesion data	Visit 1	Visit 2	Visit 3	Visit 4	Visit 5	BCC lesion cleared at:	Biopsy result

Pt 12, Lesion 1
(shoulder)

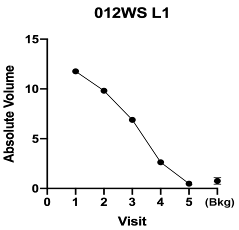

012WS L1	V1	V2	V3	V4	V5	BCC lesion cleared at					
	Visit Number										
Clinical height	flat	flat	flat	flat	gone						
Clinical diameters (mm)	10x13	10x13	10x13		No lesion	V5					
3D diameter (*mm*)	17.12	15.39	7.45	6.04	visible						
3D perp diameter (*mm*)	13.74	11.90	5.43	2.84	--						
3D av height (*mm*)	0.045	0.030	0.179	0.050	--						
3D volume (*mm^3*)	10.267	6.926	3.986	0.754	0.112		V1	V2	V5	MEAN	SD
3D absol volume (*mm^3*)	11.767	9.815	6.893	2.637	0.475		0.659	1.121	0.475	0.752	0.333

Pt 12, Lesion 2
(back)

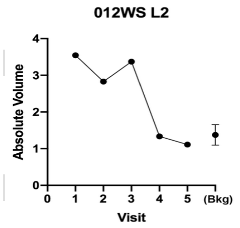

012WS L2	V1	V2	V3	V4	V5
	Visit Number				
Clinical height	flat	flat	flat	gone	
Clinical diameters (mm)	10x10	10x10	7x10		
3D diameter (*mm*)	11.81	11.32	5.16	1.99	No lesion
3D perp diameter (*mm*)	9.92	9.33	3.12	1.47	visible
3D av height (*mm*)	0.038	0.040	0.054	0.001	--
3D volume (*mm^3*)	2.276	2.253	0.866	0.891	-0.280
3D absol volume (*mm^3*)	3.544	2.828	3.373	1.334	1.110

V4

Biopsy at Visit 5:
Scar tissue

Background, 3D Absol Vol in non-tumor areas:

			MEAN	SD
1.641	1.399	1.080	**1.373**	0.281

Pt 12, Lesion 3
(shoulder)

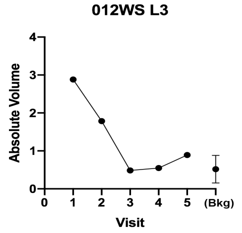

012WS L3	V1	V2	V3	V4	V5
	Visit Number				
Clinical height	flat	flat	flat	gone	
Clinical diameters (mm)	9x10	9x10	3x5		
3D diameter (*mm*)	10.40	10.00	No lesion	No lesion	No lesion
3D perp diameter (*mm*)	7.65	9.41	visible	visible	visible
3D av height (*mm*)	0.045	0.033	--	--	--
3D volume (*mm^3*)	2.588	1.674	0.307	-0.357	-0.713
3D absol volume (*mm^3*)	2.880	1.784	0.484	0.548	0.890

V3

Background, 3D Absol Vol in non-tumor areas:

			MEAN	SD
0.376	0.249	0.931	**0.519**	0.362

Pt 12, Lesion 4
(shoulder)

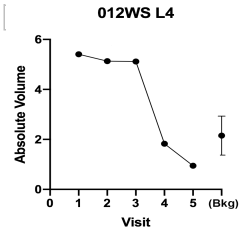

012WS L4	V1	V2	V3	V4	V5
	Visit Number				
Clinical height	flat	flat	flat	gone	
Clinical diameters (mm)	15x14	15x14	8x8		
3D diameter (*mm*)	13.13	14.15	8.59	No lesion	No lesion
3D perp diameter (*mm*)	12.13	13.35	7.36	visible	visible
3D av height (*mm*)	0.047	0.011	0.059	--	--
3D volume (*mm^3*)	4.236	-2.514	-2.469	0.696	0.331
3D absol volume (*mm^3*)	5.410	5.132	5.116	1.829	0.949

V4

Background, 3D Absol Vol in non-tumor areas:

			MEAN	SD
2.972	2.064	1.417	**2.151**	0.781

Pt 12, Lesion 5
(back)

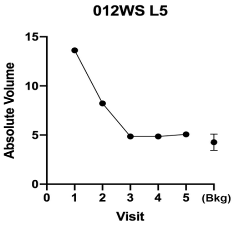

012WS L5	V1	V2	V3	V4	V5
	Visit Number				
Clinical height	flat	flat	flat	flat	Normal skin markings
Clinical diameters (mm)	15x12	15x12	10x12	8x10	8x10
3D diameter (*mm*)	16.57	17.06	6.69	No lesion	No lesion
3D perp diameter (*mm*)	14.88	13.28	3.50	visible	visible
3D av height (*mm*)	0.071	0.027	0.040	--	--
3D volume (*mm^3*)	9.276	-0.292	-2.513	-4.295	-0.637
3D absol volume (*mm^3*)	13.617	8.216	4.860	4.860	5.074

V3

Background, 3D Absol Vol in non-tumor areas:

			MEAN	SD
4.626	3.327	4.870	**4.274**	0.829

Pt 12, Lesion 6
(back)

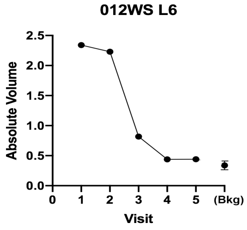

012WS L6	V1	V2	V3	V4	V5
Clinical height	flat	flat	flat	flat	Normal skin markings
Clinical diameters (mm)	11x8	11x8	7x10	6x7	8x7
3D diameter (*mm*)	11.47	10.88	1.94	No lesion	No lesion
3D perp diameter (*mm*)	8.15	7.81	1.86	visible	visible
3D av height (*mm*)	0.0154	0.0184	0.0074	--	--
3D volume (*mm^3*)	0.153	-0.100	-0.676	-0.254	-0.344
3D absol volume (*mm^3*)	2.341	2.230	0.818	0.439	0.440

V4

Background, 3D Absol Vol in non-tumor areas:

			MEAN	SD
0.356	0.365	0.297	**0.339**	0.037

Pt 12, Lesion 7
(back)

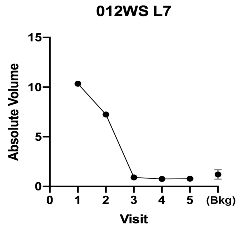

012WS L7	V1	V2	V3	V4	V5
	Visit Number				
Clinical height	flat	flat	flat	slightly d	Slightly depressed
Clinical diameters (mm)	11x7	11x7	8x11	6x9	6x9
3D diameter (*mm*)	13.48	12.80	No lesion	No lesion	No lesion
3D perp diameter (*mm*)	10.04	9.77	visible	visible	visible
3D av height (*mm*)	0.0216	0.0279	--	--	--
3D volume (*mm^3*)	-6.895	-3.455	0.091	0.083	0.652
3D absol volume (*mm^3*)	10.346	7.238	0.906	0.755	0.778

V3

Background, 3D Absol Vol in non-tumor areas:

			MEAN	SD
1.696	1.138	0.774	**1.203**	0.465

Pt 12, Lesion 8
(back)

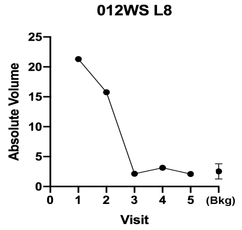

012WS L8	V1	V2	V3	V4	V5
	Visit Number				
Clinical height	flat	flat	flat	flat	3x3 slightly raised
Clinical diameters (mm)	15x9	15x9	7x10	7x10	7x10
3D diameter (*mm*)	16.21	16.40	12.76	No lesion	?Scar
3D perp diameter (*mm*)	11.75	11.93	8.87	visible	--
3D av height (*mm*)	0.0065	0.0148	0.0048	--	--
3D volume (*mm^3*)	-19.531	-12.004	-0.760	0.963	1.536
3D absol volume (*mm^3*)	21.305	15.753	2.142	3.134	2.094

V3

Biopsy at Visit 5:
Scar tissue

Background, 3D Absol Vol in non-tumor areas:

			MEAN	SD
3.984	1.993	1.594	**2.524**	1.280

Pt 12, Lesion 9
(leg)

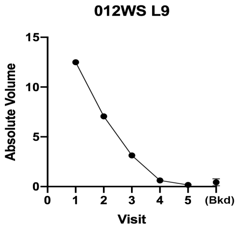

	Visit Number				
**012WS L9**	**V1**	**V2**	**V3**	**V4**	**V5**
Clinical height	flat	flat	flat	flat	normal skin
Clinical diameters (mm)	5x8	5x8	11x9	4x5	4x5
3D diameter (*mm*)	12.12	11.81	11.20	No lesion	No lesion
3D perp diameter (*mm*)	8.80	8.92	9.09	visible	visible
3D av height (*mm*)	0.1601	0.0971	0.0522	--	--
3D volume (*mm^3*)	12.406	6.953	2.965	0.607	0.169
3D absol volume (*mm^3*)	12.498	7.057	3.136	0.615	0.172

V4

Background, 3D Absol Vol in non-tumor areas:

			MEAN	SD
0.573	0.623	0.084	**0.427**	0.298

Pt 12, Lesion 10
(leg)

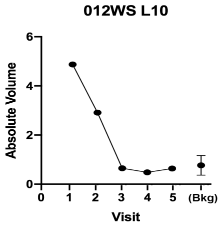

	Visit Number				
**012WS L10**	**V1**	**V2**	**V3**	**V4**	**V5**
Clinical height	flat	flat	flat	flat	normal skin
Clinical diameters (mm)	9x5	9x5	4x5	5x10	5x10
3D diameter (*mm*)	10.50	10.04	8.51	4.41	No lesion
3D perp diameter (*mm*)	6.40	8.10	5.16	2.46	visible
3D av height (*mm*)	0.0999	0.0488	0.0161	0.0076	--
3D volume (*mm^3*)	4.739	2.037	0.392	-0.147	0.509
3D absol volume (*mm^3*)	4.855	2.890	0.629	0.463	0.620

V3

Background, 3D Absol Vol in non-tumor areas:

			MEAN	SD
1.214	0.529	0.502	**0.749**	0.40

Patient / Lesion (Location)	Graphical Summary	3-D lesion data	Visit 1	Visit 2	Visit 3	Visit 4	Visit 5	BCC lesion cleared at:	Biopsy result

In retrospect, all lesions of Patient 13 turned out to be keloid scars (located on the lower legs, probably due to shaving).

Pt 13, Lesion 1
(leg)

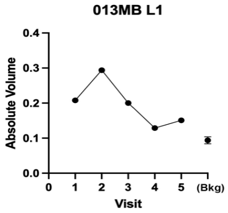

	Visit Number				
**013MB L1**	**V1**	**V2**	**V3**	**V4**	**V5**
Clinical height	Raised	raised	raised	raised	raised
Clinical diameters (mm)	3x3	2x2	2x2	1x1	
3D diameter (*mm*)	3.181	2.716	2.664	2.574	2.344
3D perp diameter (*mm*)	2.380	2.504	2.037	2.209	1.919
3D av height (*mm*)	0.049	0.081	0.068	0.044	0.061
3D volume (*mm^3*)	0.208	0.294	0.199	0.129	0.151
3D absol volume (*mm^3*)	0.208	0.294	0.200	0.129	0.151

Keloid

Background, 3D Absol Vol in non-tumor areas:

			MEAN	SD
0.1	0.083	0.1	**0.094**	0.01

Pt 13, Lesion 2
(leg)

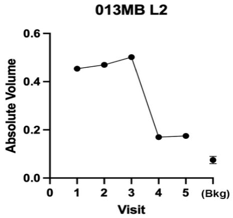

	Visit Number				
**013MB L2**	**V1**	**V2**	**V3**	**V4**	**V5**
Clinical height	Raised	raised	raised	raised	raised
Clinical diameters (mm)	3x3	3x2	3x2	2x2	
3D diameter (*mm*)	3.115	3.487	3.277	2.875	3.116
3D perp diameter (*mm*)	2.768	3.015	2.682	2.540	2.448
3D av height (*mm*)	0.094	0.076	0.095	0.044	0.037
3D volume (*mm^3*)	0.452	0.464	0.502	0.170	0.173
3D absol volume (*mm^3*)	0.454	0.470	0.502	0.170	0.175

Keloid

Biopsy at Visit 5:
Scar tissue

Background, 3D Absol Vol in non-tumor areas:

			MEAN	SD
0.072	0.067	0.086	**0.075**	0.01

Pt 13, Lesion 3
(leg)

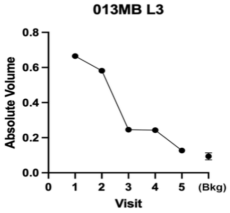

	Visit Number				
**013MB L3**	**V1**	**V2**	**V3**	**V4**	**V5**
Clinical height	Raised	raised	raised	raised	raised
Clinical diameters (mm)	3x3	3x2	3x2	2x2	
3D diameter (*mm*)	3.416	3.526	3.158	2.915	3.010
3D perp diameter (*mm*)	2.929	3.092	2.598	2.635	2.610
3D av height (*mm*)	0.111	0.082	0.050	0.062	0.027
3D volume (*mm^3*)	0.663	0.563	0.245	0.243	0.120
3D absol volume (*mm^3*)	0.665	0.582	0.246	0.243	0.127

Keloid

Background, 3D Absol Vol in non-tumor areas:

			MEAN	SD
0.1	0.083	0.1	**0.094**	0.01

Pt 13, Lesion 4
(leg)

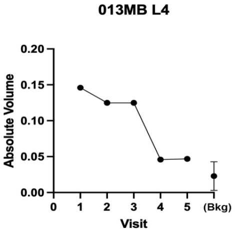

	Visit Number				
**013MB L4**	**V1**	**V2**	**V3**	**V4**	**V5**
Clinical height	Raised	raised	raised	raised	raised
Clinical diameters (mm)	1x1	1x2	1x1	1x1	
3D diameter (*mm*)	2.239	2.653	2.525	1.799	1.854
3D perp diameter (*mm*)	1.785	1.729	1.983	1.570	1.643
3D av height (*mm*)	0.065	0.049	0.037	0.034	0.031
3D volume (*mm^3*)	0.119	0.123	0.051	0.046	0.046
3D absol volume (*mm^3*)	0.146	0.125	0.125	0.046	0.047

Keloid

Background, 3D Absol Vol in non-tumor areas:

			MEAN	SD
0.026	0.025	0.018	**0.023**	0.00

Pt 13, Lesion 5
(leg)

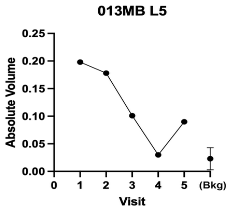

	Visit Number				
**013MB L5**	**V1**	**V2**	**V3**	**V4**	**V5**
Clinical height	Raised	raised	raised	raised	raised
Clinical diameters (mm)	1x1	2x2	2x2	1x2	
3D diameter (*mm*)	2.200	2.450	2.060	1.912	2.115
3D perp diameter (*mm*)	1.886	2.289	1.939	1.505	1.870
3D av height (*mm*)	0.087	0.052	0.046	0.028	0.042
3D volume (*mm^3*)	0.198	0.146	0.091	0.030	0.089
3D absol volume (*mm^3*)	0.198	0.178	0.101	0.030	0.090

Keloid

Background, 3D Absol Vol in non-tumor areas:

			MEAN	SD
0.026	0.025	0.018	**0.023**	0.00

Pt 13, Lesion 7
(leg)

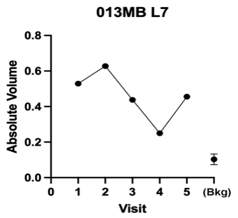

013MB L7	V1	V2	V3	V4	V5
	Visit Number				
Clinical height	Raised	raised	raised	raised	raised
Clinical diameters (mm)	3x4	4x2	3x3	3x3	
3D diameter (*mm*)	3.986	4.022	3.626	3.590	4.031
3D perp diameter (*mm*)	2.640	2.957	2.550	2.338	3.057
3D av height (*mm*)	0.092	0.093	0.083	0.060	0.063
3D volume (*mm^3*)	0.529	0.625	0.438	0.250	0.455
3D absol volume (*mm^3*)	0.529	0.628	0.438	0.250	0.456

Keloid

Biopsy at Visit 5: Scar tissue

Background, 3D Absol Vol in non-tumor areas:

			MEAN	SD
0.139	0.073	0.097	**0.103**	0.03

Pt 13, Lesion 8
(leg)

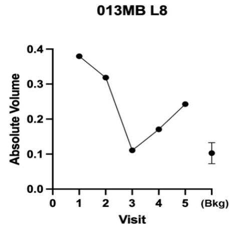

013MB L8	V1	V2	V3	V4	V5
	Visit Number				
Clinical height	raised	flat	sl. raisec	slightly raised	
Clinical diameters (mm)	4x4	2x3	2x3	1x1	
3D diameter (*mm*)	3.8166	3.7049	3.1588	3.5212	3.3915
3D perp diameter (*mm*)	2.8908	2.6884	2.5624	2.3245	3.1721
3D av height (*mm*)	0.0547	0.0537	0.0237	0.037	0.0393
3D volume (*mm^3*)	0.3794	0.3185	0.1034	0.1708	0.238
3D absol volume (*mm^3*)	0.3794	0.3185	0.1109	0.1708	0.2428

Keloid

Biopsy at Visit 5: Scar tissue

Background, 3D Absol Vol in non-tumor areas:

			MEAN	SD
0.139	0.073	0.097	**0.103**	0.03

Patient / Lesion (Location)	Graphical Summary	3-D lesion data	Visit 1	Visit 2	Visit 3	Visit 4	Visit 5	BCC lesion cleared at:	Biopsy result

Pt 14, Lesion 1
(forearm)

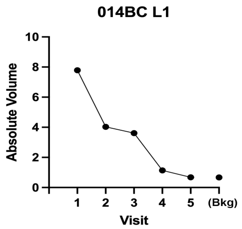

014 BC L1	V1	V2	V3	V4	V5
	Visit Number				
Clinical height	Raised	raised	raised	raised	raised
Clinical diameters (mm)	10 x 10	10 x 10	10 x 10	10 x 10	2 x 2
3D diameter (*mm*)	7.911	6.932	8.385	6.756	6.284
3D perp diameter (*mm*)	7.67	6.538	8.104	5.998	5.44
3D av height (*mm*)	0.198	0.124	0.097	0.053	0.05
3D volume (*mm^3*)	8.071	3.663	3.683	1.16	0.848
3D absol volume (*mm^3*)	8.074	3.664	3.683	1.16	0.848

V5

Background, 3D Absol Vol in non-tumor areas:

			MEAN	SD
0.733	0.382	0.728	**0.614**	0.20

Pt 14, Lesion 2
(elbow)

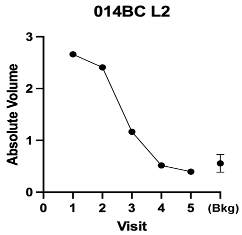

014 BC L2	V1	V2	V3	V4	V5
	Visit Number				
Clinical height	Raised	raised	raised	raised	raised
Clinical diameters (mm)	8 x 8	10 x 10	10 x 10	9 x 9	no lesion
3D diameter (*mm*)	11.257	11.289	6.955	6.913	7.048
3D perp diameter (*mm*)	9.293	7.682	5.124	5.197	5.281
3D av height (*mm*)	0.036	0.044	0.001	0.001	0.005
3D volume (*mm^3*)	2.352	2.159	0.008	0.024	0.104
3D absol volume (*mm^3*)	2.66	2.408	1.165	0.515	0.395

V4

Background, 3D Absol Vol in non-tumor areas:

			MEAN	SD
0.367	0.703	0.599	**0.556**	0.17

Pt 14, Lesion 3
(back)

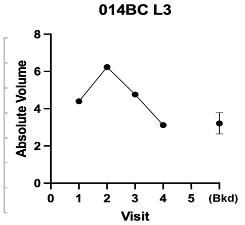

014 BC L3	V1	V2	V3	V4	V5
	Visit Number				
Clinical height	Raised	raised	raised	raised	raised
Clinical diameters (mm)	9 x 9	9 x 9	9 x 9	n/d	no lesion
3D diameter (*mm*)	14.664	13.679	13.47	13.442	
3D perp diameter (*mm*)	10.923	10.923	11.048	10.628	
3D av height (*mm*)	0.006	0.008	0.004	0.018	
3D volume (*mm^3*)	0.563	0.421	0.775	1.748	photo
3D absol volume (*mm^3*)	4.396	6.235	4.765	3.116	wrong site

V4

Background, 3D Absol Vol in non-tumor areas:

			MEAN	SD
3.761	2.629	3.241	**3.210**	0.57

Pt 14, Lesion 4
(back)

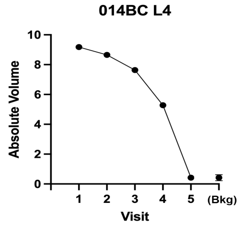

014 BC L4	V1	V2	V3	V4	V5
	Visit Number				
Clinical height	Raised	raised	raised	raised	raised
Clinical diameters (mm)	9 x 9	9 x 9	9 x 9	10 x 10	no lesion
3D diameter (*mm*)	8.591	8.456	8.46	9.041	8.937
3D perp diameter (*mm*)	7.426	6.325	5.963	4.608	4.546
3D av height (*mm*)	0.214	0.256	0.207	0.256	0.013
3D volume (*mm^3*)	8.316	8.642	7.582	5.274	0.267
3D absol volume (*mm^3*)	8.316	8.651	7.645	5.276	0.419

V5

Background, 3D Absol Vol in non-tumor areas:

			MEAN	SD
0.231	0.491	0.606	**0.443**	0.19

Pt 14, Lesion 5
(back)

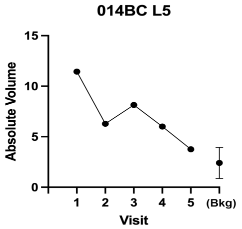

014 BC L5	V1	V2	V3	V4	V5
	Visit Number				
Clinical height	Raised	raised	raised	raised	raised
Clinical diameters (mm)	12 x 12	12 x 12	12 x 11	11 x 11	no lesion
3D diameter (*mm*)	12.93	12.349	12.995	11.378	11.405
3D perp diameter (*mm*)	11.309	10.744	10.808	7.489	6.76
3D av height (*mm*)	0.116	0.071	0.09	0.095	0.004
3D volume (*mm^3*)	11.431	6.097	8.017	5.829	0.18
3D absol volume (*mm^3*)	11.451	6.28	8.143	6.011	1.738

V5

Background, 3D Absol Vol in non-tumor areas:

			MEAN	SD
0.724	2.755	3.765	**2.415**	1.55

Pt 14, Lesion 6
(arm)

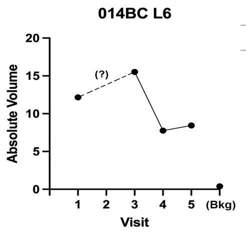

014 BC L6	V1	V2	V3	V4	V5
	Visit Number				
Clinical height	Raised	raised	raised	raised	raised
Clinical diameters (mm)	11 x 11	11 x 11	8 x 4	11 x 11	6 x 6
3D diameter (*mm*)	9.671	n/d	9.291	7.727	5.329
3D perp diameter (*mm*)	6.913	n/d	6.129	4.47	3.857
3D av height (*mm*)	0.254	n/d	0.448	0.328	0.608
3D volume (*mm^3*)	12.147	n/d	15.394	7.745	8.453
3D absol volume (*mm^3*)	12.152	n/d	15.522	7.758	8.458

NC

Biopsy at Visit 5: BCC, superficial, nodular, and micronodular

Background, 3D Absol Vol in non-tumor areas:

			MEAN	SD
0.429	0.45	0.288	**0.389**	0.09

Pt 14, Lesion 7
(shoulder)

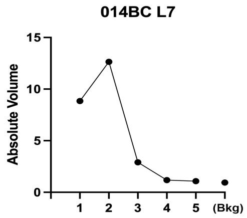

014 BC L7	V1	V2	V3	V4	V5
	Visit Number				
Clinical height	Raised	raised	raised		
Clinical diameters (mm)	10 x 10	10 x 10	3 x 3	4 x 4	no lesion
3D diameter (*mm*)	14.723	14.663	11.365	7.283	7.129
3D perp diameter (*mm*)	10.012	9.388	8.864	5.892	5.754
3D av height (*mm*)	0.095	0.144	0.047	0.022	0.035
3D volume (*mm^3*)	8.467	12.529	2.665	0.549	0.793
3D absol volume (*mm^3*)	8.849	12.648	2.91	1.178	1.075

V5

Background, 3D Absol Vol in non-tumor areas:

			MEAN	SD
0.941	0.951	0.643	**0.845**	0.18

Pt 14, Lesion 8
(chest)

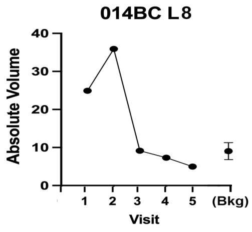

014 BC L8	V1	V2	V3	V4	V5
	Visit Number				
Clinical height	only slightly raised				
Clinical diameters (mm)	24 x 13	24 x 13	17 x 12	17 x 17	no lesion
3D diameter (*mm*)	23.851	22.474	22.656	16.8	16.282
3D perp diameter (*mm*)	13.972	15.821	13.124	11.304	10.95
3D av height (*mm*)	0.027	0.134	0.011	0.004	0.011
3D volume (*mm^3*)	6.98	34.911	2.238	0.498	1.288
3D absol volume (*mm^3*)	24.727	35.987	9.169	7.308	4.975

V3

Background, 3D Absol Vol in non-tumor areas:

			MEAN	SD
10.973	9.616	6.599	**9.063**	2.24

Pt 14, Lesion 9
(chest)

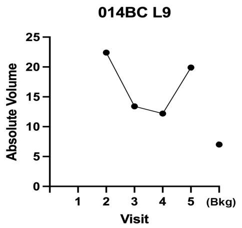

014 BC L9	V1	V2	V3	V4	V5
	Visit Number				
Clinical height	Raised	raised	raised	raised	raised
Clinical diameters (mm)	n/d	8 x 8	9 x 9	9 x 9	8 x 8
3D diameter (*mm*)	n/d	14.392	11.354	9.711	10.996
3D perp diameter (*mm*)	n/d	10.75	8.76	8.325	10.036
3D av height (*mm*)	n/d	0.216	0.191	0.232	0.284
3D volume (*mm^3*)	n/d	22.367	13.406	12.224	19.87
3D absol volume (*mm^3*)	n/d	22.369	13.422	12.234	19.881

NC

Biopsy at Visit 5: BCC, superficial, nodular, and trichoepithelial

Background, 3D Absol Vol in non-tumor areas:

			MEAN	SD
8.259	5.147	8.584	**7.330**	1.90

Pt 14, Lesion 10
(nose)

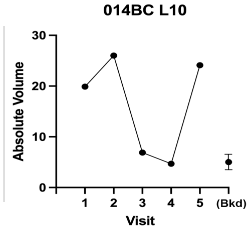

014 BC L10	V1	V2	V3	V4	V5
	Visit Number				
Clinical height	Raised	raised	raised	raised	raised
Clinical diameters (mm)	15 x 8	15 x 8	8 x 4	6 x 6	10 x 10
3D diameter (*mm*)	12.899	12.547	9.177	8.064	12.286
3D perp diameter (*mm*)	7.701	8.654	5.056	4.622	7.646
3D av height (*mm*)	0.297	0.378	0.233	0.171	0.381
3D volume (*mm^3*)	8.91	10.71	14.64	13.53	12.21
3D absol volume (*mm^3*)	19.87	26.026	6.893	4.686	24.118

NC

Clinically, this was clearly a big nodular BCC; patient refused biopsy

Background, 3D Absol Vol in non-tumor areas:

			MEAN	SD
3.309	5.793	6.022	**5.041**	1.50

Patient / Lesion (Location)	Graphical Summary	3-D lesion data	Visit 1	Visit 2	Visit 3	Visit 4	Visit 5	BCC lesion cleared at:	Biopsy result

Pt 15, Lesion 1
(scalp)

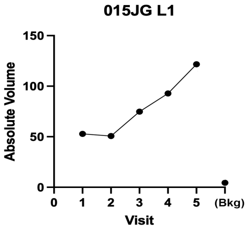

015 JG L1	V1	V2	V3	V4	V5
	Visit Number				
Clinical height	Raised	raised	raised	raised	raised
Clinical diameters (mm)	9 x 9	9 x 9	10 x 10	11 x 10	12 x 11
3D diameter (*mm*)	9.967	9.976	11.114	12.056	12.2
3D perp diameter (*mm*)	8.485	9.003	10.764	10.77	11.139
3D av height (*mm*)	0.928	0.848	0.985	1.052	1.286
3D volume (*mm^3*)	52.774	50.471	74.314	92.616	121.52
3D absol volume (*mm^3*)	52.803	50.639	74.795	92.754	121.68

NC

Background, 3D Absol Vol in non-tumor areas:

			MEAN	SD
2.206	5.775	5.37	**4.450**	1.95

Pt 15, Lesion 2
(scalp)

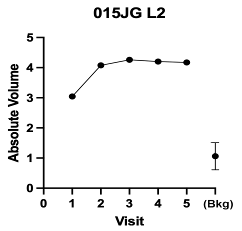

015 JG L2	V1	V2	V3	V4	V5
	Visit Number				
Clinical height	Raised	raised	raised	raised	raised
Clinical diameters (mm)	5 x 5	5 x 5	4 x 4	3 x 2	3 x 3
3D diameter (*mm*)	7.272	5.924	5.987	5.14	5.293
3D perp diameter (*mm*)	6.328	4.708	5.462	4.893	5.058
3D av height (*mm*)	0.113	0.212	0.194	0.267	0.247
3D volume (*mm^3*)	3.044	4.058	4.255	4.202	4.161
3D absol volume (*mm^3*)	3.044	4.076	4.259	4.202	4.174

NC

Background, 3D Absol Vol in non-tumor areas:

			MEAN	SD
0.715	0.895	1.57	**1.060**	0.45

Pt 15, Lesion 3
(temple)

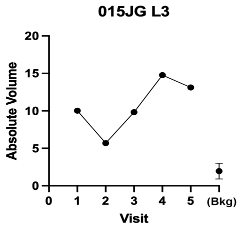

015 JG L3	Visit Number				
	V1	V2	V3	V4	V5
Clinical height	Raised	raised	raised	raised	raised
Clinical diameters (mm)	8 x 6	9 x 6	7 x 4	10 x 5	13 x 9
3D diameter (*mm*)	10.657	10.507	12.624	14.06	13.41
3D perp diameter (*mm*)	9.429	7.219	9.245	9.081	6.851
3D av height (*mm*)	0.178	0.093	0.146	0.212	0.223
3D volume (*mm^3*)	9.78	4.312	9.113	14.772	13.102
3D absol volume (*mm^3*)	10.032	5.69	9.833	14.783	13.132

NC

Biopsy at Visit 5: BCC, superficial, nodular, and trichoepitheliom.

Background, 3D Absol Vol in non-tumor areas:

			MEAN	SD
3.164	1.393	1.302	**1.953**	1.05

Pt 15, Lesion 4
(nose)

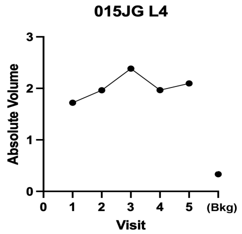

015 JG L4	Visit Number				
	V1	V2	V3	V4	V5
Clinical height	Raised	raised	raised	raised	raised
Clinical diameters (mm)	2 x 2	2 x 2	3 x 3	3 x 3	4 x 4
3D diameter (*mm*)	3.998	3.767	4.108	3.672	3.606
3D perp diameter (*mm*)	3.121	3.228	3.789	3.585	3.477
3D av height (*mm*)	0.203	0.242	0.258	0.232	0.23
3D volume (*mm^3*)	1.704	1.963	2.379	1.967	2.017
3D absol volume (*mm^3*)	1.722	1.963	2.384	1.967	2.096

NC

Background, 3D Absol Vol in non-tumor areas:

			MEAN	SD
0.328	0.373	0.298	**0.333**	0.04

Pt 15, Lesion 5
(nose)

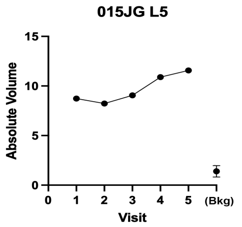

015 JG L5	Visit Number				
	V1	V2	V3	V4	V5
Clinical height	Raised	raised	raised	raised	raised
Clinical diameters (mm)	4 x 3	4 x 4	4 x 4	5 x 5	
3D diameter (*mm*)	5.369	5.349	5.485	7.127	6.6
3D perp diameter (*mm*)	5.149	5.247	5.105	6.06	5.975
3D av height (*mm*)	0.426	0.408	0.377	0.309	0.395
3D volume (*mm^3*)	8.712	8.176	8.11	9.726	11.385
3D absol volume (*mm^3*)	8.734	8.227	9.062	10.896	11.567

NC

Background, 3D Absol Vol in non-tumor areas:

			MEAN	SD
1.181	0.957	2.058	**1.399**	0.58

Pt 15, Lesion 6
(nose)

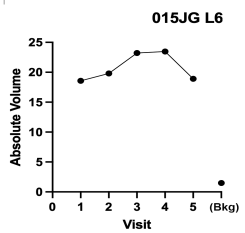

015 JG L6	Visit Number				
	V1	V2	V3	V4	V5
Clinical height	Raised	raised	raised	raised	raised
Clinical diameters (mm)	5 x 5	5 x 5	6 x 4	6 x 5	5 x 5
3D diameter (*mm*)	6.48	7.311	7.634	7.793	7.35
3D perp diameter (*mm*)	6.326	6.741	6.712	6.652	6.389
3D av height (*mm*)	0.661	0.568	0.647	0.597	0.546
3D volume (*mm^3*)	18.582	19.798	23.199	23.346	18.87
3D absol volume (*mm^3*)	18.582	19.798	23.228	23.464	18.912

NC

Background, 3D Absol Vol in non-tumor areas:

			MEAN	SD
1.988	1.246	1.235	**1.490**	0.43

Pt 15, Lesion 7
(ear)

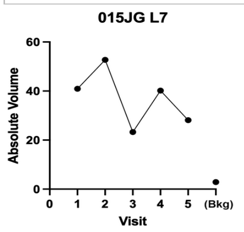

015 JG L7	Visit Number				
	V1	V2	V3	V4	V5
Clinical height	Raised	raised	raised	raised	raised
Clinical diameters (mm)	14 x 5	14 x 5	11 x 8	6 x 5	15 x 8
3D diameter (*mm*)	12.026	11.952	11.443	10.897	12.386
3D perp diameter (*mm*)	6.706	7.295	6.965	7.468	7.29
3D av height (*mm*)	0.694	0.85	0.465	0.728	0.527
3D volume (*mm^3*)	40.852	52.705	23.225	40.199	27.914
3D absol volume (*mm^3*)	40.915	52.705	23.283	40.199	28.108

NC

Background, 3D Absol Vol in non-tumor areas:

			MEAN	SD
3.169	2.027	3.444	**2.880**	0.75

Pt 15, Lesion 8
(back)

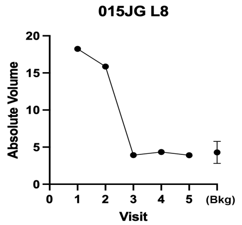

015 JG L8	Visit Number				
	V1	V2	V3	V4	V5
Clinical height	Raised	raised	raised		
Clinical diameters (mm)	14 x 13	14 x 13	10 x 10	4 x 3	no lesion
3D diameter (*mm*)	16.26	16.564	11.262	11.101	11.253
3D perp diameter (*mm*)	13.004	14.843	9.6	9.814	9.863
3D av height (*mm*)	0.11	0.088	0.029	0.055	0.049
3D volume (*mm^3*)	17.824	15.588	2.219	4.138	3.71
3D absol volume (*mm^3*)	18.234	15.855	3.925	4.336	3.914

V3

Background, 3D Absol Vol in non-tumor areas:

			MEAN	SD
5.303	4.975	2.587	**4.288**	1.48

Pt 15, Lesion 9
(shoulder)

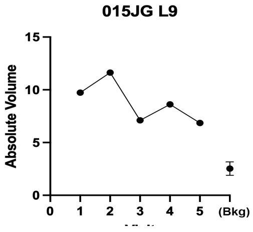

015 JG L9	Visit Number				
	V1	V2	V3	V4	V5
Clinical height	Raised	raised	raised	raised	raised
Clinical diameters (mm)	7 x 5	6 x 5	6 x 4	6 x 5	4 x 4
3D diameter (*mm*)	9.147	9.006	8.882	9.113	9.183
3D perp diameter (*mm*)	7.344	7.198	6.974	7.233	7.118
3D av height (*mm*)	0.2	0.248	0.147	0.164	0.134
3D volume (*mm^3*)	9.366	12.026	7.441	7.815	6.421
3D absol volume (*mm^3*)	9.723	11.616	7.063	8.601	6.809
3D absol volume	9.731	11.636	7.106	8.616	6.861

NC

Biopsy at Visit 5: BCC, superficial, nodular, and micronodular

Background, 3D Absol Vol in non-tumor areas:

			MEAN	SD
3.243	2.002	2.334	**2.526**	0.64

Visit

Pt 15, Lesion 10 (back)

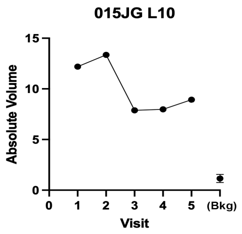

015 JG L10	V1	V2	V3	V4	V5
	Visit Number				
Clinical height	Raised	raised	raised	raised	raised
Clinical diameters (mm)	7 x 5	8 x 4	9 x 5	8 x 5	12 x 5
3D diameter (*mm*)	8.957	9.079	8.925	8.586	9.329
3D perp diameter (*mm*)	7.982	6.071	5.243	5.829	6.132
3D av height (*mm*)	0.283	0.35	0.239	0.228	0.243
3D volume (*mm^3*)	12.116	13.309	7.867	7.94	8.935
3D absol volume (*mm^3*)	12.194	13.361	7.876	7.986	8.937

NC

Biopsy at Visit 5: BCC, nodular & trichoepithelio-matous subtypes

Background, 3D Absol Vol in non-tumor areas:

			MEAN	SD
1.422	1.341	0.694	1.152	0.40

ARIZONA PATIENTS:

Patient / Lesion (Location)	Graphical Summary	3-D lesion data	Visit 1	Visit 2	Visit 3	Visit 4	Visit 5	BCC lesion cleared at:	Biopsy result

Patient 26

**Lesion 1 (cheek)**	DATA NOT USABLE- Problem with 3D reconstruction, uable to process due to hair artifacts
**Lesion 2 (cheek)**	DATA NOT USABLE- tumor already gone at Visit 2
**Lesion 3 (nose)**	DATA NOT USABLE- tumor already gone at Visit 2
**Lesion 4 (ear)**	DATA NOT USABLE- tumor already gone at Visit 2
**Lesion 5 (leg)**	DATA NOT USABLE- tumor already gone at Visit 2
**Lesion 6 (leg)**	DATA NOT USABLE- Problem with 3D reconstruction, uable to process due to hair artifacts
**Lesion 7 (leg)**	DATA NOT USABLE- Images from Visits 4 and 5 missing
**Lesion 8 (leg)**	DATA NOT USABLE- Problem with 3D reconstruction, uable to process due to hair artifacts
**Lesion 9 (leg)**	DATA NOT USABLE- tumor already gone at Visit 2

Patient / Lesion (Location)	Graphical Summary	3-D lesion data	Visit 1	Visit 2	Visit 3	Visit 4	Visit 5	BCC lesion cleared at:	Biopsy result

Patient 27

**Lesion 1 (ear)**	DATA NOT USABLE- tumor already gone at Visit 2
**Lesion 2 (temple)**	DATA NOT USABLE- Problem with bad 3D reconstruction, unable to process
**Lesion 3 (neck)**	DATA NOT USABLE- tumor already gone at Visit 2

Patient / Lesion (Location)	Graphical Summary	3-D lesion data	Visit 1	Visit 2	Visit 3	Visit 4	Visit 5	BCC lesion cleared at:	Biopsy result

Pt 28, Lesion 3 (back)

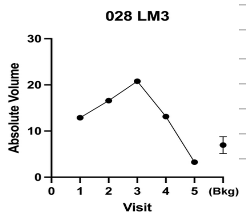

028 LM 3	V1	V2	V3	V4	V5
	Visit Number				
3D diameter (*mm*)	15.208	13.259	12.998	36.48	11.706
3D perp diameter (*mm*)	10.527	10.408	10.578	93.198	8.33
3D av height (*mm*)	0.015	0	0.002	3.397	0.033
3D volume (*mm^3*)	1.813	0.03	0.188	-0.011	2.081
3D absol volume (*mm^3*)	12.887	16.599	20.789	13.139	3.263

V5

Background, 3D Absol Vol in non-tumor areas:

			MEAN	SD
8.599	5.003	7.29	6.964	1.82

**Pt 28, Les 1 (shoulder)**	DATA NOT USABLE- tumor already gone at Visit 2
**Pt 28, Lesion 2 (back)**	DATA NOT USABLE- unhealed Bx site replaced by scar at Visit 3
**Pt 28, Lesion 4 (back)**	DATA NOT USABLE- flat white scar, tumor visually gone by Visit 3
**Pt 28, Lesion 5 (back)**	DATA NOT USABLE- Flat red biopsy site at V1 and V2; normal epidermal markings
**Pt 28, Les 6 (shoulder)**	DATA NOT USABLE- Flat white biopsy site at V1 and V2
**Pt 28, Les 7 (shoulder)**	DATA NOT USABLE- tumor already gone at Visit 2
**Pt 28, Les 8 (sternum)**	DATA NOT USABLE- tumor already gone at Visit 2

Patient / Lesion (Location)	Graphical Summary	3-D lesion data	Visit 1	Visit 2	Visit 3	Visit 4	Visit 5	BCC lesion cleared at:	Biopsy result

Patient 29

**Pt 29, Les 1 (shoulder)**	DATA NOT USABLE- tumor already gone at Visit 2
**Pt 29, Les 2 (forehead)**	DATA NOT USABLE- tumor already gone at Visit 2
**Pt 29, Les 3 (forehead)**	DATA NOT USABLE- tumor already gone at Visit 2

Graphical data	Graphical Summary	3-D lesion data	Visit 1	Visit 2	Visit 3	Visit 4	Visit 5	BCC lesion cleared at:	Biopsy result

Patient 30 (WITHDREW FROM STUDY)

Patient / Lesion (Location)	Graphical Summary	3-D lesion data	Visit 1	Visit 2	Visit 3	Visit 4	Visit 5	BCC lesion cleared at:	Biopsy result

Pt 31, Lesion 3 (leg)

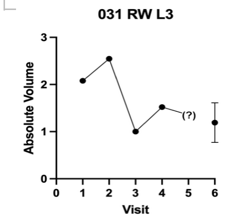

031 RW L3	V1	V2	V3	V4	V5
	Visit Number				
3D diameter (*mm*)	9.361	9.353	8.532	8.229	Corrupted
3D perp diameter (*mm*)	6.497	6.885	6.104	6.241	image
3D av height (*mm*)	0.051	0.059	0.031	0.047	0
3D volume (*mm^3*)	2.076	2.545	0.95	1.449	0
3D absol volume (*mm^3*)	2.078	2.545	1.002	1.52	0

V3

Background, 3D Absol Vol in non-tumor areas:

			MEAN	SD
1.599	1.204	0.769	1.191	0.42

Pt 31, Lesion 1 (ankle) DATA NOT USABLE- tumor already gone at Visit 2
Pt 31, Lesion 2 (leg) DATA NOT USABLE- tumor already gone at Visit 2
Pt 31, Lesion 4 (leg) DATA NOT USABLE- tumor already gone at Visit 2

Patient / Lesion (Location)	Graphical Summary	3-D lesion data	Visit 1	Visit 2	Visit 3	Visit 4	Visit 5	BCC lesion cleared at:	Biopsy result

Patient 32 (LOST TO FOLLOW-UP)

Patient / Lesion (Location)	Graphical Summary	3-D lesion data	Visit 1	Visit 2	Visit 3	Visit 4	Visit 5	BCC lesion cleared at:	Biopsy result

Patient 33

Pt 33, Lesion 1 (hand) DATA NOT USABLE- tumor already gone at Visit 2
Pt 33, Lesion 2 (ear) DATA NOT USABLE- tumor already gone at Visit 2
Pt 33, Lesion 3 (back) DATA NOT USABLE- tumor already gone at Visit 2
Pt 33, Lesion 4 (neck) DATA NOT USABLE- tumor already gone at Visit 2

(not done)

Patient / Lesion (Location)	Graphical Summary	3-D lesion data	Visit 1	Visit 2	Visit 3	Visit 4	Visit 5	BCC lesion cleared at:	Biopsy result

Pt 34, Lesion 1 (neck)

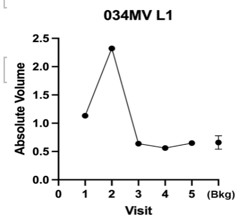

034 MV L1	V1	V2	V3	V4	V5
	Visit Number				
3D diameter (*mm*)	7.426	8.842	6.829	7.393	7.218
3D perp diameter (*mm*)	5.409	6.832	4.181	4.576	4.590
3D av height (*mm*)	0.024	0.029	0.004	0.005	0.023
3D volume (*mm^3*)	0.774	1.218	0.072	0.108	0.54
3D absol volume (*mm^3*)	1.132	2.321	0.638	0.561	0.649

BCC lesion cleared at: **V3**

Background, 3D Absol Vol in non-tumor areas:

			MEAN	SD
0.647	0.54	0.789	**0.659**	0.12

Pt 34, Lesion 2 (back) DATA NOT USABLE- tumor already gone at Visit 2
Pt 34, Les 2 (forearm) DATA NOT USABLE- tumor already gone at Visit 2

Patient / Lesion (Location)	Graphical Summary	3-D lesion data	Visit 1	Visit 2	Visit 3	Visit 4	Visit 5	BCC lesion cleared at:	Biopsy result

Pt 35, Lesion 1 (shoulder)

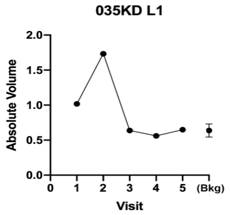

035KD L1	V1	V2	V3	V4	V5
	Visit Number				
3D diameter (*mm*)	7.426	8.842	6.829	7.393	7.218
3D perp diameter (*mm*)	5.409	6.832	4.181	4.576	4.59
3D av height (*mm*)	0.024	0.029	0.004	0.005	0.023
3D volume (*mm^3*)	0.774	1.218	0.072	0.108	0.54
3D absol volume (*mm^3*)	1.132	2.321	0.638	0.561	0.649

BCC lesion cleared at: **V3**

Background, 3D Absol Vol in non-tumor areas:

			MEAN	SD
0.647	0.540	0.726	**0.638**	0.093

Pt 35, Lesion 4 (back)

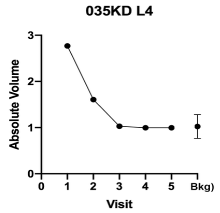

035KD L4	V1	V2	V3	V4	V5
	Visit Number				
3D diameter (*mm*)	6.85	5.86	4.51	4.12	3.05
3D perp diameter (*mm*)	5.41	4.86	3.45	3.17	2.27
3D av height (*mm*)	0.095	0.136	0.004	0.050	0.006
3D volume (*mm^3*)	1.873	1.544	0.913	0.804	-0.736
3D absol volume (*mm^3*)	2.768	1.607	1.028	0.995	0.966

BCC lesion cleared at: **V3**

Background, 3D Absol Vol in non-tumor areas:

			MEAN	SD
1.320	0.869	0.880	**1.023**	0.257

Pt 35, Lesion 2 (back) DATA NOT USABLE- tumor already gone at Visit 2
Pt 35, Lesion 3 (back) DATA NOT USABLE- tumor already gone at Visit 2
Pt 35, Lesion 5 (elbow) DATA NOT USABLE- small ulcer at V1, tumor already gone at Visit 2
Pt 35, Les 6 (clavicle) DATA NOT USABLE- photos for V1 and V2 missing

Patient / Lesion (Location)	Graphical Summary	3-D lesion data	Visit 1	Visit 2	Visit 3	Visit 4	Visit 5	BCC lesion cleared at:	Biopsy result

Pt 36, Lesion 1 (shoulder)

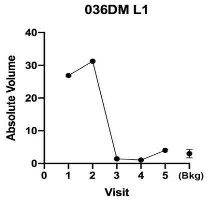

036DM L1	V1	V2	V3	V4	V5
	Visit Number				
3D diameter (*mm*)	27.354	22.394	11.001	10.732	11.721
3D perp diameter (*mm*)	11.96	12.798	9.247	9.096	9.357
3D av height (*mm*)	0.073	0.083	0.015	0.009	0.048
3D volume (*mm^3*)	7.309	5.209	0.288	-0.092	2.486
3D absol volume (*mm^3*)	26.886	31.257	1.416	1.041	4.012

BCC lesion cleared at: **V3**

Background, 3D Absol Vol in non-tumor areas:

			MEAN	SD
2.580	4.464	1.981	**3.008**	1.295

Pt 36, Lesion 2 (cheek)

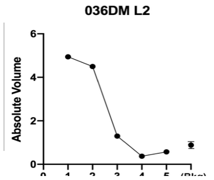

036DM L2	V1	V2	V3	V4	V5
	Visit Number				
3D diameter (*mm*)	12.94	13.131	6.566	6.884	7.133
3D perp diameter (*mm*)	6.791	8.137	4.445	4.497	4.654
3D av height (*mm*)	0.038	0.044	0.014	0.007	0.002
3D volume (*mm^3*)	2.076	2.798	0.285	0.142	0.038
3D absol volume (*mm^3*)	4.942	4.098	1.298	0.374	0.57

BCC lesion cleared at: **V4**

Background, 3D Absol Vol in non-tumor areas:

			MEAN	SD
1.051	0.861	0.730	**0.881**	0.162

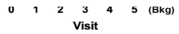

Pt 36, Lesion 3
(temple)

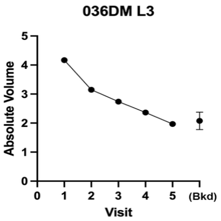

036DM L3	Visit Number V1	V2	V3	V4	V5
3D diameter (*mm*)	10.284	12.309	9.582	9.58	9.82
3D perp diameter (*mm*)	9.27	9.992	7.753	7.366	7.608
3D av height (*mm*)	0.032	0.025	0.027	0.044	0.016
3D volume (*mm^3*)	2.422	1.894	1.487	2.245	0.858
3D absol volume (*mm^3*)	4.169	3.149	2.738	2.364	1.968

V4

Background, 3D Absol Vol in non-tumor areas:

			MEAN	SD
2.208	2.291	1.731	**2.077**	0.302

Graphical data	Notes & Response to VitD	3-D lesion data	Visit 1	Visit 2	Visit 3	Visit 4	Visit 5	BCC lesion cleared at:	Biopsy result
**Patient 37**	(LOST TO FOLLOW-UP)								

Patient / Lesion (Location)	Graphical Summary	3-D lesion data	Visit 1	Visit 2	Visit 3	Visit 4	Visit 5	BCC lesion cleared at:	Biopsy result

Pt 38, Lesion 1
(leg)

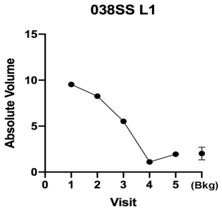

038SS L1	Visit Number V1	V2	V3	V4	V5
3D diameter (*mm*)	11.19	12.12	11.32	7.42	7.11
3D perp diameter (*mm*)	8.09	7.92	6.72	5.82	4.88
3D av height (*mm*)	0.173	0.121	0.098	0.029	0.048
3D volume (*mm^3*)	9.483	8.255	5.523	0.696	1.914
3D absol volume (*mm^3*)	9.529	8.258	5.525	1.106	1.944

V4

Background, 3D Absol Vol in non-tumor areas:

			MEAN	SD
2.617	2.190	1.260	**2.022**	0.694

Pt 38, Lesion 2
(leg)

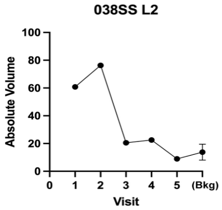

038SS L2	Visit Number V1	V2	V3	V4	V5
3D diameter (*mm*)	19.026	22.528	19.052	20.137	17.464
3D perp diameter (*mm*)	16.269	18.812	15.544	16.572	15.432
3D av height (*mm*)	0.2783	0.282	0.103	0.117	0.021
3D volume (*mm^3*)	58.247	76.242	19.406	22.333	3.275
3D absol volume (*mm^3*)	60.787	76.32	20.503	22.581	8.924

V5

Background, 3D Absol Vol in non-tumor areas:

				MEAN	SD
16.542	7.099	17.667	16.504	**14.453**	4.932

Pt 38, Lesion 3
(leg)

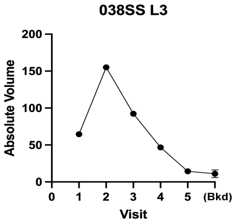

038SS L3	Visit Number V1	V2	V3	V4	V5
3D diameter (*mm*)	21.66	21.58	20.81	19.38	18.37
3D perp diameter (*mm*)	12.53	18.87	20.55	15.64	18.31
3D av height (*mm*)	0.338	0.575	0.372	0.223	0.114
3D volume (*mm^3*)	64.105	155.085	92.161	46.333	13.893
3D absol volume (*mm^3*)	64.608	155.087	92.242	46.827	14.335

V5

Background, 3D Absol Vol in non-tumor areas:

				MEAN	SD
9.075	9.476	12.326	13.742	**11.155**	2.252

Pt 38, Lesion 4 (leg) DATA NOT USED- Lesion that persisted at Vist 5 but was not biopsied

Patient / Lesion (Location)	Graphical Summary	3-D lesion data	Visit 1	Visit 2	Visit 3	Visit 4	Visit 5	BCC lesion cleared at:	Biopsy result

Pt 39, Lesion 1
(forehead)

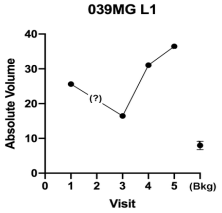

039MG L1	Visit Number V1	V2	V3	V4	V5
3D diameter (*mm*)	15.17		14.73	11.17	16.39
3D perp diameter (*mm*)	11.17	Photo	10.86	8.85	12.95
3D av height (*mm*)	0.727	missing	0.426	0.506	0.669
3D volume (*mm^3*)	25.139		16.393	31.047	35.810
3D absol volume (*mm^3*)	25.628		16.452	31.054	36.448

NC

BCC, nodular and superficial

Background, 3D Absol Vol in non-tumor areas:

			MEAN	SD
8.009	9.191	6.794	**7.998**	1.199

Pt 39, Lesion 2
(glabella)

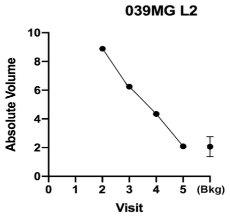

039MG L2	Visit Number V1	V2	V3	V4	V5
3D diameter (*mm*)		14.207	13.483	10.443	10.073
3D perp diameter (*mm*)	Photo	11.299	8.57	8.459	7.832
3D av height (*mm*)	missing	0.0742	0.075	0.065	0.021
3D volume (*mm^3*)		7.030	4.849	3.095	0.006
3D absol volume (*mm^3*)		8.610	6.246	4.347	2.092

V5

Background, 3D Absol Vol in non-tumor areas:

			MEAN	SD
2.865	1.711	1.621	**2.066**	0.694

Pt 39, Les 3 (forehead) DATA NOT USABLE- bad 3D reconstruction due to interference from hair

Pt 39, Lesion 4 (back) DATA NOT USABLE- photos missing for V2 and V3

Patient / Lesion (Location)	Graphical Summary	3-D lesion data	Visit 1	Visit 2	Visit 3	Visit 4	Visit 5	BCC lesion cleared at:	Biopsy result
**Patient 40**	Screen fail (not enrolled)								

Patient / Lesion (Location)	Graphical Summary	3-D lesion data	Visit 1	Visit 2	Visit 3	Visit 4	Visit 5	BCC lesion cleared at:	Biopsy result

Pt 41, Lesion 1
(back)

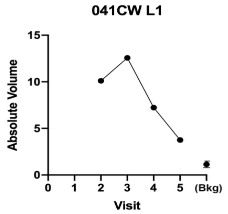

041CW L1	V1	V2	V3	V4	V5
	Visit Number				
3D diameter (*mm*)		10.38	10.02	8.79	13.55
3D perp diameter (*mm*)	Photo	8.72	7.82	5.92	9.58
3D av height (*mm*)	missing	0.168	0.230	0.123	0.101
3D volume (*mm^3*)		9.862	12.517	6.987	3.611
3D absol volume (*mm^3*)		10.104	12.573	7.228	3.755

BCC lesion cleared at: **NC**

Biopsy result: BCC, nodular (Biopsied at V5)

Background, 3D Absol Vol in non-tumor areas:

			MEAN	SD
0.912	1.547	0.960	**1.140**	0.354

Pt 41, Lesion 2
(shoulder)

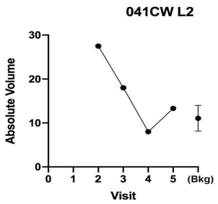

041CW L2	V1	V2	V3	V4	V5
	Visit Number				
3D diameter (*mm*)		23.873	17.933	19.587	19.105
3D perp diameter (*mm*)	Photo	19.931	15.3	17.299	18.032
3D av height (*mm*)	missing	0.091	0.07	0.031	0.062
3D volume (*mm^3*)		25.965	13.33	5.142	12.297
3D absol volume (*mm^3*)		27.482	16.424	8.015	13.31

BCC lesion cleared at: **V4**

Background, 3D Absol Vol in non-tumor areas:

			MEAN	SD
7.738	16.296	13.196	**12.410**	4.333

Pt 41, Lesion 3
(neck)

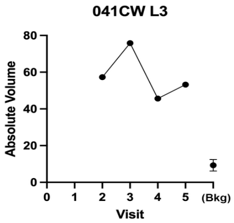

041CW L3	V1	V2	V3	V4	V5
	Visit Number				
3D diameter (*mm*)		17.144	20.512	17.177	16.046
3D perp diameter (*mm*)	Photo	13.246	14.907	13.045	12.496
3D av height (*mm*)	missing	0.349	0.358	0.287	0.37
3D volume (*mm^3*)		56.832	75.679	45.195	53.162
3D absol volume (*mm^3*)		57.309	75.856	45.62	53.223

BCC lesion cleared at: **NC**

Background, 3D Absol Vol in non-tumor areas:

			MEAN	SD
12.778	8.337	6.792	**9.302**	3.108

Pt 41, Lesion 4
(shoulder)

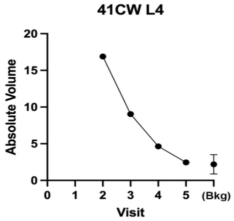

041CW L4	V1	V2	V3	V4	V5
	Visit Number				
3D diameter (*mm*)		15.11	15.646	13.907	13.42
3D perp diameter (*mm*)	Photo	5.566	4.486	4.453	3.67
3D av height (*mm*)	missing	0.328	0.184	0.08	0.06
3D volume (*mm^3*)		16.75	8.761	3.1	1.95
3D absol volume (*mm^3*)		16.899	9.041	4.641	2.462

BCC lesion cleared at: **V5**

Background, 3D Absol Vol in non-tumor areas:

			MEAN	SD
3.522	2.165	0.868	**2.185**	1.327

Pt 41, Lesion 5
(arm)

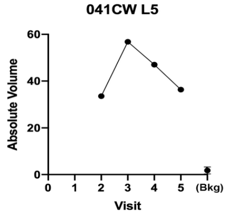

041CW L5	V1	V2	V3	V4	V5
	Visit Number				
3D diameter (*mm*)		13.05	13.37	14.85	13.43
3D perp diameter (*mm*)	Photo	11.43	10.91	9.97	9.21
3D av height (*mm*)	missing	0.501	0.505	0.391	0.806
3D volume (*mm^3*)		33.240	56.346	46.942	36.291
3D absol volume (*mm^3*)		33.578	56.818	47.035	36.322

BCC lesion cleared at: **NC**

Background, 3D Absol Vol in non-tumor areas:

			MEAN	SD
2.063	1.419	1.891	**1.791**	0.333

Patient / Lesion (Location)	Graphical Summary	3-D lesion data	Visit 1	Visit 2	Visit 3	Visit 4	Visit 5	BCC lesion cleared at:	Biopsy result

Pt 42, Lesion 1
(shoulder)

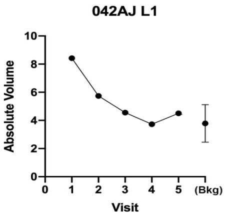

042AJ L1	V1	V2	V3	V4	V5
	Visit Number				
3D diameter (*mm*)	10.55	9.87	8.62	8.75	No lesion
3D perp diameter (*mm*)	9.60	8.57	7.57	8.15	visible
3D av height (*mm*)	0.121	0.085	0.082	0.089	--
3D volume (*mm^3*)	7.801	3.971	4.289	3.727	3.845
3D absol volume (*mm^3*)	8.421	5.739	4.554	3.732	4.503

BCC lesion cleared at: **V3**

Background, 3D Absol Vol in non-tumor areas:

			MEAN	SD
4.026	6.979	3.458	**4.821**	1.890

Pt 42, Lesion 2
(chest)

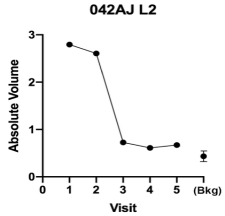

042AJ L2	V1	V2	V3	V4	V5
	Visit Number				
3D diameter (*mm*)	5.32	6.20	3.22	6.006	6.241
3D perp diameter (*mm*)	4.06	4.00	2.97	4.345	3.785
3D av height (*mm*)	0.087	0.076	0.037	0.039	0.035
3D volume (*mm^3*)	2.784	2.591	0.646	0.65	0.604
3D absol volume (*mm^3*)	2.794	2.607	0.727	0.668	0.67

BCC lesion cleared at: **V4**

Background, 3D Absol Vol in non-tumor areas:

			MEAN	SD
0.554	0.61	0.522	**0.562**	0.045

Pt 42, Lesion 5
(chest)

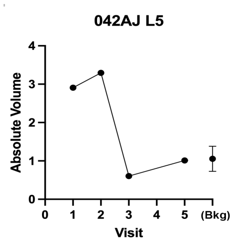

	Visit Number				
**042AJ L5**	**V1**	**V2**	**V3**	**V4**	**V5**
3D diameter (*mm*)	7.36	6.71	3.42		No lesion
3D perp diameter (*mm*)	6.89	6.18	2.43	Photo	visible
3D av height (*mm*)	0.194	0.234	0.064	missing	--
3D volume (*mm^3*)	2.897	3.278	-0.233		0.912
3D absol volume (*mm^3*)	2.907	3.295	0.604		1.011

V3

Background, 3D Absol Vol in non-tumor areas:

			MEAN	SD
1.432	0.837	0.895	**1.055**	0.328

Pt 42, Lesion 3 (chest) DATA NOT USABLE- lesion gone by Visit 2
Pt 42, Lesion 4 (chest) DATA NOT USABLE- lesion gone by Visit 2

Patient / Lesion (Location)	Graphical Summary	3-D lesion data	Visit 1	Visit 2	Visit 3	Visit 4	Visit 5	BCC lesion cleared at:	Biopsy result

Pt 43, Lesion 4A
(ear)

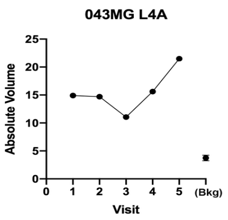

	Visit Number				
**043MG L4 A**	**V1**	**V2**	**V3**	**V4**	**V5**
3D diameter (*mm*)	8.49	7.70	7.40	8.07	9.35
3D perp diameter (*mm*)	6.99	6.94	5.68	7.07	6.17
3D av height (*mm*)	0.414	0.388	0.363	0.399	0.459
3D volume (*mm^3*)	14.816	13.639	11.034	15.312	21.254
3D absol volume (*mm^3*)	14.897	14.706	11.055	15.621	21.498

NC

BCC, Nodular

Background, 3D Absol Vol in non-tumor areas:

			MEAN	SD
4.261	3.28	3.684	**3.742**	0.493

Pt 43, Lesion 4B
(ear)

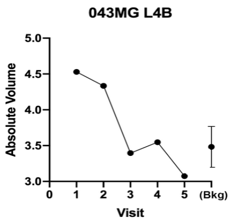

	Visit Number				
**043MG L4 B**	**V1**	**V2**	**V3**	**V4**	**V5**
3D diameter (*mm*)	8.48	8.44	8.79	No lesion	No lesion
3D perp diameter (*mm*)	7.04	6.30	5.03	visible	visible
3D av height (*mm*)	0.041	0.035	0.004	--	--
3D volume (*mm^3*)	-1.048	-2.084	-3.202	-2.903	-2.188
3D absol volume (*mm^3*)	4.529	4.334	3.396	3.548	3.075

V3

Background, 3D Absol Vol in non-tumor areas:

			MEAN	SD
4.261	3.280	3.684	**3.742**	0.493

Pt 43, Lesion 1 (lip) DATA NOT USABLE- sunken biopsy scar, no changes after PDT
Pt 43, Les 2 (forehead) DATA NOT USABLE- sunken biopsy scar, no changes after PDT
Pt 43, Lesion 3 (cheek) DATA NOT USABLE- flat biopsy scar, did not change after PDT
Pt 43, Lesion 5 (scalp) DATA NOT USABLE- biopsy scar, hemorrhagic papule at V2

Patient / Lesion (Location)	Graphical Summary	3-D lesion data	Visit 1	Visit 2	Visit 3	Visit 4	Visit 5	BCC lesion cleared at:	Biopsy result

Patient 44

Pt 44, Lesion 1 (chest) DATA NOT USABLE- tumor already gone at V2
Pt 44, Lesion 2 (clavicle) DATA NOT USABLE- tumor already gone at V2
Pt 44, Lesion 1 (neck) DATA NOT USABLE- tumor already gone at V2

Patient / Lesion (Location)	Graphical Summary	3-D lesion data	Visit 1	Visit 2	Visit 3	Visit 4	Visit 5	BCC lesion cleared at:	Biopsy result

Pt 45, Lesion 1
(shoulder)

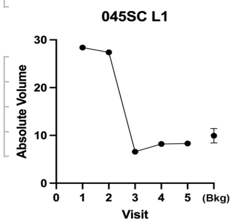

	Visit Number				
**045SC L1**	**V1**	**V2**	**V3**	**V4**	**V5**
3D diameter (*mm*)	18.499	19.704	16.584	17.034	17.866
3D perp diameter (*mm*)	12.901	15.039	8.875	8.903	9.32
3D av height (*mm*)	0.189	0.133	0.063	0.069	0.049
3D volume (*mm^3*)	28.146	25.051	5.825	6.597	5.209
3D absol volume (*mm^3*)	28.385	27.4	6.586	8.211	8.311

V3

Background, 3D Absol Vol in non-tumor areas:

			MEAN	SD
9.833	10.002	9.054	**9.630**	0.506

Pt 45, Lesion 2
(arm)

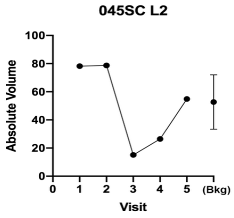

	Visit Number				
**045SC L2**	**V1**	**V2**	**V3**	**V4**	**V5**
3D diameter (*mm*)	24.692		22.932	24.275	23.902
3D perp diameter (*mm*)	20.263		20.045	20.873	21.356
3D av height (*mm*)	0.167		0.12	0.163	0.19
3D volume (*mm^3*)	75.013	73.135	12.263	24.293	54.477
3D absol volume (*mm^3*)	78.238	78.676	15.126	26.478	54.858

V3

Background, 3D Absol Vol in non-tumor areas:

			MEAN	SD
37.60797	46.038	74.598	**52.748**	19.386

Pt 45, Lesion 3
(shoulder)

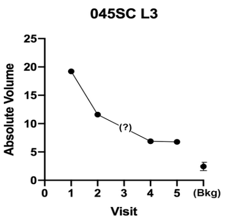

045SC L3	V1	V2	V3	V4	V5
3D diameter (*mm*)	13.124	12.85		Too	8.744
3D perp diameter (*mm*)	9.716	9.662		much	6.784
3D av height (*mm*)	0.227	0.19	Photo	hair	0.178
3D volume (*mm^3*)	18.976	15.423			7.236
3D absol volume (*mm^3*)	19.223	15.652			7.267

Visit Number

NC

BCC, nodular (Biopsied at V5)

Background, 3D Absol Vol in non-tumor areas:

			MEAN	SD
3.035	2.328	1.832	**2.398**	0.605

Pt 45, Lesion 4
(shoulder)

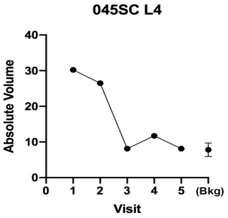

045SC L4	V1	V2	V3	V4	V5
3D diameter (*mm*)	17.796	18.474	15.672	11.732	12.829
3D perp diameter (*mm*)	17.047	16.361	9.148	8.047	8.715
3D av height (*mm*)	0.15	0.117	0.049	0.003	0.029
3D volume (*mm^3*)	27.193	22.288	4.553	0.17	2.347
3D absol volume (*mm^3*)	30.224	26.489	8.115	11.725	8.108

Visit Number

V3

Background, 3D Absol Vol in non-tumor areas:

			MEAN	SD
13.428	8.558	7.067	**9.684**	3.327

Pt 45, Lesion 6
(back)

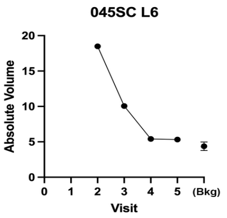

045SC L6	V1	V2	V3	V4	V5
3D diameter (*mm*)		17.012	12.675	13.351	11.712
3D perp diameter (*mm*)		16.358	8.863	8.954	8.562
3D av height (*mm*)		0.025	0.112	0.024	0.039
3D volume (*mm^3*)		4.047	8.168	1.818	2.624
3D absol volume (*mm^3*)		18.499	10.052	5.402	5.329

Visit Number

V4

Background, 3D Absol Vol in non-tumor areas:

			MEAN	SD
3.758	4.981	4.406	**4.382**	0.612

Pt 45, Lesion 7
(back)

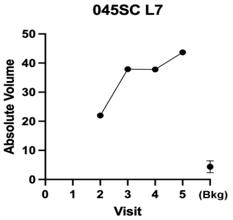

045SC L7	V1	V2	V3	V4	V5
3D diameter (*mm*)		9.845	11.474	11.665	13.281
3D perp diameter (*mm*)		5.858	7.2493	7.2042	8.1183
3D av height (*mm*)		0.526	0.7632	0.1564	0.5458
3D volume (*mm^3*)		21.961	37.556	37.78	43.37
3D absol volume (*mm^3*)		21.967	37.746	37.82	43.69

Visit Number

NC

BCC, Nodular (Biopsied at V5)

Background, 3D Absol Vol in non-tumor areas:

			MEAN	SD
5.156	3.271	4.66	**4.362**	0.977

Pt 45, Lesion 5 (back) DATA NOT USABLE- tumor already gone at V2

Patient / Lesion (Location)	Graphical Summary	3-D lesion data	Visit 1	Visit 2	Visit 3	Visit 4	Visit 5	BCC lesion cleared at:	Biopsy result

Pt 46, Lesion 1
(shoulder)

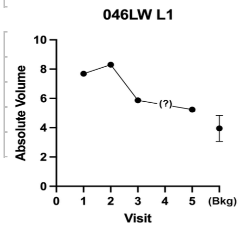

046LW L1	V1	V2	V3	V4	V5
3D diameter (*mm*)	12.664	10.86	11.244	photo	11.67
3D perp diameter (*mm*)	12.333	9.901	9.489	missing	11.35
3D av height (*mm*)	0.065	0.108	0.089		0.065
3D volume (*mm^3*)	6.438	8.063	5.512		12.474
3D absol volume (*mm^3*)	7.691	8.303	5.867		5.241

Visit Number

V5

Background, 3D Absol Vol in non-tumor areas:

			MEAN	SD
3.555	3.336	4.981	**3.957**	0.893

Pt 46, Lesion 2
(shoulder)

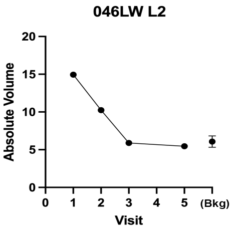

046LW L2	V1	V2	V3	V4	V5
3D diameter (*mm*)	13.179	13.238	13.165		13.32
3D perp diameter (*mm*)	12.07	9.747	9.96	photo	9.579
3D av height (*mm*)	0.153	0.104	0.03	missing	0.046
3D volume (*mm^3*)	14.914	10.16	2.607		4.562
3D absol volume (*mm^3*)	14.944	10.244	5.885		5.455

Visit Number

V3

Background, 3D Absol Vol in non-tumor areas:

			MEAN	SD
5.292	6.791	6.162	**6.082**	0.753

Pt 46, Lesion 4
(back)

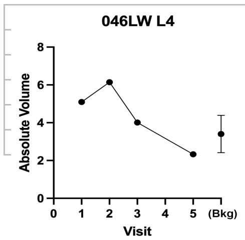

046LW L4	V1	V2	V3	V4	V5
3D diameter (*mm*)	11.79	12.492	13.07		12.124
3D perp diameter (*mm*)	9.757	8.748	8.809	Photo	8.424
3D av height (*mm*)	0.057	0.076	0.098	missing	0.009
3D volume (*mm^3*)	4.408	5.826	3.1		0.675
3D absol volume (*mm^3*)	5.099	6.145	4.01		2.328

Visit Number

V3

Background, 3D Absol Vol in non-tumor areas:

			MEAN	SD
3.958	2.267	3.991	**3.405**	0.986

Pt 46, Les 3 (shoulder) DATA NOT USABLE- sunken scar, tumor already gone at V2

Patient / Lesion (Location)	Graphical Summary	3-D lesion data	Visit 1	Visit 2	Visit 3	Visit 4	Visit 5	BCC lesion cleared at:	Biopsy result

Pt 47, Lesion 1 (leg)

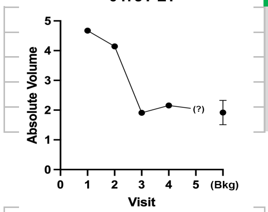

047JT L1	V1	V2	V3	V4	V5
	Visit Number				
3D diameter (*mm*)	9.687	8.647	9.116	9.086	
3D perp diameter (*mm*)	7.888	5.515	5.661	5.798	
3D av height (*mm*)	0.067	0.141	0.046	0.023	
3D volume (*mm^3*)	2.938	4.11	1.424	0.765	photo
3D absol volume (*mm^3*)	4.672	4.144	1.909	2.158	missing

V3

Background, 3D Absol Vol in non-tumor areas:

			MEAN	SD
1.525	2.344	1.886	**1.918**	0.410

Pt 47, Lesion 2A (leg)

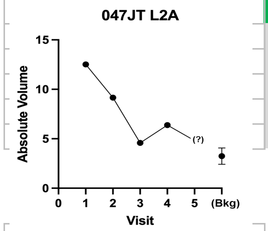

047JT L2A	V1	V2	V3	V4	V5
	Visit Number				
3D diameter (*mm*)	10.068	10.395	10.966	11.276	
3D perp diameter (*mm*)	8.916	7.452	7.634	7.535	
3D av height (*mm*)	0.177	0.158	0.069	0.088	
3D volume (*mm^3*)	11.715	9.042	4.316	5.911	photo
3D absol volume (*mm^3*)	12.513	9.157	4.583	6.383	missing

Inconclusive (no V5)
Did not include in lesion clearance calculations

Background, 3D Absol Vol in non-tumor areas:

			MEAN	SD
2.424	3.235	4.072	**3.244**	0.824

Pt 47, Lesion 2B (leg)

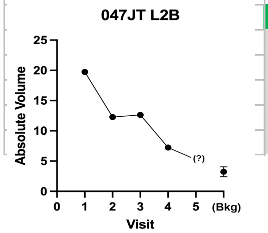

047JT L2B	V1	V2	V3	V4	V5
	Visit Number				
3D diameter (*mm*)	14.537	9.708	10.541	9.836	
3D perp diameter (*mm*)	9.975	7.712	7.3322	7.924	
3D av height (*mm*)	0.187	0.242	0.2679	0.131	
3D volume (*mm^3*)	19.401	12.264	12.544	7.203	photo
3D absol volume (*mm^3*)	19.737	12.286	12.644	7.234	missing

Inconclusive (no V5)
Did not include in lesion clearance calculations

Background, 3D Absol Vol in non-tumor areas:

			MEAN	SD
2.424	3.235	4.072	**3.244**	0.824

Pt 46, Lesion 3 (knee) DATA NOT USABLE- lesion gone at V3, skin has normal epidermal markings

Pt 46, Lesion 4 (leg) DATA NOT USABLE- photos for V5 missing, and some photos may be a different lesion

Patient / Lesion (Location)	Graphical Summary	3-D lesion data	Visit 1	Visit 2	Visit 3	Visit 4	Visit 5	BCC lesion cleared at:	Biopsy result

Patient 48

Pt 48, Lesion 1 (glabella) DATA NOT USABLE- looks like scar, no tumor at V2

Pt 48, Lesion 2 (cheek) DATA NOT USABLE- looks like scar, no tumor at V2

Pt 48, Lesion 3 (back) DATA NOT USABLE- looks like scar, no tumor at V2

Patient / Lesion (Location)	Graphical Summary	3-D lesion data	Visit 1	Visit 2	Visit 3	Visit 4	Visit 5	BCC lesion cleared at:	Biopsy result

Pt 49, Lesion 1 (neck)

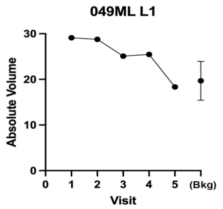

049ML L1	V1	V2	V3	V4	V5
	Visit Number				
3D diameter (*mm*)	24.631	24.147	24.708	25.211	25.148
3D perp diameter (*mm*)	14.008	14.133	13.897	14.435	14.757
3D av height (*mm*)	0.14	0.126	0.051	0.068	0.033
3D volume (*mm^3*)	26.366	22.959	9.895	13.672	6.359
3D absol volume (*mm^3*)	29.123	28.755	25.092	25.471	18.349

V5

Background, 3D Absol Vol in non-tumor areas:

			MEAN	SD
16.344	18.217	24.482	**19.681**	4.262

Pt 49, Lesion 2 (arm

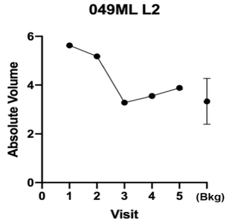

049ML L2	V1	V2	V3	V4	V5
	Visit Number				
3D diameter (*mm*)	10.037	10.263	10.185	9.888	10.425
3D perp diameter (*mm*)	8.834	8.749	8.97	8.893	9.043
3D av height (*mm*)	0.013	0.01	0.002	0.008	0.003
3D volume (*mm^3*)	-4.097	-4.049	-2.995	-2.643	-3.567
3D absol volume (*mm^3*)	5.629	5.176	3.281	3.551	3.881

V3

Background, 3D Absol Vol in non-tumor areas:

			MEAN	SD
2.274	3.652	4.075	**3.334**	0.942

Pt 49, Lesion 4 (back)

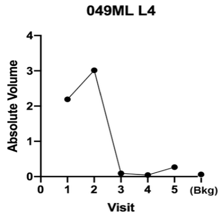

049ML L4	V1	V2	V3	V4	V5
	Visit Number				
3D diameter (*mm*)	4.81	6.45	No lesio	No lesion	No lesion
3D perp diameter (*mm*)	4.72	5.42	visible	visible	visible
3D av height (*mm*)	0.072	0.126	--	--	--
3D volume (*mm^3*)	2.121	3.007	0.037	-0.014	-0.226
3D absol volume (*mm^3*)	2.191	3.014	0.093	0.043	0.270

V3

Background, 3D Absol Vol in non-tumor areas:

			MEAN	SD
0.076	0.064	0.100	**0.080**	0.019

Pt 49, Lesion 3 (back) DATA NOT USABLE- missing photos for V4 and v5

Patient / Lesion (Location)	Graphical Summary	3-D lesion data	Visit 1	Visit 2	Visit 3	Visit 4	Visit 5	BCC lesion cleared at:	Biopsy result

Pt 50, Lesion 1
(chest)

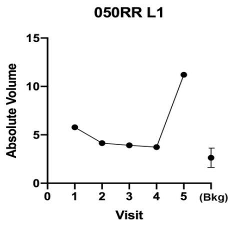

	Visit Number				
050RR L1	V1	V2	V3	V4	V5
3D diameter	12.43	11.64	8.24	8.36	8.12
3D perp diameter	8.96	9.21	7.23	7.51	7.43
3D av height	0.059	0.055	0.080	0.074	0.146
3D volume	5.266	3.952	3.914	3.569	11.150
3D absol volume	5.782	4.138	3.923	3.735	11.206

BCC lesion cleared at: **NC**

Biopsy result: BCC, nodular

Background, 3D Absol Vol in non-tumor areas:

			MEAN	SD
2.466	2.353	1.8	**2.206**	0.356

Pt 50, Lesion 2
(clavicle)

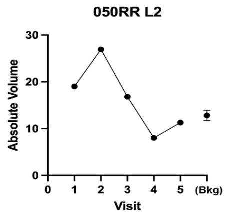

	Visit Number				
050RR L2	V1	V2	V3	V4	V5
3D diameter	30.905	29.472	27.16	No lesion	No lesion
3D perp diameter	16.265	15.629	9.811	visible	visible
3D av height	0.016	0.002	0.014	--	--
3D volume	5.425	0.595	2.418	5.197	1.239
3D absol volume	19.025	26.933	16.817	8.001	11.292

BCC lesion cleared at: **V4**

Background, 3D Absol Vol in non-tumor areas:

			MEAN	SD
12.085	12.262	14.087	**12.811**	1.108

Pt 50, Lesion 3
(shoulder)

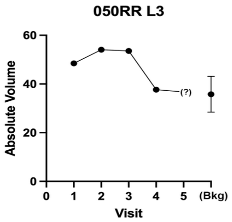

	Visit Number				
050RR L3	V1	V2	V3	V4	V5
3D diameter	18.845	18.905	20.084	19.238	n/d
3D perp diameter	15.418	15.603	16.021	15.697	n/d
3D av height	0.2547	0.279	0.254	0.19	n/d
3D volume	48.419	54.078	53.537	37.671	bad 3D
3D absol volume	48.452	54.085	53.56	37.685	reconstruc

BCC lesion cleared at: **V4**

Background, 3D Absol Vol in non-tumor areas:

			MEAN	SD
31.745	44.298	31.346	**35.796**	7.365

Pt 50, Lesion 4
(shoulder)

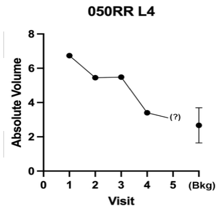

	Visit Number				
050RR L4	V1	V2	V3	V4	V5
3D diameter	12.211	10.868	11.206	11.496	nd
3D perp diameter	7.957	7.105	7.957	8.151	nd
3D av height	0.11	0.092	0.077	0.042	nd
3D volume	6.714	5.243	5.148	2.936	nd
3D absol volume	6.737	5.448	5.481	3.405	nd

BCC lesion cleared at: **V5**

Background, 3D Absol Vol in non-tumor areas:

			MEAN	SD
2.484	1.752	3.775	**2.670**	1.024

Pt 50, Lesion 7
(arm)

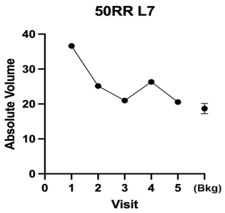

	Visit Number				
050RR L7	V1	V2	V3	V4	V5
3D diameter	15.457	15.451	15.345	15.207	15.15
3D perp diameter	12.778	12.342	12.122	12.242	12.561
3D av height	0.287	0.197	0.167	0.21	0.167
3D Av Ht/Area * 10^4	36.629	25.144	20.951	26.33	20.542
3D volume	36.629	25.146	20.977	26.331	20.55

BCC lesion cleared at: **V5**

Background, 3D Absol Vol in non-tumor areas:

			MEAN	SD
19.102	17.037	19.87	**18.670**	1.465

Pt 50, Lesion 5 (back) DATA NOT USABLE- missing photos for V3

Pt 50, Lesion 6 (back) DATA NOT USABLE- some lesions in photos are mislabelled

SUPPLEMENTARY TABLE S2.

Absolute Tumor Volume (3DAbsVol) as a Predictor of BCC Tumor Clearance after PDT: Calculation of Sensitivity and Specificity

BCC tumors that resolved after 1, 2 or 3 PDT sesions					
		Absolute Volume (mm^3)			
**Subject #**	**Lesion #**	Visit 1	Visit 2	MeanV1V2	
9	10	0.565	0.458	0.511	
6	2	0.642	1.353	0.998	
2	2	1.358	0.719	1.038	
35	1	1.017	1.733	1.375	
3	1	1.424		1.424	
9	3	1.964	1.317	1.641	
9	5	2.231	1.123	1.677	
34	1	1.132	2.321	1.727	
2	5	1.768	2.086	1.927	
35	4	2.768	1.607	2.187	
12	6	2.341	2.230	2.285	
31	3	2.078	2.545	2.312	
12	3	2.880	1.784	2.332	
9	7	2.327	2.348	2.338	
3	2	1.950	3.042	2.496	
10	5		2.498	2.498	
14	2	2.660	2.406	2.533	
3	5	2.423	2.674	2.549	
49	4	2.191	3.014	2.603	
42	2	2.794	2.607	2.701	
8	4	3.340	2.731	3.035	
42	5	2.907	3.295	3.101	
12	2	3.544	2.828	3.186	
36	3	4.169	3.149	3.659	
12	10	4.855	2.890	3.872	
3	3	3.943	4.294	4.118	
47	1	4.672	4.144	4.408	
43	4B	4.529	4.334	4.432	
36	2	4.942	4.098	4.520	
14	1	8.075	1.664	4.870	
9	8	4.091	5.701	4.896	
10	1	6.212	3.676	4.944	
12	4	5.410	5.132	5.271	
14	3	4.396	6.235	5.316	
49	2	5.629	5.176	5.403	
3	6	6.352	4.466	5.409	
6	3	4.562	6.422	5.492	
8	5		5.597	5.597	
46	4	5.099	6.145	5.622	
50	4	6.737	5.448	6.093	
3	4	6.510	5.890	6.200	
5	3	6.535	6.505	6.520	
42	1	8.421	5.739	7.080	
8	3	6.660	8.027	7.343	
46	1	7.691	8.303	7.997	
5	1	7.764	8.348	8.056	
14	4	8.316	8.651	8.484	
39	2		8.610	8.610	
12	7	10.346	7.238	8.792	
14	5	11.451	6.280	8.866	
38	1	9.529	8.258	8.894	
7	2	10.512	8.897	9.704	
12	9	12.498	7.057	9.778	
10	3	10.551	10.199	10.375	
7	1	9.169	11.761	10.465	
14	7	8.849	12.648	10.749	
12	1	11.767	9.815	10.791	
12	5	13.617	8.216	10.916	
6	4	13.372	8.463	10.917	59
46	2	14.944	10.244	12.594	
6	5	10.902	15.025	12.964	

BCC tumors that failed to clear after 3 PDT sessions						Histology of the Nonresponders
		Absolute Volume (mm^3)				
**Subject #**	**Lesion #**	**Visit 1**	**Visit 2**	MeanV1V2		
5	8	1.561	1.106	1.334		mnBCC
9	9	1.857	1.091	1.474		sBCC
15	4	1.722	1.963	1.843		o
6	1	2.996	2.526	2.761		adenBCC
1	5	3.029	3.127	3.078		mnBCC
15	2	3.040	4.076	3.558		o
5	6	4.038	4.421	4.229		infBCC
50	1	5.782	4.138	4.960		nBCC
9	2	6.599	3.897	5.248		mnBCC
9	4	5.398	6.287	5.842		mnBCC
15	3	10.030	5.690	7.860		TrichoBCC
1	1		8.314	8.314		o
1	3	7.905	8.940	8.423		mnBCC
15	5	8.734	8.227	8.481		o
5	7	7.069	10.128	8.599		adenBCC
41	1		10.104	10.104		nBCC
15	9	9.730	11.630	10.680	17	mnBCC
14	6	12.152		12.152		mnBCC
15	10	12.190	13.360	12.775		TRichoBCC
8	6		14.468	14.468		o
43	4	14.897	14.706	14.802		nBCC
45	3	19.223	15.652	17.438		nBCC
14	9	22.369	13.422	17.896		TrichoBCC
15	6	18.582	19.798	19.190		o
45	7		21.967	21.967		nBCC
5	9	27.065	17.004	22.035		mnBCC
14	10	19.870	26.030	22.950		o
39	1	25.628		25.628		nBCC
41	5		33.578	33.578		o
15	7	40.915	52.705	46.810		o
15	1	52.803	50.639	51.721		o
9	6	51.243	57.343	54.293		mnBCC
5	4	82.831	66.033	74.432		nBCC
5	5	50.306	105.462	77.884		infBCC
9	1	106.636	72.914	89.775		nBCC
41	3		108.494	108.494	19	nBCC

ANALYSIS 1:

Use 3DAbsVol = 12.0 cubic mm to predict whether BCC lesions clear with PDT:

Total sample population:

P = positives = lesions that cleared with PDT; P = 86

N = negatives = lesions that failed to clear; N = 36 P + N = 122 lesions

True positives (Actually cleared; predicted to clear): 59

False positives (NC; predicted to clear): 17

True negative (NC; predicted NC): 19

False negative (Actually cleared; predicted NC): 27

NC = not cleared

		PREDICTED CONDITION		
		**predict positive**	**predict negative**	
ACTUAL CONDITION	**Lesion cleared**	True positive TP = 59	False negative FN = 27	86
	**Failed to clear**	False positive FP = 17	True negative TN = 19	36

SENSITIVITY (or true positive rate) = TP /P = 59/8[illegible] **68.60%**

SPECIFICITY (or true negative rate) = TN /N = 19/3[illegible] **52.78%**

Subject #	Lesion #	(Absolute Volume, continued)			
41	4	16.890	9.041	12.966	
10	2	15.372	10.692	13.032	
28	3	12.887	16.600	14.744	
5	10	14.443	17.244	15.844	
1	6	16.801	15.882	16.341	
15	8	18.230	15.860	17.045	
7	3	18.445	16.947	17.696	
2	1	24.558	12.326	18.442	
45	6		18.499	18.499	
12	8	21.305	15.753	18.529	
41	2	27.482	16.424	21.953	
50	2	19.025	26.933	22.979	
5	2	22.260	25.737	23.999	
45	1	28.385	27.400	27.893	
45	4	30.224	26.489	28.357	
49	1	29.123	28.755	28.939	
36	1	26.886	31.257	29.071	
14	8	24.727	35.987	30.357	
50	7	36.629	25.146	30.888	
1	4	43.334	18.573	30.954	
50	3	48.452	27.040	37.746	
1	2	44.856	50.176	47.516	
38	3	64.608		64.608	
38	2	60.787	76.320	68.553	
45	2	78.238	78.676	78.457	27

ANALYSIS 2:

The specifity may be too low because many true negatives were BCC tumors with aggressive histological subtypes. In clinical practice, such aggressive tumors would not be chosen for PDT after a diagnostic biopsy.

Question:

What happens if only the nonresponders with histology of sBCC or nBCC are included in the analysis?

PREDICTED CONDITION

ACTUAL CONDITION		predict positive	predict negative	
	**Lesion cleared**	True positive TP = 59	False negative FN = 27	86
	**Failed to clear**	False positive FP = 3	True negative TN = 7	10

SENSITIVITY (or true positive rate) = TP /P =	59/8(	**68.60%**
SPECIFICITY (or true negative rate) = TN /N =	7/10	**70.00%**

Result: Sensitivity and specificity are nearly the same.

SUPPLEMENTARY TABLE S3.

Average Tumor Height (3DAvHt) as a Predictor of BCC Tumor Clearance after PDT: Calculation of Sensitivity and Specificity

BCC tumors that resolved after 1, 2 or 3 PDT sesions

		**Average Tumor Height (mm)**		
**Subject #**	**Lesion #**	Visit 1	Visit 2	MeanV1V2
3	1	0.004		**0.004**
3	4	0.005	0.006	**0.006**
6	2	0.010	0.001	**0.006**
6	5	0.009	0.003	**0.006**
14	3	0.006	0.008	**0.007**
28	3	0.015	0.002	**0.009**
50	2	0.016	0.002	**0.009**
12	8	0.006	0.015	**0.011**
49	2	0.013	0.01	**0.012**
12	7	0.015	0.018	**0.017**
12	6	0.015	0.018	**0.017**
9	10	0.026	0.010	**0.018**
5	3	0.022	0.016	**0.019**
5	2	0.012	0.030	**0.021**
9	3	0.021	0.022	**0.022**
9	8	0.025	0.021	**0.023**
45	6		0.025	**0.025**
34	1	0.024	0.029	**0.027**
35	1	0.024	0.029	**0.027**
36	3	0.032	0.025	**0.029**
12	4	0.047	0.011	**0.029**
2	2	0.047	0.016	**0.032**
12	1	0.045	0.030	**0.037**
43	4B	0.041	0.035	**0.038**
12	2	0.038	0.040	**0.039**
12	3	0.045	0.033	**0.039**
14	2	0.036	0.044	**0.040**
10	2	0.019	0.063	**0.041**
36	2	0.038	0.044	**0.041**
8	5		0.042	**0.042**
1	6	0.047	0.039	**0.043**
10	1	0.068	0.023	**0.046**
8	4	0.048	0.047	**0.047**
12	5	0.071	0.027	**0.049**
6	4	0.051	0.048	**0.050**
5	10	0.076	0.026	**0.051**
3	3	0.040	0.063	**0.051**
31	3	0.051	0.059	**0.055**
3	5	0.053	0.061	**0.057**
10	3	0.072	0.046	**0.059**
1	4	0.071	0.052	**0.062**
7	1	0.063	0.066	**0.064**
46	4	0.057	0.076	**0.067**
5	1	0.067	0.072	**0.069**
9	5	0.101	0.047	**0.074**
39	2		0.074	**0.074**
12	10	0.100	0.049	**0.074**
6	3	0.067	0.088	**0.077**
36	1	0.073	0.083	**0.078**
14	8	0.027	0.134	**0.081**
42	2	0.087	0.076	**0.082**
46	1	0.065	0.108	**0.087**
10	5		0.089	**0.089**
41	2		0.091	**0.091**
14	5	0.116	0.071	**0.094**
15	8	0.110	0.088	**0.099**
49	4	0.072	0.126	**0.099**
50	4	0.11	0.092	**0.101**
7	3	0.126	0.079	**0.102**
42	1	0.121	0.085	**0.103**
47	1	0.067	0.141	**0.104**

BCC tumors that failed to clear after 3 PDT sessions

		**Average Tumor Height (mm)**				Histology of the Nonresponders	
**Subject #**	**Lesion #**	**Visit 1**	**Visit 2**	MeanV1V2			
5	8	0.045	0.026	0.035			mnBCC
1	1		0.044	0.044			o
9	9	0.061	0.031	0.046		sBCC	
50	1	0.059	0.055	0.057		nBCC	
1	5	0.058	0.060	0.059			mnBCC
5	4	0.060	0.071	0.065		nBCC	
6	1	0.067	0.102	0.085			adenBCC
9	2	0.105	0.064	0.085			mnBCC
1	3	0.081	0.100	0.091			mnBCC
5	7	0.060	0.166	0.113			adenBCC
5	9	0.164	0.101	0.132			infBCC
5	6	0.124	0.142	0.133			infBCC
15	3	0.178	0.093	0.136	13		TrichoBCC
15	2	0.113	0.212	**0.163**			o
41	1		0.168	**0.168**		nBCC	
5	5	0.079	0.272	**0.175**			infBCC
14	9	0.216	0.191	**0.204**			TrichoBCC
45	3	0.227	0.19	**0.209**		nBCC	
15	4	0.203	0.242	**0.223**			o
15	9	0.200	0.248	**0.224**			mnBCC
14	6	0.254		**0.254**			mnBCC
9	4	0.240	0.314	**0.277**			mnBCC
15	10	0.283	0.350	**0.317**			TRichoBCC
14	10	0.297	0.378	**0.338**			o
41	3		0.349	**0.349**		nBCC	
43	4	0.414	0.388	**0.401**		nBCC	
15	5	0.426	0.408	**0.417**			o
41	5		0.501	**0.501**			o
45	7		0.526	**0.526**		nBCC	
9	6	0.549	0.611	**0.580**			mnBCC
15	6	0.661	0.568	**0.615**			o
9	1		0.615	**0.615**		nBCC	
39	1	0.727		**0.727**		nBCC	
15	7	0.694	0.850	**0.772**			o
15	1	0.928	0.848	**0.888**	22		o

ANALYSIS 1:

Use 3DAbsVol = 12.0 cubic mm to predict whether BCC lesions clear with PDT:

Total sample population:

P = positives = lesions that cleared with PDT; P = 86

N = negatives = lesions that failed to clear; N = 35 P + N = 121 lesions

True positives (Actually cleared; predicted to clear): 70

False positives (NC; predicted to clear): 13

True negative (NC; predicted NC): 22

False negative (Actually cleared; predicted NC): 16

NC = not cleared

		PREDICTED CONDITION		
		**predict positive**	**predict negative**	
ACTUAL CONDITION	**Lesion cleared**	True positive TP = 70	False negative FN = 16	86
	**Failed to clear**	False positive FP = 13	True negative TN = 22	35

SENSITIVITY (or true positive rate) = TP /P = 70/86 **81.40%**

SPECIFICITY (or true negative rate) = TN /N = 22/35 **62.86%**

Subject #	Lesion #	Average Tumor Height (mm)			
35	4	0.095	0.136	**0.115**	
14	7	0.095	0.144	**0.120**	
1	2	0.058	0.198	**0.128**	
46	2	0.153	0.104	**0.129**	
12	9	0.160	0.097	**0.129**	
49	1	0.14	0.126	**0.133**	
45	4	0.15	0.117	**0.134**	
3	2	0.143	0.134	**0.139**	
38	1	0.173	0.121	**0.147**	70
45	1	0.189	0.133	0.161	
14	1	0.198	0.124	0.161	
45	2	0.167		0.167	
3	6	0.200	0.147	0.173	
9	7	0.177	0.185	0.181	
8	3	0.185	0.219	0.202	
2	1	0.392	0.017	0.204	
42	5	0.194	0.234	0.214	
14	4	0.214	0.256	0.235	
50	7	0.287	0.197	0.242	
2	5	0.242	0.260	0.251	
41	4	0.328	0.184	0.256	
50	3	0.255	0.279	0.267	
7	2	0.278	0.261	0.270	
38	2	0.278	0.282	0.280	
38	3	0.338		0.338	16

ANALYSIS 2:

The specifity may be too low because many true negatives were BCC tumors with aggressive histological subtypes. In clinical practice, such aggressive tumors would not be chosen for PDT after a diagnostic biopsy.

Question:

What happens if only the nonresponders with histology of sBCC or nBCC are included in the analysis?

ACTUAL CONDITION		PREDICTED CONDITION: **predict positive**	**predict negative**	
	**Lesion cleared**	True positive<br>TP = 70	False negative<br>FN = 16	86
	**Failed to clear**	False positive<br>FP = 3	True negative<br>TN = 7	10

SENSITIVITY (or true positive rate) = TP /P = 70/86 **81.40%**

SPECIFICITY (or true negative rate) = TN /N = 7/10 = **70.00%**

Result: Sensitivity and specificity are more similar than in Analysis 1.

SUPPLEMENTARY TABLE S4. Comparison of BCC histological subtype, measured tumor depth (H&E), and the calculated 3D height (3DAvHt)

These are Depth-of-invasion (DOI) measurements of BCC tumors from H&E stained sections of PDT-resistant tumors, stained with H&E and digitized on a Leica scanner. The distance from the outer epidermis to the bottom of the tumor was measured using Q-Path software. Depths from 10 different locations per tumor were averaged.

Index Number	Histological subtype category *	Original histological diagnosis by pathologist	Average DOI (microns)	3DAvHeight (mm)
1	superficial	BCC, superficial	129.433	0.025
		mean DOI:	129	0.025
2	nodular	BCC, nodular	354.517	0.144
3	nodular	BCC, nodular	245.187	0.138
4	nodular	BCC, nodular	1672.2	0.282
5	nodular	BCC, nodular	564.852	
6	nodular	BCC, superficial and nodular	417.687	
7	nodular	BCC, nodular	1351.32	
		mean DOI:	767.6	0.188
		SD	594.3	0.081
		SEM	**242.6**	**0.047**
8	micronodular	BCC, nodular and microndodular	832.511	0.166
9	micronodular	BCC, nodular and microndodular	2186.77	0.167
10	micronodular	BCC, micronodular	832.149	0.085
11	micronod	BCC, nod, micronod, adenoid	612.499	0.140
12	micronodular	BCC, nodular and microndodular	1548.5	0.102
13	micronodular	BCC, nodular and microndodular	2039.892	0.168
14	micronodular	BCC, micronodular	3080.48	0.473
15	micronodular	BCC, superficial, nod, micronod	1173.533	0.608
16	micronodular	BCC, nodular and micronodular	715.136	
17	micronodular	BCC, nodular and micronodular	795.158	
18	micronodular	BCC, micronodular	307.276	
19	micronodular	BCC, superficial, nod, micronod	966.945	0.134
		mean DOI:	1258	0.227
		SD	807.0	0.183
		SEM	**233.0**	**0.061**
20	infiltrative	BCC, infiltrative	3090.58	0.318
21	infiltrative	BCC, nodular and infiltrative	1268.101	0.315
22	infiltrative	BCC, micronodular & infiltrative	308.343	0.343
		mean DOI:	1556	0.325
		SD	1413.2	0.015
		SEM	**815.9**	**0.009**
	"Other"			
23	adenoid	BCC, nodular and adenoid	1432.455	0.067
24	trichoepithelial	BCC, superf, nod, trichoepithelial	1122.227	0.284
25	trichoepithelial	BCC, superf, nod, trichoepithelial	1183.094	0.223
26	trichoepithelial	BCC, superf, nod, trichoepithelial	767.352	0.243
		mean DOI:	1126	0.204
		SD	274.4	0.095
		SEM	**137.2**	**0.047**

(Data for Graph A)

Average Tumor Depth vs. Histologic subtype

Depth (microns)

Histo. Subtype*	mean	SEM	n
sBCC	129	0	1
nBCC	767	242	6
mnBCC	1258	233	12
infBCC	1556	815	3
otherBCC	1126	137	4

GRAPH A

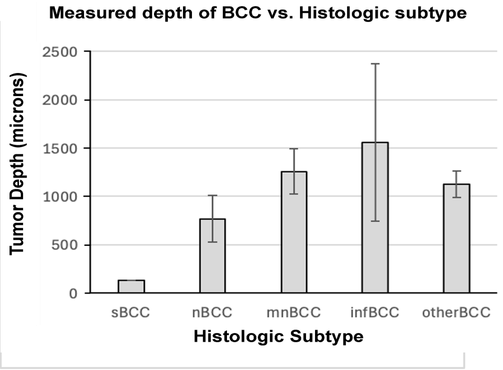

(Data for Graph B)

Calculated 3D Height vs. Histologic Subtype

3DAvHt (mm)

Subtype	mean	SEM	n
sBCC	0.025	0	1
nBCC	0.188	0.047	3
mnBCC	0.227	0.061	8
infBCC	0.325	0.009	3
otherBCC	0.204	0.047	4

GRAPH B

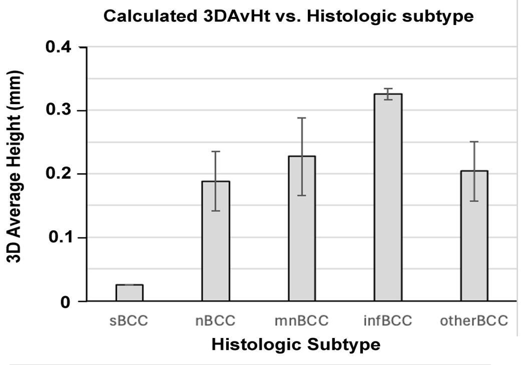

Footnotes:

* The most biologically aggressive subtype mentioned by the pathologists was chosen as the histologic category, using the following hierarchy: infiltrative, "other" > micronodular > nodular > superficial
